# Energy landscapes and synergetic state transitions in frustrated Stuart–Landau oscillator networks: a homotopy continuation study

**DOI:** 10.3389/fnetp.2026.1778380

**Published:** 2026-06-26

**Authors:** Yang Li, Kazuyuki Aihara

**Affiliations:** International Research Center for Neurointelligence (WPI-IRCN), The University of Tokyo, Tokyo, Japan

**Keywords:** energy landscape, energy-based models, homotopy continuation, multistability, network physiology, state transitions, Stuart–Landau oscillator networks, synergetics

## Abstract

**Introduction:**

Energy landscapes provide a useful lens for understanding multistability and state transitions in self-organizing systems, but systematic characterization of the energy landscapes for coupled oscillator networks remains less well developed. Here, we study a frustrated Stuart–Landau oscillator network on a 2D toroidal lattice with competing local coupling and global frustration and characterize how its macroscopic states and noise-driven transitions reorganize as the frustration strength is varied.

**Method:**

We combine a mode-based, phase-decoupled approximation with homotopy continuation and track representative families of equilibria from the approximation to the full model. In singular cases where the leading XY-Hamiltonian phase interaction yields non-isolated critical points, we impose a second-order phase equilibrium condition and solve it in a constrained-solvability sense to ensure that continuation is well posed.

**Results:**

Numerical continuation shows partial selectivity of this homotopy, in which lower-energy initializations tend to continue to similarly lower-energy, lower-index equilibria in the full system. Across frustration regimes, continuation shows how approximate equilibria split and reorder by energy and index; at intermediate frustration, extensive runs reveal multistability between an isolated global minimum and multiple 1D troughs contained within the edges of a multigraph. Guided by the resulting landscape-level picture, simulations demonstrate distinct noise-dependent synergetic phenomena, including noise-induced synchronization, noise-induced desynchronization, and persistent switching among coexisting macrostates. Stationary transition kinetics exhibit both Arrhenius–Kramers-like (activated) and diffusion-limited scaling with respect to noise.

**Discussion:**

These results support a coupled-oscillator-based framework for analyzing the energy landscape of multivariate time series, with potential applications in neuroscience, physiology, and beyond.

## 1 Introduction

Many natural and artificial systems spontaneously develop ordered and coherent patterns without centralized control. Synergetics, pioneered by Hermann Haken (Haken, 1977, Haken, 1983b, Haken, 1989), provides a conceptual framework for such self-organization by emphasizing how macroscopic structures emerge from the collective dynamics of many interacting components. A central message is that, near a qualitative reorganization of behavior, the high-dimensional dynamics of a complex system can often be captured by a small set of collective variables (i.e., *order parameters*), while the remaining degrees of freedom are effectively *slaved* to them. In this view, abrupt changes in global behavior are driven by shifts in *control parameters* that reshape the accessible macroscopic states and their stability, and *fluctuations* such as noise can trigger transitions among coexisting attractors.

Coupled oscillator networks constitute a prototypical arena for synergetics (Haken, 1983a): simple rhythmic units interact locally yet generate global synchronization, patterned phase-locking, and complex spatiotemporal oscillations (Strogatz, 2000; Strogatz, 2003). In some oscillator networks, collective dynamics can be organized by an *energy* or *potential*-like function, yielding a landscape-level representation of multistability and state transitions. From a synergetics perspective, the energy landscape provides a natural interpretive lens: stable macroscopic patterns correspond to minima, while unstable critical points and barriers shape the transition structure; noise can induce switching by enabling escapes across basins, and control parameters can deform the landscape, creating, destroying, or reshaping its structure. This viewpoint is especially valuable when one aims to identify and compare multiple coexisting collective states rather than focusing on a single attractor.

Recently, energy-based dynamical models and coupled oscillator networks have gained renewed attention as mechanistic models of interacting physiological systems, particularly for large-scale brain activity (Schneidman et al., 2006; Fraiman et al., 2009; Watanabe et al., 2013; Deco et al., 2017; Deco and Kringelbach, 2020; Chinichian et al., 2024; Hannay et al., 2018; Smart et al., 2023). The brain exhibits rich rhythms that may support information processing across perception, cognition, and memory (Buzsáki and Draguhn, 2004; Fries, 2005; Fries, 2015; Buzsáki and Mihály, 2023), and the coexistence of multiple metastable states provides a principled framework for discussing flexible switching among brain states (Tognoli and Kelso, 2014; Adachi and Aihara, 1997; Tsuda, 2015; Deco et al., 2019). Spin-based energy models have been influential in formalizing such metastability in terms of energy landscapes (Watanabe et al., 2013; Watanabe et al., 2014; Ezaki et al., 2017; Masuda et al., 2025). More recently, their application to health checkup data has identified multiple states and different state transitions in diabetes progression, highlighting their potential across a broader range of physiological systems (Ito et al., 2025). Meanwhile, coupled oscillator models offer an intrinsically continuous and rhythmic alternative that can express a broader repertoire of collective dynamics. These perspectives motivate studying *energy landscapes in coupled oscillator networks* to clarify how stable rhythmic states are organized and how noise or other possible mechanisms modulate transitions between them, as well as their potential application in networked physiology.

In this article, we investigate the energy landscape of a frustrated Stuart–Landau oscillator network and its noise-driven transitions among coexisting macroscopic states, as the frustration strength is varied across different regimes. Methodologically, we begin with an analytically tractable mode-based, phase-decoupled approximation, which suggests potentially dominant coordinates for macroscopic states; we then use *homotopy continuation* (Chen and Li, 2015) to track representative families of equilibria from the approximation to the full system while monitoring their energies and stability indices. In the singular cases in which the phase critical points of the initial XY-Hamiltonian-like term are non-isolated, we impose a second-order phase equilibrium condition and solve it in a *constrained-solvability* sense to make the continuation well posed. Extensive continuation runs reveal a *partial selectivity* property: an initial equilibrium with relatively high (or low) energy and stability index under the approximation tends to continue to equilibria with similarly high (or low) energy and indices in the full system. This continuation-based exploration yields a quantitative, landscape-level characterization of the typical macrostates that appear and of how their stability and energetic ordering change across frustration regimes. At an intermediate level of frustration, the energy landscape of the network exhibits an isolated global minimum and multiple 1D equi-energy troughs supported on the edges of a multigraph; for sufficiently large frustration, these troughs extend to the entire multigraph, which then constitutes the set of global minima. Based on this characterization, we demonstrate distinct noise-dependent phenomena, including noise-induced synchronization, desynchronization, and switching among macrostates; moreover, as functions of the noise level, we observe Arrhenius–Kramers-like scaling for uphill activation and diffusion-limited scaling for motion along the graph. Our results serve as a step toward an *oscillator-based* energy landscape analysis framework for real multivariate time series data, with potential applications in neuroscience, networked physiology, and beyond.

## 2 Model and methods

### 2.1 Stuart–Landau oscillator network: an energy-based model

We consider a network of N Stuart–Landau oscillators, with a complex-valued state $s_{i}$ of the i th oscillator, whose dynamics is given by

$$\frac{ds_{i}}{dt}=s_{i}\left[\left(a_{i}+ib\right)-|s_{i}{|}^{2}\right]+\overset{N}{\underset{j=1}{\sum }}w_{ij}s_{j}+\sigma \frac{dB_{i}}{dt},$$ (1)

for i=1,2,…,N . Here, $a_{i}$ and b are real constants that modulate the local dynamics of a single oscillator, which undergoes a supercritical Hopf bifurcation at $a_{i}=0$ and creates a stable limit cycle $s_{i}\left(t\right)=\sqrt{a_{i}}\exp\left(ibt\right)$ for $a_{i}>0$ ; $B_{i}$ represents complex-valued standard Brownian motion, and for simplicity, we assume independent, identically distributed (i.i.d.) noise across oscillators with intensity σ . The oscillators are linearly coupled *via* a real symmetric matrix $W=\left[w_{ij}\right]$ with zero diagonal entries because any $w_{ii}\neq 0$ should be merged with $a_{i}$ . The deterministic part of Equation 1 can also be expressed in polar coordinates, with $s_{i}=\rho_{i}\exp\left(i\phi_{i}\right)$ , as

$$\left\{\begin{array}{cc} \frac{d\rho_{i}}{dt} & =\rho_{i}\left(a_{i}-\rho_{i}^{2}\right)+\overset{N}{\underset{j=1}{\sum }}w_{ij}\rho_{j}\cos\left(\phi_{j}-\phi_{i}\right),\\ \rho_{i}\frac{d\phi_{i}}{dt} & =b\rho_{i}+\overset{N}{\underset{j=1}{\sum }}w_{ij}\rho_{j}\sin\left(\phi_{j}-\phi_{i}\right). \end{array}\right.$$ (2)

Note that we include $\rho_{i}$ explicitly on the left-hand side to keep the equation well defined at the phase singularity $\rho_{i}=0$ , where $\rho_{i}d\phi_{i}/dt$ can be interpreted as the velocity component perpendicular to the radial axis. Moreover, Equation 2 remains invariant under the transformation $\left(\rho_{i},\phi_{i}\right)\mapsto \left(-\rho_{i},\phi_{i}\pm \pi \right)$ . In practice, we therefore allow $\rho_{i}$ to take negative values so that $\left(\rho_{i},\phi_{i}\right)$ varies continuously across the origin. We impose the conventional restriction $\rho_{i}\in \left.\left[0,+\infty \right.\right)$ and $\phi_{i}\in \left.\left[-\pi ,\pi \right.\right)$ only when a unique representation is needed.

The local dynamics in Equation 1 is a special case of the general Stuart–Landau form, in which the cubic term $s_{i}|s_{i}{|}^{2}$ may have a complex coefficient (Deco and Kringelbach, 2020; Uchiyama and Fujisaka, 2004). It is also a reduced version of the computational brain models used by Deco et al. (2017, 2019) and Deco and Kringelbach (2020), in which we use a common intrinsic angular frequency b for all oscillators instead of a distribution. A third simplification is the symmetric coupling matrix W . Under these simplifications, the deterministic dynamics in Equation 1 admits an energy-descent description in a co-rotating frame: letting $\phi_{i}\mapsto \phi_{i}+bt$ (i=1,2,…,N) , the dynamics reduces to the case b=0 , where it becomes a gradient flow as follows:

$$\left\{\begin{array}{cc} \frac{d\rho_{i}}{dt} & =-\frac{\partial H}{\partial \rho_{i}},\\ \rho_{i}\frac{d\phi_{i}}{dt} & =-\frac{\partial H}{\rho_{i}\partial \phi_{i}}, \end{array}\right.$$ (3)

for i=1,2,…,N , with the energy function given by Equation 4:

$$H\left(\rho ,\phi \right)=\frac{1}{4}\overset{N}{\underset{i=1}{\sum }}\rho_{i}^{4}-\frac{1}{2}\overset{N}{\underset{i=1}{\sum }}a_{i}\rho_{i}^{2}-\overset{N}{\underset{i=1}{\sum }}\underset{j<i}{\sum }\rho_{i}\rho_{j}w_{ij}\cos\left(\phi_{i}-\phi_{j}\right).$$ (4)

For notation in this article, we collect state variables in vector forms such as $\rho ={\left[\rho_{1}\;\rho_{2}\;\cdots \;\rho_{N}\right]}^{⊤}$ and $\phi ={\left[\phi_{1}\;\phi_{2}\;\cdots \;\phi_{N}\right]}^{⊤}$ ; for a scalar like H , gradients such as ∂H/∂ρ and ∂H/∂ϕ lie in $R^{N}$ , and derivatives of a vector with respect to another such as $\partial^{2}H/\partial \rho^{2}$ and $\partial^{2}H/\left(\partial \phi \partial \rho \right)$ denote the corresponding Jacobian matrices (Hessian blocks for H ). In addition, we write ◦ for elementwise (Hadamard) products and use the shorthand ∂H/(ρ∂ϕ)≔(1/ρ)◦(∂H/∂ϕ) . Then, along trajectories of Equation 3, we have:

$$\frac{\;dH}{\;dt}={\left(\frac{\partial H}{\partial \rho }\right)}^{⊤}\frac{\;d\rho }{\;dt}+{\left(\frac{\partial H}{\rho \partial \phi }\right)}^{⊤}\left(\rho ◦\frac{\;d\phi }{\;dt}\right)=-{\left\|\frac{\partial H}{\partial \rho }\right\|}^{2}-{\left\|\frac{\partial H}{\rho \partial \phi }\right\|}^{2}\leq 0,$$ (5)

where the last equality holds if and only if ∂H/∂ρ=∂H/(ρ∂ϕ)=0 . Thus, as indicated by Equation 5, the deterministic system in the co-rotating frame relaxes, with monotonically decreasing H , toward an equilibrium of Equation 3, or equivalently, a critical point of H(ρ,ϕ) . Returning to the original frame, the network described by Equation 2 always converges to a frequency-synchronized solution with common angular frequency b . Accordingly, throughout the article, we focus on the gradient case b=0 and refer to H(ϕ,ρ) as the energy function for the coupled oscillator network described by Equation 1, regardless of the value of b .

The energy function H can have a complicated critical-point structure, which depends on system parameters, most notably the coupling matrix W . Generally speaking, the equilibria of a gradient system may be *isolated* or *non-isolated*; in the latter case, they form a connected critical manifold along which the energy landscape is locally flat. Correspondingly, isolated equilibria can be asymptotically *stable* (local minima of H ) or *unstable* (saddles or maxima). In contrast, non-isolated equilibria are typically *semi-stable* due to neutral directions tangent to the critical set. These properties are largely captured by the eigenvalue spectrum of an appropriate Hessian of H evaluated at the equilibrium. In polar coordinates, we adopt the following Hessian convention:

$$\nabla^{2}H\left(\rho ,\phi \right)=\left[\begin{array}{cc} \frac{\partial^{2}H}{\partial \rho^{2}} & \frac{\partial^{2}H}{\rho \partial \phi \partial \rho }-\text{Diag }\left(\frac{\partial H}{\rho^{2}\partial \phi }\right)\\ \frac{\partial^{2}H}{\rho \partial \rho \partial \phi }-\text{Diag }\left(\frac{\partial H}{\rho^{2}\partial \phi }\right) & \frac{\partial^{2}H}{\rho^{2}\partial \phi^{2}}+\text{Diag }\left(\frac{\partial H}{\rho \partial \rho }\right) \end{array}\right]$$ (6)

so that the second-order Taylor expansion takes the form:

$$\frac{1}{2}{\left[\begin{array}{c} \delta \rho \\ \rho ◦\delta \phi \end{array}\right]}^{⊤}\nabla^{2}H\left(\rho ,\phi \right)\left[\begin{array}{c} \delta \rho \\ \rho ◦\delta \phi \end{array}\right],$$

which remains meaningful at the phase singularity $\rho_{i}=0$ . At a critical point of H(ρ,ϕ) , a positive definite $\nabla^{2}H$ implies an isolated minimum (a stable equilibrium), while negative eigenvalues of $\nabla^{2}H$ indicate unstable directions. Zero eigenvalues signal neutral directions and may indicate either a genuine critical manifold of non-isolated equilibria or an isolated minimum, maximum, or saddle point whose type generally must be determined by higher-order analysis. For the system given by Equation 3, the energy function in Equation 4 is invariant under the global phase shift ϕ↦ϕ+ε1 . Consequently, any nontrivial equilibrium with ρ≠0 is non-isolated in the direction δϕ=1 , yielding a zero eigenvalue of $\nabla^{2}H$ . We exclude this symmetry-induced degree of freedom and assess stability considering the remaining 2N−1 eigenvalues; if they are all positive, the associated frequency-synchronized periodic orbit of Equation 2 is deemed a local minimum. In practice, we define the *index* of an equilibrium as the number of negative eigenvalues of $\nabla^{2}H$ and use it as a proxy for stability. We verify the stability of index-0 equilibria with additional zero eigenvalues by simulating perturbed trajectories. The specific expression for the Hessian in Equation 6 in a transformed frame is given by Equation S81 in the Supplementary Appendix, together with those for the whole energy homotopy, which will be described in subsequent subsections.

### 2.2 Mode-based approximation: A coarse description

In this subsection, we introduce a mode-based, phase-decoupled approximation that provides a coarse description of the energy landscape. The goal is to capture the main features of the stationary statistics of the system as described by Equation 1 and to obtain a convenient starting point for the subsequent, more accurate landscape exploration. For this purpose, we transform the state variable into an orthogonal eigenbasis $E=\left[e_{1}\;e_{2}\;\cdots e_{N}\right]$ obtained from the eigendecomposition of the linear part of Equation 1,

$$W+\text{Diag }\left(a\right)=E\text{Diag }\left(d\right)E^{⊤},$$

where $a={\left[a_{1}\;a_{2}\;\cdots \;a_{N}\right]}^{⊤}$ and $d={\left[d_{1}\;d_{2}\;\cdots \;d_{N}\right]}^{⊤}$ collects the corresponding eigenvalues. Let $z=E^{⊤}s$ denote the projection of the original state variable s onto the eigenbasis and introduce polar coordinates (r,θ) again for the new state variable z , by z=r◦exp(iθ) . Then, the energy function H can be rewritten in (r,θ) following from the coordinate transformations as

$$\begin{array}{cc} H\left(r,\theta \right)=\frac{1}{4}\overset{N}{\underset{i=1}{\sum }}\rho_{i}^{4}-\frac{1}{2}\overset{N}{\underset{i=1}{\sum }}a_{i}\rho_{i}^{2}-\frac{1}{2}\overset{N}{\underset{i=1}{\sum }}\overset{N}{\underset{j=1}{\sum }}w_{ij}\rho_{i}\rho_{j}\exp\left(i\left(\phi_{i}-\phi_{j}\right)\right)\\ & \overset{s=\rho ◦\exp\left(i\phi \right)}{=}\frac{1}{4}\overset{N}{\underset{i=1}{\sum }}{\left(s_{i}^{†}s_{i}\right)}^{2}-\frac{1}{2}s^{†}\text{Diag }\left(a\right)s-\frac{1}{2}s^{†}Ws\\ & \overset{s=Ez}{=}\frac{1}{4}\overset{N}{\underset{i=1}{\sum }}{\left(\overset{N}{\underset{j_{1}=1}{\sum }}\overset{N}{\underset{j_{2}=1}{\sum }}e_{ij_{1}}e_{ij_{2}}z_{j_{1}}^{†}z_{j_{2}}\right)}^{2}-\frac{1}{2}z^{†}\text{Diag }\left(d\right)z\\ & \overset{z=r◦\exp\left(i\theta \right)}{=}\frac{1}{4}\overset{N}{\underset{i=1}{\sum }}{\left[\overset{N}{\underset{j=1}{\sum }}e_{ij}^{2}r_{j}^{2}+\overset{N}{\underset{j_{1}=1}{\sum }}\underset{j_{2}\neq j_{1}}{\sum }e_{ij_{1}}e_{ij_{2}}r_{j_{1}}r_{j_{2}}\cos\left(\theta_{j_{1}}-\theta_{j_{2}}\right)\right]}^{2}-\frac{1}{2}r^{⊤}\text{Diag }\left(d\right)r, \end{array}$$ (7)

where superscript † denotes the conjugate transpose. By further defining

$$B=E◦E=E^{◦2},\;W^{\left(k\right)}=\left[w_{ij}^{\left(k\right)}\right]=\left[e_{ki}e_{kj}\right],\;C\left(\theta \right)=\left[c_{ij}\left(\theta \right)\right]=\left[\cos\left(\theta_{i}-\theta_{j}\right)-\delta_{ij}\right],$$ (8)

where ${\left(\cdot \right)}^{◦2}$ denotes elementwise squaring and $\delta_{ij}$ is the Kronecker delta, the energy can be written compactly as

$$H\left(r,\theta \right)=\frac{1}{4}\overset{N}{\underset{k=1}{\sum }}{\left[{\left(Br^{◦2}\right)}_{k}+r^{⊤}\left(W^{\left(k\right)}◦C\left(\theta \right)\right)r\right]}^{2}-\frac{1}{2}d^{⊤}r^{◦2}.$$ (9)

Because an orthogonal change of coordinates preserves the gradient-flow structure, the deterministic dynamics in (r,θ) can be obtained analogously to Equation 3 by differentiating H(r,θ) with respect to r and θ . We interpret the eigenbasis projections $z={\left[z_{1}\;z_{2}\;\cdots \;z_{N}\right]}^{⊤}$ as oscillation modes; accordingly, we refer to the gradient dynamics induced by Equation 9 as *mode dynamics* as it describes the evolution of the mode amplitudes and phases. Finally, when W+Diag (d) has repeated eigenvalues, different choices of eigenmodes in E yield different representations of the mode dynamics. We may have the following strategies to handle this ambiguity: (i) choose a basis that distributes oscillation power as evenly as possible across oscillators, for example, by Riemannian minimization of $\sum_{i,j}e_{ij}^{4}$ over the orthogonal manifold; (ii) apply random orthogonal rotations within the degenerate eigenspaces to circumvent certain singularities (as used later); or (iii) enforce the continuity of E with respect to some control parameter, implemented numerically by a small perturbation to E to lift the degeneracy.

Now, assume that the couplings are sufficiently “random” so that, for each k , the matrix $W^{\left(k\right)}$ has balanced positive/negative entries, and the phase-dependent cross term, $r^{⊤}\left(W^{\left(k\right)}◦C\left(\theta \right)\right)r$ , is negligible compared with ${\left(Br^{◦2}\right)}_{k}$ . This leads us to neglect the interaction among modes *via* the phase and approximate the energy by

$$H_{\mathrm{approx}}\left(r\right)=\frac{1}{4}{\left(Br^{◦2}\right)}^{⊤}\left(Br^{◦2}\right)-\frac{1}{2}d^{⊤}r^{◦2},$$ (10)

which further yields the *approximate* mode dynamics as an amplitude system,

$$\frac{dr}{dt}=-\frac{\partial H_{\mathrm{approx}}\left(r\right)}{\partial r}=-\left(B^{⊤}Br^{◦2}-d\right)◦r$$ (11)

with the constraints r≥0 . Here, we let ≥ and ≫ denote elementwise “no less than” and “strictly greater than,” respectively, while x>y means x≥y with x≠y . If $B^{⊤}B$ is nonsingular (hence positive definite), Equation 11 in both the deterministic and stochastic settings admits the following properties:

- *Equilibria via active sets.* Letting $\psi :=r^{◦2}$ , the critical points of $H_{\mathrm{approx}}\left(r\right)$ in Equation 10 satisfy $\left(B^{⊤}B\psi -d\right)◦\psi =0$ ; that is, each component either vanishes $\left(\psi_{i}=0\right)$ or satisfies the linear equation ${\left(B^{⊤}B\psi \right)}_{i}=d_{i}$ . The equilibria of system Equation 11 can thus be described by choosing a support, or “active set” of indices with $\psi_{i}>0$ and then solving a principal sub-system. This is expressed by
$${\left(B^{⊤}B\right)}^{\left(1,1\right)}\psi^{\left(1\right)}=d^{\left(1\right)},\;\psi^{\left(2\right)}=0,$$ (12)
  where the superscripts (1) and (2) above denote components in two complementary subspaces and ${\left(B^{⊤}B\right)}^{\left(1,1\right)}$ denotes the corresponding principal submatrix. Because every principal submatrix of a positive definite matrix is positive definite, there are at most $2^{N}$ candidate solutions (one per choice of the active set), and only those with $\psi^{\left(1\right)}\gg 0$ are feasible equilibria.
- *Uniqueness of the stable equilibrium and mode sparsity.* In terms of ψ , the approximate energy is the convex quadratic function given by Equation 13:
$$\begin{array}{cc} H_{\mathrm{approx}}^{\psi }\left(\psi \right) & =\frac{1}{4}\psi^{⊤}B^{⊤}B\psi -\frac{1}{2}d^{⊤}\psi \\ & =\frac{1}{4}{\left(\psi -{\left(B^{⊤}B\right)}^{-1}d\right)}^{⊤}B^{⊤}B\left(\psi -{\left(B^{⊤}B\right)}^{-1}d\right)-\frac{1}{4}d^{⊤}{\left(B^{⊤}B\right)}^{-1}d, \end{array}$$ (13)
  which is strongly convex on the convex set ${\left.\left[0,\infty \right.\right)}^{N}$ . Thus, it has a unique minimizer $\psi_{*}\in {\left.\left[0,\infty \right.\right)}^{N}$ , corresponding to a unique stable equilibrium $r_{*}=\psi_{*}^{◦\frac{1}{2}}$ of Equation 11; all other feasible critical points are saddles. If ${\left(B^{⊤}B\right)}^{-1}d\gg 0$ , then the minimizer $\psi_{*}={\left(B^{⊤}B\right)}^{-1}d$ (the full-support solution to Equation 12); otherwise, $\psi_{*}$ coincides with the feasible solution to Equation 12 that is nearest to ${\left(B^{⊤}B\right)}^{-1}d$ in the Mahalanobis distance induced by $B^{⊤}B$ , lying on the boundary of ${\left.\left[0,+\infty \right.\right)}^{N}$ . This suggests that, under Equation 11, some mode amplitudes tend to vanish to minimize the energy, so the stable state is typically supported on only a few oscillation modes. From the perspective of synergetics, these active modes act as *order parameters* that can organize highly coherent behavior such as synchronization.
- *Steady state under noise as a truncated Gaussian in* ψ *.* As the i.i.d. Gaussian noise in Equation 1 is invariant under orthogonal transformations, the stationary probability density in z admits a Gibbs form $P_{\text{approx}}^{\left(z\right)}\left(z\right)\propto \exp\left(-2H_{\text{approx}}^{\left(z\right)}\left(z\right)/\sigma^{2}\right)$ . Under the phase-decoupled approximation $H_{\text{approx}}^{\left(z\right)}\left(z\right)=H_{\text{approx}}\left(r\right)=H_{\text{approx}}^{\left(\psi \right)}\left(\psi \right)$ with $z◦z^{†}=r^{◦2}=\psi$ , integrating out θ and invoking $\prod_{i}r_{i}dr_{i}=2^{-N}\prod_{i}d\psi_{i}$ easily lead to $P_{\text{approx}}^{\left(\psi \right)}\left(\psi \right)\propto \exp\left(-2H_{\text{approx}}^{\left(\psi \right)}\left(\psi \right)/\sigma^{2}\right)$ , that is,
$$P_{\text{approx}}^{\left(\psi \right)}\left(\psi \right)=\frac{1}{Z\left(B,d,\sigma \right)}\exp\left[-\frac{{\left(\psi -{\left(B^{⊤}B\right)}^{-1}d\right)}^{⊤}B^{⊤}B\left(\psi -{\left(B^{⊤}B\right)}^{-1}d\right)}{2\sigma^{2}}\right]$$ (14)
  on $\psi \in {\left.\left[0,+\infty \right.\right)}^{N}$ , where Z(B,d,σ) is the normalization factor or partition function. Thus ψ follows a truncated multivariate normal distribution on the positive orthant given in Equation 15,
$$\psi ∼TN\left({\left(B^{⊤}B\right)}^{-1}d,\;\sigma^{2}{\left(B^{⊤}B\right)}^{-1};\;\psi \geq 0\right),$$ (15)
  with the previously mentioned $\psi_{*}$ attaining the maximum density. We expect this distribution to concentrate near $\psi_{*}$ as σ→0 , revealing clear dominant modes. For sufficiently large σ , fluctuations may overwhelm the deterministic dynamics and degrade the organized behavior.

Although the approximate mode dynamics (Equation 11) admits the characterization above, the associated partition function Z(B,d,σ) for the truncated normal distribution shown by Equation 14 generally has no closed-form expression. Closed-form evaluation of Z(B,d,σ) and marginal density functions is possible only for special choices of the quadratic form. In our previous work (Li et al., 2022), an even simpler mean-field approximation has been introduced by taking all entries of $B=\left[e_{ij}^{2}\right]$ to be approximately 1/N , which yields the following:

$$H_{\text{approx}}^{\left(\psi \right)}\left(\psi \right)=\frac{1}{4N}\psi^{⊤}O\psi -\frac{1}{2}d^{⊤}\psi ,$$ (16)

$$P_{\text{approx}}^{\left(\psi \right)}\left(\psi \right)=\frac{1}{Z\left(d,N,\sigma \right)}\exp\left[-\frac{1}{2N\sigma^{2}}\psi^{⊤}O\psi +\frac{1}{\sigma^{2}}d^{⊤}\psi \right],$$ (17)

for $\psi \in {\left.\left[0,+\infty \right.\right)}^{N}$ , where O is the all-one matrix. The amplitude dynamics induced by Equation 16 for r (i.e., replacing ψ by $r^{◦2}$ and then differentiating with respect to r ) reduces to

$$\frac{dr}{dt}=-\left(\frac{r^{⊤}r}{N}1-d\right)◦r.$$ (18)

When $d_{1}>d_{2}>\cdots >d_{N_{+}}>0\geq d_{N_{+}+1}\geq \cdots \geq d_{N}$ , this system has exactly $N_{+}$ nontrivial equilibria, each supported on a single mode (all other components vanish). When $d_{1}>0$ , all equilibria are saddles except $r_{*}={\left[\sqrt{Nd_{1}}\;0\;\cdots \;0\right]}^{⊤}$ , which is the unique stable equilibrium of the system given by Equation 18 and corresponds to the global minimizer of $H_{\text{approx}}^{\left(\psi \right)}\left(\psi \right)$ in Equation 16; this reflects an extreme form of mode sparsity, where only one order parameter survives, and corresponds to synchronization on the pattern encoded by the leading eigenvector $e_{1}$ . Accordingly, analytic examination of marginal densities of $P_{\text{approx}}^{\left(\psi \right)}\left(\psi \right)$ in Equation 17 shows that a ring-like distribution can arise only in the leading mode coordinate $z_{1}$ . Furthermore, this ring-like distribution collapses to a unimodal distribution when the leading eigenvalue $d_{1}$ is below a threshold $d_{th}\left(d_{2},d_{3},\ldots ,d_{N};N,\sigma \right)$ , which provides an approximate criterion for a stochastic analogue of *oscillation death* (see Section 3.8 and Li et al. (2022) for details).

### 2.3 Homotopy continuation: exploring the full landscape

The approximate analysis above provides a coarse picture of the Stuart–Landau oscillator network, highlighting the statistical dominance of a single organized oscillatory macrostate and its degradation under strong noise. However, the full energy function, Equation 4 or Equation 9, and its associated gradient dynamics may exhibit a richer critical-point structure, including multiple local minima and saddles that can mediate synergetic transitions. Resolving the full landscape typically requires numerical tools, and we use *homotopy continuation* in this work. Consistent with the preceding formulation, we work with H(r,θ) in Equation 9 and introduce the parametric homotopy as Equation 19

$$H\left(r,\theta ;\tau \right)=\frac{1}{4}\overset{N}{\underset{k=1}{\sum }}{\left[{\left(Br^{◦2}\right)}_{k}+\tau r^{⊤}\left(W^{\left(k\right)}◦C\left(\theta \right)\right)r\right]}^{2}-\frac{1}{2}d^{⊤}r^{◦2},$$ (19)

where the homotopy parameter τ∈R continuously deforms the landscape from the approximation (τ=0) to the full model (τ=1) . Starting from a critical point $\left(r_{0},\theta_{0}\right)$ of H(r,θ;0) , we attempt to trace the corresponding family of critical points as τ varies; that is, we compute a continuation curve (r(τ),θ(τ),τ) in the augmented state space, which satisfies the *equilibrium conditions*

$$\frac{\partial H\left(r,\theta ;\tau \right)}{\partial r}=0,$$ (20a)

$$\frac{\partial H\left(r,\theta ;\tau \right)}{\tau r\partial \theta }=0.$$ (20b)

An important point here is that the equilibrium condition for the phase θ would normally be ∂H/(r∂θ)=0 . However, the approximate energy landscape $H\left(r,\theta ;0\right)=H_{\mathrm{approx}}\left(r\right)$ is independent of θ so that ∂H/(r∂θ)=0 holds trivially at τ=0 and imposes no constraint on $\theta_{0}$ . This violates the nonsingularity condition required by the implicit function theorem for local continuation. We therefore impose the phase equilibrium condition up to the first order in τ using the rescaled form of Equation 20b, which remains equivalent to the original condition for τ≠0 while imposing a critical constraint at τ=0 . For continuation, we differentiate the equilibrium conditions (Equations 20a, b) with respect to τ and set the total derivatives to 0. This yields the *tangency conditions*

$$\frac{d}{d\tau }\frac{\partial H\left(r,\theta ;\tau \right)}{\partial r}=\frac{\partial^{2}H}{\partial r^{2}}\frac{dr}{d\tau }+\frac{\partial^{2}H}{\partial \theta \partial r}\frac{d\theta }{d\tau }+\frac{\partial^{2}H}{\partial \tau \partial r}=0,$$ (21a)

$$\frac{d}{d\tau }\frac{\partial H\left(r,\theta ;\tau \right)}{\tau r\partial \theta }=\left(\frac{\partial }{\partial r}\frac{\partial H}{\tau r\partial \theta }\right)\frac{dr}{d\tau }+\left(\frac{\partial }{\partial \theta }\frac{\partial H}{\tau r\partial \theta }\right)\frac{d\theta }{d\tau }+\frac{\partial }{\partial \tau }\frac{\partial H}{\tau r\partial \theta }=0,$$ (21b)

where we write H in place of H(r,θ;τ) for brevity. Equations 21a,b form an implicit ordinary differential equation for (r(τ),θ(τ)) along the solution manifold; it is also referred to as the *homotopy system*. Numerically integrating the homotopy system traces the desired continuation curves, allowing us to transport critical points from τ=0 to τ=1 and thereby compare the approximate and full energy landscapes.

Next, we briefly introduce the implementation of continuation. To handle possible fold bifurcations on the continuation curve where ∥dr/dτ∥ or ∥dθ/dτ∥ becomes infinite, we adopt an arc-length l -parametrization, instead of the above τ -parametrization, by multiplying Equation 21a and Equation 21b with dτ/dl . The homotopy system is then expressed as

$$\left[\begin{array}{ccc} \frac{\partial^{2}H}{\partial r^{2}} & \frac{\partial^{2}H}{\partial \theta \partial r} & \frac{\partial^{2}H}{\partial \tau \partial r}\\ \frac{\partial }{\partial r}\frac{\partial H}{\tau r\partial \theta } & \frac{\partial }{\partial \theta }\frac{\partial H}{\tau r\partial \theta } & \frac{\partial }{\partial \tau }\frac{\partial H}{\tau r\partial \theta } \end{array}\right]\left[\begin{array}{c} r^{\prime }\\ \theta^{\prime }\\ \tau^{\prime } \end{array}\right]=0,$$ (22)

where $\left(r^{\prime },\theta^{\prime },\tau^{\prime }\right)=\left(dr/dl,d\theta /dl,d\tau /dl\right)$ , or more briefly,

$$\left[\begin{array}{ccc} A_{11}\left(r,\theta ;\tau \right) & A_{12}\left(r,\theta ;\tau \right) & b_{1}\left(r,\theta ;\tau \right)\\ A_{21}\left(r,\theta ;\tau \right) & A_{22}\left(r,\theta ;\tau \right) & b_{2}\left(r,\theta ;\tau \right) \end{array}\right]\left[\begin{array}{c} r^{\prime }\\ \theta^{\prime }\\ \tau^{\prime } \end{array}\right]=0,$$ (23)

where $A=\left[A_{ij}\right]$ (i,j=1,2) and $b={\left[\;b_{1}^{⊤}\;b_{2}^{⊤}\;\right]}^{⊤}$ denote the corresponding blocks of derivatives in the augmented coefficient matrix in Equation 22. Moreover, this 2N×(2N+1) system only determines the tangential direction of the continuation curve, where we can apply the normalization

$${\left\|r^{\prime }\right\|}^{2}+{\left\|\theta^{\prime }\right\|}^{2}+|\tau^{\prime }{|}^{2}=1$$ (24)

to specify a unit tangential vector, with its orientation chosen by

$$\text{sgn det }\left[\begin{array}{ccc} A_{11} & A_{12} & b_{1}\\ A_{21} & A_{22} & b_{2}\\ r^{\prime ⊤} & \theta^{\prime ⊤} & \tau^{\prime } \end{array}\right]=+1\text{ or }-1.$$ (25)

Equations 23, 24 are solved and then integrated using the conventional approach of Euler’s predictor and generalized Newton’s corrector, with the algorithm details given in Section 2 of Chen and Li (2015). Generally, both orientations in Equation 25 should be attempted when continuing to τ=1 . Here, however, for each initial configuration $\left(r_{0},\theta_{0},0\right)$ with $\tau^{\prime }\neq 0$ , we retain only the orientation for which $\tau^{\prime }>0$ because any continuation curve that starts with $\tau^{\prime }<0$ and reaches τ=1 must bend back and cross τ=0 at another “initial” configuration $\left(r_{0},\theta_{0},0\right)$ . In addition, note that the continuation curves in this study should come in pairs due to the reflection symmetry (r,θ)→(r,−θ) , which entails accompanying sign reversals of $A_{12}$ , $A_{21}$ , $b_{2}$ , and $\theta^{\prime }$ for Equations 23–25 to remain satisfied.

After introducing the homotopy continuation method, we examine the specific form of our energy homotopy and its derivatives. For convenience, we expand H(r,θ;τ) in the homotopy parameter τ as Equation 26:

$$H\left(r,\theta ;\tau \right)=H_{0}\left(r\right)+\tau H_{1}\left(r,\theta \right)+\tau^{2}H_{2}\left(r,\theta \right),$$ (26)

$$H_{0}\left(r\right)=\frac{1}{4}{\left(Br^{◦2}\right)}^{⊤}\left(Br^{◦2}\right)-\frac{1}{2}d^{⊤}r^{◦2}=H_{\text{approx}}\left(r\right),$$ (27a)

$$H_{1}\left(r,\theta \right)=\frac{1}{2}\overset{N}{\underset{k=1}{\sum }}{\left(Br^{◦2}\right)}_{k}\left[r^{⊤}\left(W^{\left(k\right)}◦C\left(\theta \right)\right)r\right]=\frac{1}{2}r^{⊤}\left[V\left(r\right)◦C\left(\theta \right)\right]r,$$ (27b)

$$H_{2}\left(r,\theta \right)=\frac{1}{4}\overset{N}{\underset{k=1}{\sum }}{\left[r^{⊤}\left(W^{\left(k\right)}◦C\left(\theta \right)\right)r\right]}^{2},$$ (27c)

where $H_{1}\left(r,\theta \right)$ in Equation 27b is written compactly using V(r) , which is defined as

$$V\left(r\right)=\overset{N}{\underset{k=1}{\sum }}{\left(Br^{◦2}\right)}_{k}W^{\left(k\right)}=E^{⊤}\text{Diag }\left(Br^{◦2}\right)E.$$ (28)

This expansion clarifies appropriate initialization as τ→0 . The initial amplitudes $r_{0}$ at τ=0 are chosen as the critical points of $H_{\text{approx}}\left(r\right)$ , which have already been prepared in Section 2.2. The phase initialization is then determined at the first order by $H_{1}\left(r,\theta \right)$ because the phase equilibrium condition (Equation 20b) yields Equation 29:

$$\frac{\partial H\left(r,\theta ;\tau \right)}{\tau r\partial \theta }=\frac{\partial }{\tau r\partial \theta }\left(\tau H_{1}\left(r,\theta \right)+\tau^{2}H_{2}\left(r,\theta \right)\right)=\frac{\partial H_{1}\left(r,\theta \right)}{r\partial \theta }+O\left(\tau \right).$$ (29)

Note that, the function $H_{1}\left(r_{0},\theta \right)$ in θ , for a fixed $r_{0}$ , has exactly the form of an *XY Hamiltonian*, which is extensively studied in statistical physics. We therefore obtain $\theta_{0}$ according to Equation 30:

$$\frac{\partial H_{XY}\left(\theta \right)}{r_{0}\partial \theta }=0,\text{ with }H_{XY}\left(\theta \right):=\frac{1}{2}r_{0}^{⊤}\left[V\left(r_{0}\right)◦C\left(\theta \right)\right]r_{0}.$$ (30)

In this study, we do not seek to enumerate all critical points; instead, for each chosen $r_{0}$ of interest, we sample random phases θ , apply the generalized Newton corrector within the θ -subspace to obtain possibly multiple candidate $\theta_{0}$ , and then perform homotopy continuation up to τ=1 .

Finally, we provide full expressions for the homotopy system (Equation 23) for completeness. Although the separation of functions in r and θ in Equations 27a–c facilitates our physical understanding of the energy homotopy, the coefficient matrix blocks in the homotopy system have far more complex expressions. Nevertheless, we find that by introducing the quantities defined in Equation 31,

$$\begin{array}{cc} R & =\text{Diag }\left(r\right),\\ S\left(\theta \right) & =\left[s_{ij}\left(\theta \right)\right]=\left[\sin\left(\theta_{i}-\theta_{j}\right)\right],\\ G\left(r,\theta \right) & =E◦\left(ERC\left(\theta \right)\right),\\ F\left(r,\theta \right) & =E◦\left(ER\;S\left(\theta \right)\right), \end{array}$$ (31)

along with previously defined C(θ) in Equation 8 and V(r) in Equation 28, H(r,θ;τ) can be rewritten simply in a “quadratic” form as Equation 32 (see also Equation S68 in the Supplementary Appendix):

$$H\left(r,\theta ;\tau \right)=\frac{1}{4}r^{⊤}{\left(BR+\tau G\right)}^{⊤}\left(BR+\tau G\right)r-\frac{1}{2}r^{⊤}\text{Diag }\left(d\right)r.$$ (32)

Accordingly, the coefficient matrix blocks in Equation 23 are given by

$$A\left(r,\theta ,\tau \right)=A^{\left(0\right)}\left(r,\theta \right)+\tau A^{\left(1\right)}\left(r,\theta \right)+\tau^{2}A^{\left(2\right)}\left(r,\theta \right),$$ (33a)

$$b\left(r,\theta ,\tau \right)=b^{\left(0\right)}\left(r,\theta \right)+\tau b^{\left(1\right)}\left(r,\theta \right)=\left[\begin{array}{c} G^{⊤}BR+{\left(BR\right)}^{⊤}G\\ F^{⊤}G \end{array}\right]r+\tau \left[\begin{array}{c} 2G^{⊤}Gr\\ 0 \end{array}\right]r,$$ (33b)

where we have

$$A^{\left(0\right)}=\left[\begin{array}{cc} \text{Diag }\left(B^{⊤}Br^{◦2}-d\right)+2RB^{⊤}BR\; & 0\;\\ -V◦S+2F^{⊤}BR\; & \left(V◦C\right)R-\text{Diag }\left(\left(V◦C\right)r\right) \end{array}\right],$$ (34a)

$$\begin{array}{ll} & A^{\left(1\right)}=\\ & \left[\begin{array}{cc} V◦C+Diag\left(B^{⊤}Gr\right)+2G^{⊤}BR+2{\left(BR\right)}^{⊤}G & \left(V◦S\right)R+2{\left(BR\right)}^{⊤}FR-\text{Diag }\left(\left(V◦S\right)r\right)\\ -S◦\left(E^{⊤}\text{Diag }\left(Gr\right)E\right)+2F^{⊤}G & \left[C◦\left(E^{⊤}\text{Diag }\left(Gr\right)E\right)\right]R+2F^{⊤}FR-\text{Diag }\left(G^{⊤}Gr\right) \end{array}\right], \end{array}$$ (34b)

$$\begin{array}{ll} & A^{\left(2\right)}=\\ & \left[\begin{array}{cc} C◦\left(E^{⊤}\text{Diag }\left(Gr\right)E\right)+2G^{⊤}G\; & \left[S◦\left(E^{⊤}\text{Diag }\left(Gr\right)E\right)\right]R+2G^{⊤}FR+\text{Diag }\left(F^{⊤}Gr\right)\\ 0\; & 0 \end{array}\right]. \end{array}$$ (34c)

In Equations 33a,b, 34a–c, the explicit dependence of the relevant quantities on r and θ has been suppressed for compactness of notation. We refer the reader to the Supplementary Appendix for detailed derivations of these expressions, together with the necessary preliminaries provided therein.

### 2.4 Additional singularity at initialization: multiple branches

In the previous subsection, a first-order phase equilibrium condition is introduced for the specific form of homotopy (Equation 19) so that the initial phases can be determined from the critical points of an XY Hamiltonian and numerically computed using, for example, the Newton–Raphson method. Following from the implicit function theorem, isolated initial phase configurations can be obtained when the corresponding Jacobian block, given by Equation 35,

$$A_{22}^{\left(0\right)}\left(r_{0},\theta_{0}\right)=\left(\frac{\partial }{\partial \theta }\frac{\partial H_{XY}\left(\theta \right)}{r_{0}\partial \theta }\right){|}_{\theta =\theta_{0}}=\left[\left(V◦C\right)R-\text{Diag }\left(\left(V◦C\right)r\right)\right]{|}_{\begin{array}{c} r=r_{0}\\ \theta =\theta_{0} \end{array}},$$ (35)

has rank N−1 , after excluding the degree of freedom associated with the global phase shift. However, this full-rank condition does not necessarily hold, especially in the presence of additional symmetries. When $A_{22}^{\left(0\right)}\left(r_{0},\theta_{0}\right)$ is singular, the first-order phase equilibrium condition alone is insufficient to determine isolated phase equilibria because flat directions may remain along which the energy gradient varies only at order $O\left(\tau^{2}\right)$ . In such cases, the $O\left(\tau^{2}\right)$ term in homotopy Equation 26 must be taken into account to identify the phase configurations that persist as isolated continuation branches. This motivates the second-order phase equilibrium condition introduced below. In this subsection, we consider the singular cases where $\partial H_{XY}/\left(r_{0}\partial \theta \right)\equiv 0$ on a manifold of dimension greater than one; this implies that the matrix $A_{22}^{\left(0\right)}\left(r_{0},\theta \right)$ has a locally constant rank strictly less than N−1 along this manifold. Here, we assume that the Jacobian block for r at $\left(r,\tau \right)=\left(r_{0},0\right)$ , that is, $A_{11}^{\left(0\right)}\left(r_{0}\right)$ , is nonsingular.

#### 2.4.1 Strong singularity

We first consider the extreme case in which the XY Hamiltonian, $H_{XY}\left(\theta \right)=\frac{1}{2}r_{0}^{⊤}\left(V\left(r_{0}\right)◦C\left(\theta \right)\right)r_{0}$ , is independent of θ . Equivalently, the phase equilibrium condition satisfies $\partial H_{XY}\left(\theta \right)/\left(r_{0}\partial \theta \right)=-\left(V\left(r_{0}\right)◦S\left(\theta \right)\right)r_{0}\equiv 0$ for all θ , so the Jacobian block $A_{22}^{\left(0\right)}\left(r_{0},\theta \right)$ vanishes identically. In this strongly singular case, the initial phase $\theta_{0}$ cannot be determined by the preceding equilibrium condition, and the continuation with Equation 23 cannot be conducted in the usual way. To de-trivialize continuation at τ=0 , we further rescale the phase equilibrium condition (Equation 20b) and impose the second-order form $\partial H\left(r,\theta ;\tau \right)/\left(\tau^{2}r\partial \theta \right)=0$ . Taking the limit τ→0 yields

$$\begin{array}{cc} \underset{\tau \to 0}{\text{lim}}\frac{\partial H}{\tau^{2}r\partial \theta } & =\underset{\tau \to 0}{\text{lim}}\left(\frac{\partial H_{1}}{\tau r\partial \theta }+\frac{\partial H_{2}}{r\partial \theta }\right)=\left(\frac{d}{d\tau }\frac{\partial H_{1}}{r\partial \theta }+\frac{\partial H_{2}}{r\partial \theta }\right){|}_{\tau =0}\\ & =\left[\left(\frac{\partial }{\partial r}\frac{\partial H_{1}}{r\partial \theta }\right)\frac{dr}{d\tau }+\left(\frac{\partial }{\partial \theta }\frac{\partial H_{1}}{r\partial \theta }\right)\frac{d\theta }{d\tau }+\frac{\partial }{\partial \tau }\frac{\tau \partial H_{2}}{r\partial \theta }\right]{|}_{\tau =0}\\ & =\left[\left(\frac{\partial }{\partial r}\frac{\partial H}{\tau r\partial \theta }\right)\frac{dr}{d\tau }+\left(\frac{\partial }{\partial \theta }\frac{\partial H}{\tau r\partial \theta }\right)\frac{d\theta }{d\tau }+\frac{\partial }{\partial \tau }\frac{\partial H}{\tau r\partial \theta }\right]{|}_{\tau =0}=0, \end{array}$$ (36)

where we have used $\left(\partial H_{1}\left(r_{0},\theta \right)/\left(r_{0}\partial \theta \right)\right){|}_{\theta =\theta_{0}}=0$ for the second equality. Equation 36 shows that the phase equilibrium condition at τ=0 in the singular case coincides with the phase tangency condition (Equation 21b). In the strongly singular setting, this leads to

$$\left[\begin{array}{c} A_{11}^{\left(0\right)}\left(r_{0}\right)\;\\ A_{21}^{\left(0\right)}\left(r_{0},\theta \right) \end{array}\right]\frac{dr}{d\tau }+\left[\begin{array}{c} b_{1}^{\left(0\right)}\left(r_{0},\theta \right)\\ b_{2}^{\left(0\right)}\left(r_{0},\theta \right) \end{array}\right]=0,$$ (37)

where θ and dr/dτ are unknowns whose solutions yield the initial phase $\theta_{0}$ and the initial tangent $\left(dr/d\tau \right){|}_{\tau =0}$ . This system is linear in dr/dτ , while θ enters through the coefficients in a nonlinear fashion. Because $A_{11}^{\left(0\right)}\left(r_{0}\right)$ is invertible, we can eliminate dr/dτ and obtain the residual (suppressing the explicit dependence on $r_{0}$ for brevity):

$$f_{s}\left(\theta \right):=b_{2}^{\left(0\right)}\left(\theta \right)-A_{21}^{\left(0\right)}\left(\theta \right)A_{11}^{\left(0\right)-1}b_{1}^{\left(0\right)}\left(\theta \right).$$ (38)

After fixing the global phase-shift degree of freedom, the nonlinear system $f_{s}\left(\theta \right)=0$ comprises N−1 equations in N−1 unknowns and can be solved for possibly multiple solutions $\theta_{0}$ , using iterative methods such as Newton’s method and the Gauss–Newton method.

#### 2.4.2 Weak and general singularity

We now consider the general singular scenario in which the Jacobian block $A_{22}^{\left(0\right)}\left(r_{0},\theta \right)$ is rank-deficient, with locally constant rank at most N−2 on a manifold in the θ -subspace. This occurs when the condition expressed by Equation 30 admits additional degrees of freedom; for instance, two subgroups of oscillation modes may have internally fixed phase patterns while the relative phase difference between the groups can be arbitrary, yielding $A_{22}^{\left(0\right)}\left(r_{0},\theta \right)$ with a rank of N−2 . In this setting, the same second-order rescaling argument as Equation 36 applies, leading to the system

$$\left[\begin{array}{cc} A_{11}^{\left(0\right)}\left(r_{0}\right)\; & 0\;\\ A_{21}^{\left(0\right)}\left(r_{0},\theta \right)\; & A_{22}^{\left(0\right)}\left(r_{0},\theta \right) \end{array}\right]\left[\begin{array}{c} x\;\\ y \end{array}\right]+\left[\begin{array}{c} b_{1}^{\left(0\right)}\left(r_{0},\theta \right)\\ b_{2}^{\left(0\right)}\left(r_{0},\theta \right) \end{array}\right]=0$$ (39)

at τ=0 , where θ , x , and y are unknowns whose solutions provide $\theta_{0}$ , $\left(dr/d\tau \right){|}_{\tau =0}$ , and $\left(d\theta /d\tau \right){|}_{\tau =0}$ , respectively. Because $A_{11}^{\left(0\right)}\left(r_{0}\right)$ is invertible, we can eliminate x and write Equation 39 as

$$A_{22}^{\left(0\right)}\left(\theta \right)y+b_{2}^{\left(0\right)}\left(\theta \right)-A_{21}^{\left(0\right)}\left(\theta \right)A_{11}^{\left(0\right)-1}b_{1}^{\left(0\right)}\left(\theta \right)=0,$$ (40)

where the dependence on $r_{0}$ is suppressed for brevity. For a given θ , the above equation poses a linear solvability problem in y : letting $y_{*}\left(\theta \right)$ denote any least-squares solution of y for a given θ , we can define the residual as Equation 41,

$$f\left(\theta \right):=A_{22}^{\left(0\right)}\left(\theta \right)y_{*}\left(\theta \right)+b_{2}^{\left(0\right)}\left(\theta \right)-A_{21}^{\left(0\right)}\left(\theta \right)A_{11}^{\left(0\right)-1}b_{1}^{\left(0\right)}\left(\theta \right),$$ (41)

so that the solvability of Equation 40 for y is equivalent to the equation f(θ)=0 . Because $\theta_{0}$ must also lie on the critical manifold for phase equilibrium, we can finally formulate the initialization as the following coupled system in θ :

f(θ)=0, (42a)

$$\frac{\partial H_{XY}\left(\theta \right)}{r_{0}\partial \theta }=0.$$ (42b)

Note that, for a fixed θ , the solvability of Equation 40 for y generally imposes $N-1-\text{rank }A_{22}^{\left(0\right)}$ independent compatibility conditions (after excluding global shifts). In contrast, the local manifold given by Equation 42b itself has dimension $N-1-\text{rank }A_{22}^{\left(0\right)}$ . Thus, Equations 42a,b supplies precisely N−1 constraints to determine isolated admissible phases $\theta_{0}$ in generic situations. The strongly singular case is recovered as the special case where $A_{22}^{\left(0\right)}\left(\theta \right)\equiv 0$ so that Equation 42b becomes the full θ -subspace and f(θ) reduces to $f_{s}\left(\theta \right)$ in Equation 38. Equations 42a,b can be solved iteratively, for example, using a constrained Gauss–Newton-type method, in which each iteration computes a trial step δθ and performs a line search on it. This trial step is given by the Karush–Kuhn–Tucker (KKT) system in Equation 43:

$$\left[\begin{array}{cc} K{\left(\theta \right)}^{⊤}K\left(\theta \right) & A_{22}^{\left(0\right)}{\left(\theta \right)}^{⊤}\;\\ A_{22}^{\left(0\right)}\left(\theta \right) & 0 \end{array}\right]\left[\begin{array}{c} \delta \theta \;\\ \mu \end{array}\right]=-\left[\begin{array}{c} K{\left(\theta \right)}^{⊤}f\left(\theta \right)\;\\ \partial H_{XY}\left(\theta \right)/\left(r_{0}\partial \theta \right) \end{array}\right],$$ (43)

where K(θ) is a Jacobian proxy that satisfies $\partial {\left\|f\left(\theta \right)\right\|}^{2}/\partial \theta =2K{\left(\theta \right)}^{⊤}f\left(\theta \right)$ , and μ is an auxiliary vector returned. Further details about this method and explicit expressions for the relevant quantities are provided in the Supplementary Appendix.

#### 2.4.3 Mode reduction

When the initial amplitude $r_{0}$ has a support (an active set) S with the corresponding inactive set $S^{c}=\left\{1,2,\cdots \;,N\right\}\backslash S$ , it may happen that the modes indexed by a subset of $S^{c}$ become structurally degenerate: the corresponding columns of both $A_{22}^{\left(0\right)}\left(\theta \right)$ and K(θ) vanish identically on a local critical manifold. Hence, the associated phase components do not affect either condition in Equations 42a,b. This results in a singular KKT system and a non-isolated solution set, but this situation can be handled simply by disregarding these degenerate modes and restricting the computation to the remaining ones. In practice, such degenerate modes typically remain silent along the continuation curve, allowing initialization and tracing to be performed as usual in the reduced system. We refer to this as a *mode reduction*, in which mode sparsity persists beyond the phase-decoupled approximation and into the entire homotopy system.

Because $r_{0}$ , $\theta_{0}$ , and ${\left(dr/d\tau \right)}_{\tau =0}$ are now fixed, our remaining concern would be the determination of dθ/dτ at τ=0 . To this end, we differentiate the corresponding equilibrium condition to consider the phase tangency condition at the second order. Taking the limit τ→0 and using $\partial H_{1}/\left(r\partial \theta \right)=0$ at $\left(r,\theta \right)=\left(r_{0},\theta_{0}\right)$ , we obtain Equation 44:

$$\begin{array}{cc} \underset{\tau \to 0}{\lim}\frac{d}{d\tau }\frac{\partial H}{\tau^{2}r\partial \theta } & =\underset{\tau \to 0}{\lim}\left[\left(\frac{\partial }{\partial r}\frac{\partial H}{\tau^{2}r\partial \theta }\right)\frac{dr}{d\tau }+\left(\frac{\partial }{\partial \theta }\frac{\partial H}{\tau^{2}r\partial \theta }\right)\frac{d\theta }{d\tau }+\frac{\partial }{\partial \tau }\left(\frac{\partial H_{1}}{\tau r\partial \theta }+\frac{\partial H_{2}}{r\partial \theta }\right)\right]\\ & =\underset{\tau \to 0}{\lim}\left[\left(\frac{1}{\tau }\frac{\partial }{\partial r}\frac{\partial H_{1}}{r\partial \theta }+\frac{\partial }{\partial r}\frac{\partial H_{2}}{r\partial \theta }\right)\frac{dr}{d\tau }+\left(\frac{1}{\tau }\frac{\partial }{\partial \theta }\frac{\partial H_{1}}{r\partial \theta }+\frac{\partial }{\partial \theta }\frac{\partial H_{2}}{r\partial \theta }\right)\frac{d\theta }{d\tau }+\frac{\partial }{\partial \tau }\frac{\partial H_{1}}{\tau r\partial \theta }\right]\\ & =\left[\left(\frac{\partial }{\partial r}\frac{\partial H_{2}}{r\partial \theta }\right)\frac{dr}{d\tau }+\left(\frac{\partial }{\partial \theta }\frac{\partial H_{2}}{r\partial \theta }\right)\frac{d\theta }{d\tau }\right]{|}_{\tau =0}+\underset{\tau \to 0}{\lim}\frac{d}{d\tau }\frac{\partial H_{1}}{\tau r\partial \theta }\\ & =\left[\left(\frac{\partial }{\partial r}\frac{\partial H_{2}}{r\partial \theta }\right)\frac{dr}{d\tau }+\left(\frac{\partial }{\partial \theta }\frac{\partial H_{2}}{r\partial \theta }\right)\frac{d\theta }{d\tau }+\frac{{\;d}^{2}}{d\tau^{2}}\frac{\partial H_{1}}{r\partial \theta }\right]{|}_{\tau =0}=0, \end{array}$$ (44)

or more simply, the following equation for dθ/dτ :

$$A_{21}^{\left(1\right)}\left(r_{0},\theta_{0}\right)\frac{dr}{d\tau }{|}_{\tau =0}+A_{22}^{\left(1\right)}\left(r_{0},\theta_{0}\right)\frac{d\theta }{d\tau }+\frac{{\;d}^{2}}{d\tau^{2}}\frac{\partial H_{1}}{r\partial \theta }{|}_{\tau =0}=0.$$ (45)

Here, the first term is known, whereas the third term depends implicitly on ${\left(d\theta /d\tau \right)}_{\tau =0}$ and is algebraically cumbersome. In practice, we evaluate this term numerically (e.g., by a central difference approximation); detailed analysis of Equation 45 is beyond the scope of this article. Because tangents such as ${\left(d\theta /d\tau \right)}_{\tau =0}$ are used only in the Euler predictor at the start of continuation, moderate errors are typically corrected by the subsequent generalized Newton corrector, which projects each predicted point back onto a continuation curve near $\left(r_{0},\theta_{0},0\right)$ . Finally, because $\theta_{0}$ may not be unique, multiple admissible initial phases may give rise to multiple continuation branches emanating from the same critical amplitude $r_{0}$ of the approximate landscape $H_{0}\left(r\right)$ , some of which can be tracked to the full system at τ=1 .

## 3 Numerical results

### 3.1 Basic setting for a frustrated network

Here, we consider a network of $N=M^{2}$ coupled oscillators arranged on a 2D square lattice with the periodic boundary condition in both directions (i.e., a toroidal grid). Each oscillator interacts with its four nearest neighbors; this local coupling is encoded by a symmetric, hollow, 4-regular adjacency matrix $L\in R^{N\times N}$ . Moreover, to incorporate uniform global frustration, which represents long-range mean-field inhibition or antiferromagnetic interaction, we augment the local coupling with an all-to-all interaction term I−O , where O is the all-one matrix, and I is the identity. The total coupling matrix is given in Equation 46:

$$W=\kappa \left[\frac{1}{4}L+\frac{\kappa_{f}}{N}\left(I-O\right)\right],$$ (46)

where $\kappa ,\kappa_{f}\in R$ control the overall coupling strength and relative strength of frustration, respectively. The interplay between L and I−O introduces competition between local synchronization and global desynchronization, yielding a rich structure that shapes the collective dynamics of the network. Throughout this article, we fix the overall coupling strength at κ=0.1 and set the intrinsic oscillation magnitude $a_{i}=1$ for all i and the common intrinsic frequency b=π/50 (period 100).

The competing influences of local excitatory and global inhibitory couplings are reflected by the eigenvalue spectrum of the total coupling W . The lattice adjacency matrix L is diagonalizable in the Fourier basis and has well-known eigenvalues given by Equation 47:

$$\lambda_{j,l}\left(L\right)=2\cos\left(\frac{2\pi j}{M}\right)+2\cos\left(\frac{2\pi l}{M}\right)$$ (47)

for j,l∈{0,1,2,…,M−1} ; the largest eigenvalue is $\lambda_{0,0}\left(L\right)=4$ , with corresponding eigenvector 1 (the uniform mode). The frustration term has eigenvalues {λ(I−O)}={1−N,1,1,…,1} , where the single eigenvalue 1−N also corresponds to an eigenvector 1 . Because L and I−O commute, the eigenvalue spectrum of the total coupling is given in such a structured way as Equation 48:

$$\lambda_{j,l}\left(\kappa^{-1}W\right)=\left\{\begin{array}{cc} 1+\kappa_{f}\left(\frac{1}{N}-1\right),\; & \text{ for }\left(j,l\right)=\left(0,0\right),\\ \frac{1}{2}\left[\cos\left(\frac{2\pi j}{M}\right)+\cos\left(\frac{2\pi l}{M}\right)\right]+\frac{\kappa_{f}}{N},\; & \text{ otherwise}. \end{array}\right.$$ (48)

**FIGURE 1:**
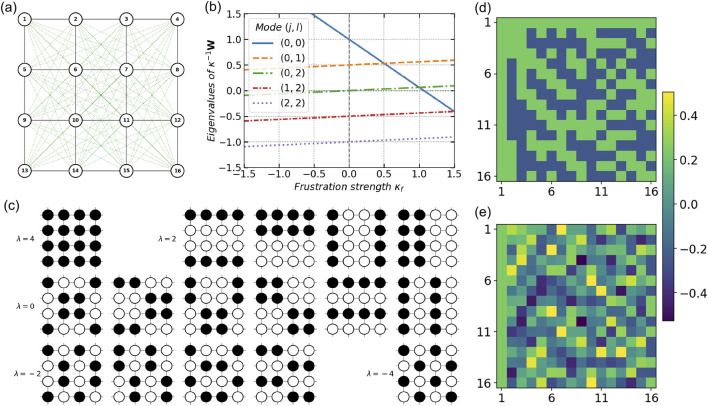
Basic network setting for the 4×4 periodic 2D lattice. **(a)** Schematic of the toroidal grid with nearest-neighbor couplings (in gray) and global frustration (in green). **(b)** Eigenvalues of the matrix $\kappa^{-1}W$ as functions of the frustration strength $\kappa_{f}$ , with the curves labeled by their corresponding Fourier modes (j,l) . For $\kappa_{f}<0.5$ , the distinct eigenvalues (from largest to smallest) have multiplicities of 1 (mode (0,0)), 4 (modes (0,1), (1,0), (0,3), and (3,0)), 6 (modes (0,2) , (2,0) , (1,1), (1,3), (3,1) , and (3,3) ), 4 (modes (1,2), (2,1), (2,3), and (3,2)), and 1 (mode (2,2)). **(c)** 2D Hadamard patterns on the 4×4 torus, grouped and labeled by the corresponding eigenvalues of L . **(d)** Normalized Hadamard matrix $E_{H}$ and **(e)** a randomly selected basis E obtained by rotations within eigenspaces corresponding to repeated eigenvalues. The column ordering matches **(c)**, and the oscillator indices follow **(a)**; this representative E is used to construct the approximate equilibria $r_{0}$ involved in Figures 2–7.

In other words, as $\kappa_{f}$ increases, the ground mode is penalized by the global frustration, leading to a decreasing eigenvalue, while all the other eigenvalues are shifted upward uniformly. We consider $N=4^{2}$ in this article, with a schematic of the lattice structure shown in Figure 1a and the change in eigenvalues with $\kappa_{f}$ shown in Figure 1b.

For $N=4^{2}$ , it is easily verified that a common orthonormal basis that simultaneously diagonalizes L and I−O (hence W , for any $\kappa_{f}$ ) can be chosen as the normalized Hadamard matrix, whose entries all have an absolute value 1/4 . The Hadamard basis, denoted by $E_{H}$ , corresponds to modes for which the oscillation power is evenly distributed across oscillators. It can be obtained by Riemannian minimization of $\sum_{i,j}e_{ij}^{4}$ over the orthogonal manifold. Figure 1c showcases the 2D patterns of these oscillation modes, corresponding to reshaped column vectors of $E_{H}$ , which is shown in Figure 1d. Finally, a general orthonormal basis E , obtained by random rotations of $E_{H}$ within the eigenspaces corresponding to repeated eigenvalues, is shown in Figure 1e; this representative orthonormal basis E , and other randomly sampled ones, will be used to isolate the initial amplitudes $r_{0}$ , as discussed later.

### 3.2 Approximate equilibria ( $H_{0}$ critical points)

Next, we investigate the coupled oscillator lattice starting from the equilibria $r_{0}$ to Equation 11, which are used to initialize the continuation of critical points of the homotopy (Equation 19) at τ=0 . With the Hadamard basis $E_{H}$ , both matrices $B_{H}\left(=E_{H}◦E_{H}\right)$ and $B_{H}^{⊤}B_{H}$ reduce to $N^{-1}O$ . This coincides with the naïve mean-field model in Li et al. (2022), where Equation 18 admits exactly $N_{+}$ nontrivial equilibria when W+Diag (a) has exactly $N_{+}$ distinct positive eigenvalues, and Equation 17 describes the stationary distribution even when the eigenvalues are repeated. However, the drawback of this singular structure in our setting is that the active-set system ${\left(B_{H}^{⊤}B_{H}\right)}^{\left(1,1\right)}r^{\left(1\right)◦2}=d^{\left(1\right)}$ in Equation 12 typically has no isolated solutions for critical points $r_{0}$ of $H_{0}\left(r\right)$ , except in the eigenspace of a single eigenvalue. This renders the Jacobian block $A_{11}^{\left(0\right)}\left(r_{0}\right)$ in Equation 39 singular, violating the fundamental requirement for continuation. To avoid such a degeneracy, we construct $H_{0}\left(r\right)$ using the randomly generated orthonormal basis E shown in Figure 1e; with probability one, $B^{⊤}B$ then has 32,003 nonsingular principal submatrices, while H(r,θ) in Equation 9 remains invariant.

**FIGURE 2:**
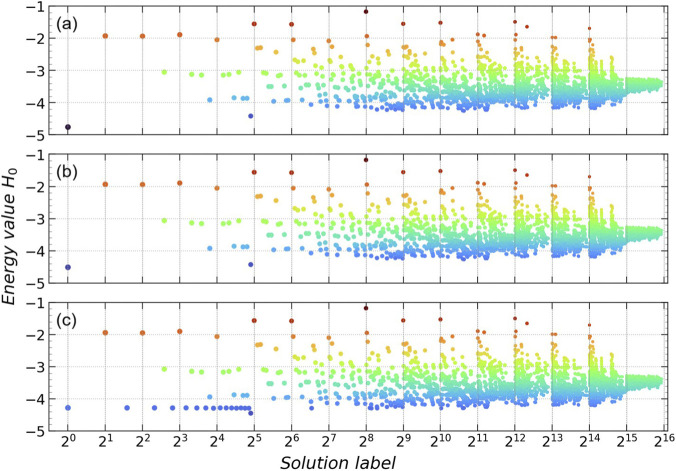
Energy values $H_{0}\left(r_{0}\right)$ of the approximate equilibria $r_{0}$ obtained from Equation 12 with the random orthonormal basis E in Figure 1e. Panels show **(a)** $\kappa_{f}=0.1$ , **(b)** $\kappa_{f}=0.4$ , and **(c)** $\kappa_{f}=0.7$ . The horizontal axis is the integer solution label, shown on a $\log_{2}$ scale; its base-2 representation encodes the support (active set) of $r_{0}$ in a least-significant-bit-first (LSB-first) convention, with the rightmost bit corresponding to the first vector component. Each dot corresponds to one feasible solution, and the trivial solution #0 is omitted. For large frustration $\left(\kappa_{f}=0.7\right)$ , solution #1 attains a higher $H_{0}$ than solution #30, and additional solutions, including #3, #5, #7, …  , #29, also appear.

We calculated the solutions $r_{0}$ to Equation 12 and plotted the corresponding energy values $H_{0}\left(r_{0}\right)$ in Figure 2, for $\kappa_{f}=0.1$ , 0.4, and 0.7. Each solution is labeled with an integer in $\left\{0,1,2,\ldots ,2^{16}-1\right\}$ , whose base-2 representation specifies the active set (i.e., the indices of nonzero components of $r_{0}$ ), with the rightmost bit corresponding to the leading vector component. For example, a solution of the form ${\left[0\;r_{2}\;r_{3}\;r_{4}\;r_{5}\;0\;\cdots \;0\right]}^{⊤}$ with $r_{2},r_{3},r_{4},r_{5}>0$ is labeled by ${\left(11110\right)}_{2}$ , that is, 30. Under the current parameter setting, a random basis E typically yields roughly $\left(3.3\pm 0.6\right)\times 10^{3}$ feasible solutions $r_{0}$ ; in our calculation, the numbers of solutions are about $4.3\times 10^{3}$ . Although varying $\kappa_{f}$ changes the range of eigenvalues in d , Figure 2 shows that the overall energy patterns of the approximate equilibria remain broadly similar. Among solutions with labels in $\left.\left[2^{n},2^{n+1}\right.\right)$ (1≤n≤14) , solution # $2^{n}$ typically has a particularly high $H_{0}$ . Meanwhile, solutions that are close to “all-one” (up to scaling) within the selected eigenspace tend to achieve lower $H_{0}$ , especially the small-numbered ones with nonzero leading modes (e.g., solutions #1 and #30).

Varying the frustration strength $\kappa_{f}$ modifies the details of the energy pattern in Figure 2. A notable change is the relative ordering of solutions #1 and #30: solution #1 has the lowest $H_{0}$ when $\kappa_{f}<0.5$ , while the energy gap between them decreases as $\kappa_{f}$ increases. Once $\kappa_{f}>0.5$ , solution #30 attains a lower $H_{0}$ than solution #1, and a series of odd-labeled solutions emerge (e.g., #3, #5, … , #29, #93, … , #1551). These solutions are accompaniments to solution #1, taking the form ${\left[r_{1}\;\varepsilon_{2}\;\varepsilon_{3}\;\cdots \;\varepsilon_{11}\;0\;\cdots \;0\right]}^{⊤}$ with $0\leq \varepsilon_{2},\varepsilon_{3},\ldots ,\varepsilon_{11}\ll r_{1}$ . This trend aligns with the intuition that the preferred configuration of the coupled oscillators shifts from full to partial synchronization, as global frustration increases.

### 3.3 Homotopy continuation instances

Now, we perform homotopy continuation from selected solutions $r_{0}$ and examine how the equilibria of $H_{0}\left(r\right)$ (i.e., H(r,θ;0) ) continue to those of H(r,θ;1) . We focus mainly on solutions #1 and #30, which attain the lowest and second-lowest values of $H_{0}$ , and also consider solutions #28, #24, and #32 as representative higher-energy examples. For $\kappa_{f}=0.7$ , we additionally investigate the small-odd-numbered solutions accompanying solution #1, for example, solution #25, which turns out to be weakly singular.

#### 3.3.1 Solution #1

This is a highly degenerate case exhibiting strong singularity and mode reduction. At τ=0 , for any θ , all blocks in the coefficient matrix in Equation 22 vanish except $A_{11}^{\left(0\right)}\left(r_{0}\right)$ . In particular, condition Equation 37 implies $dr/d\tau {|}_{\tau =0}=0$ , while $\theta_{0}$ remains arbitrary. Moreover, the above-mentioned vanishing blocks remain zero for finite τ . Consequently, the continuation reduces to dr/dτ≡0 , and the homotopy system effectively collapses to the first mode, with equilibrium #1 persisting for all τ∈[0,1] . Numerical calculation shows that this equilibrium maintains an index of 0 (i.e., no negative eigenvalues) throughout τ∈[0,1] when $\kappa_{f}=0.1$ and 0.4; for $\kappa_{f}=0.7$ , the index is initially 8, decreases to 4 at a small τ , and then remains 4 up to τ=1 . The energy values are H=−4.758 , −4.516 , and −4.280 for $\kappa_{f}=0.1$ , 0.4, and 0.7, respectively.

#### 3.3.2 Solution #30

Similar to solution #1, solution #30 at τ=0 remains invariant for any choice of orthonormal basis E . It is also a strongly singular case in the sense that $A_{22}^{\left(0\right)}\left(r_{0},\theta \right)\equiv 0$ for any θ . On the other hand, the choice of E affects the admissible initial conditions that satisfy Equations 37, 45. With the basis E in Figure 1e and $\kappa_{f}=0.4$ , we could find 50 initial phase configurations: 8 with collinear phases and 21 non-collinear configurations together with their sign-flipped counterparts (arising from the reflection symmetry). Here, we fix $\theta_{2}=0$ to remove the global phase-shift degree of freedom. In all successful runs, the homotopy system reduces to at most eight modes, with a potential support {2,3,4,5,12,13,14,15} ; the continuation curves that reach τ=1 can be grouped into the following five types:

1. The homotopy system is reduced to four modes $\left(r_{2},r_{3},r_{4},r_{5}\neq 0\right)$ ; the coefficient matrix along the continued curve has rank 19 ( $A_{22}^{\left(0\right)}$ has rank 3) in a neighborhood of τ=0 ;
2. An 8-mode reduction, with non-collinear initial phases and vanishing $r_{12}$ , $r_{13}$ , $r_{14}$ , and $r_{15}$ at τ=1 ;
3. An 8-mode reduction, with collinear initial phases and vanishing $r_{12}$ , $r_{13}$ , $r_{14}$ , and $r_{15}$ at τ=1 ;
4. An 8-mode reduction, with non-collinear initial phases and non-vanishing $r_{12}$ , $r_{13}$ , $r_{14}$ , and $r_{15}$ at τ=1 ;
5. An 8-mode reduction, with collinear initial phases and non-vanishing $r_{12}$ , $r_{13}$ , $r_{14}$ , and $r_{15}$ at τ=1 .

Here, by “collinear,” we mean that the relevant components of θ are {0,π} -locked. For cases (ii)–(v), the coefficient matrix along the continued curves has rank 23 ( $A_{22}^{\left(0\right)}$ has rank 7) in a neighborhood of τ=0 .

**FIGURE 3:**
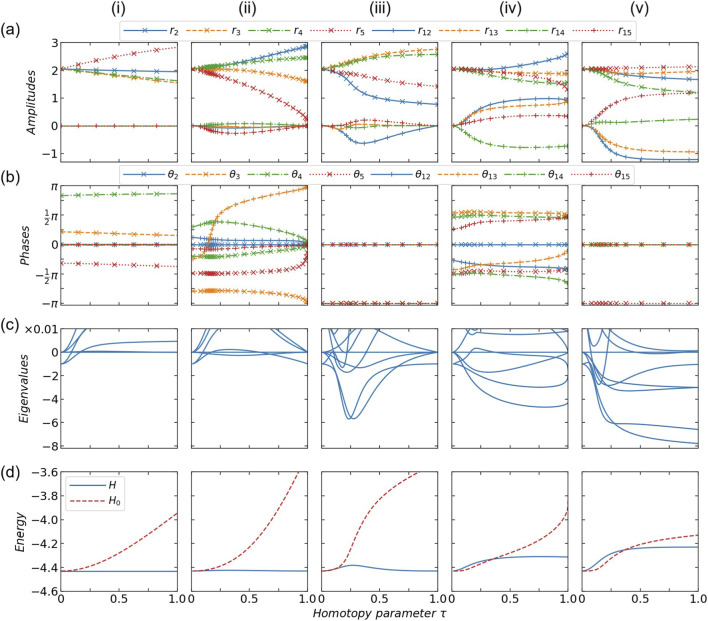
Representative continuation results initialized from solution #30 with $\kappa_{f}=0.4$ , for types (i)–(v). As functions of τ∈[0,1] , we display **(a)** the mode amplitudes r , **(b)** the phases θ , **(c)** the negative eigenvalues of the equilibrium, and **(d)** the energy H(r,θ;τ) , together with $H_{0}\left(r\right)$ for visualizing the discrepancies caused by the θ -dependent cross term in Equation 19. In case (i), the homotopy system reduces to four modes and yields an index-0 equilibrium, whereas in the other cases, it reduces to eight modes and leads to saddles with indices between 1 and 5. The equilibria at τ=1 in (i), (ii), and (iii) share the same energy H=−4.431 , which also equals the initial energy at τ=0 . Furthermore, note that the additional zero eigenvalues at τ=1 (other than the enduring one due to the global phase-shift invariance) imply degeneracy.

**FIGURE 4:**
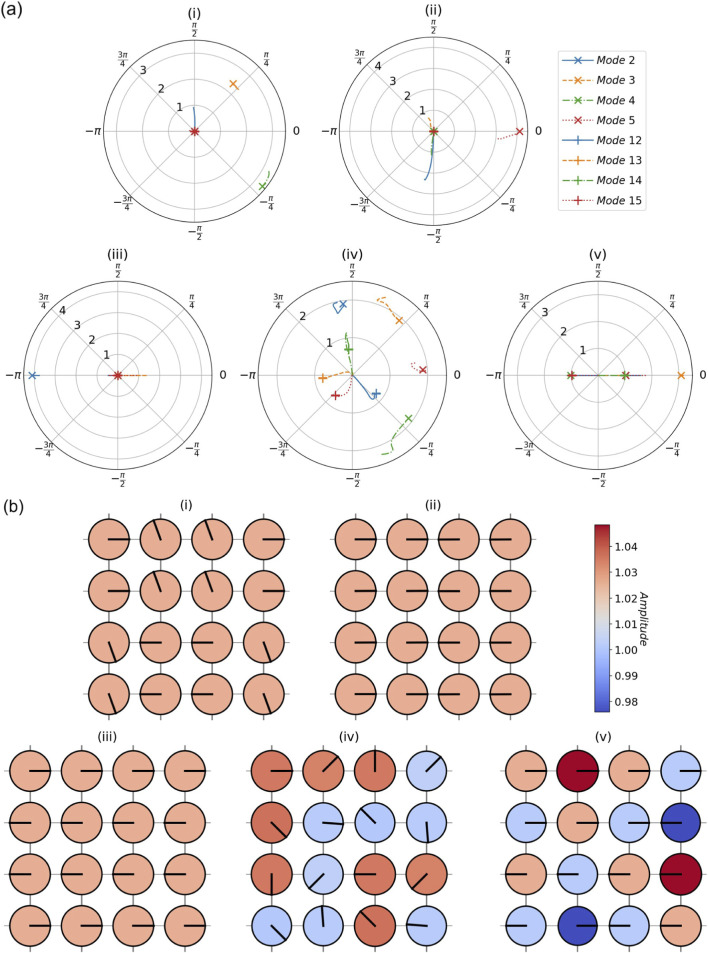
Continuation results from solution #30 with $\kappa_{f}=0.4$ in the Hadamard basis and the original basis, corresponding to cases (i)–(v) in Figure 3. **(a)** Continued curves in 2D polar coordinates, showing the amplitudes and phases of the relevant modes in the Hadamard basis. The markers, × and + , label the endpoints at τ=1 . **(b)** Equilibrium configurations in the original oscillator lattice at τ=1 . The amplitudes ρ are indicated by the radii and colors of the circles, and the phases ϕ are indicated by the directions of the hands. The local-minimum configuration in (i) has four locally synchronized clusters; the saddle configurations in (ii) and (iii) are Hadamard patterns. The saddle configurations in (iv) and (v) are more complex and might be difficult to obtain without continuation.

Figure 3 summarizes typical continuation trajectories from solution #30 with $\kappa_{f}=0.4$ . For each previously mentioned type (i)–(v), we plot the mode amplitudes r , phases θ , the equilibrium’s (negative) eigenvalues, and the energies H(r,θ;τ) as functions of the homotopy parameter τ∈[0,1] . The quantity $H_{0}\left(r\right)$ is also evaluated along the same curve and plotted together with H(r,θ;τ) to illustrate the magnitude of the θ -dependent cross terms in Equation 19 disregarded under the phase-decoupled approximation. The continuation in (i) leads to an equilibrium with no negative eigenvalues at τ=1 , which can be confirmed as an energy local minimum, while index-1 saddles are found in cases (ii) and (iii). These three equilibria share the same energy value H=−4.431 (which equals the initial energy $H_{0}$ ), and have one, three, and three additional zero eigenvalues, respectively, implying a degeneracy that will be discussed later. Meanwhile, index-3 and index-5 saddles with higher energies are obtained in (iv) and (v). To aid interpretation, we transform back to the Hadamard basis $E_{H}$ and visualize the continuation curves in mode-wise polar coordinates in Figure 4a. We then further reconstruct the corresponding oscillator configurations in the original coordinates (see Figure 4b). As a result, the equilibria obtained at τ=1 admit particularly simple representations in the Hadamard basis: case (i) involves two modes, while cases (ii) and (iii) each reduce to a single mode. Specifically, (ii) and (iii) correspond to the fifth and second Hadamard patterns in Figure 1c, while the local minimum in (i) is a quadrature superposition of the third and fourth Hadamard patterns, forming four locally synchronized clusters as shown in panel (i) of Figures 4a,b. We note that these results are representative, and other similar minima and saddles also exist owing to symmetry.

**FIGURE 5:**
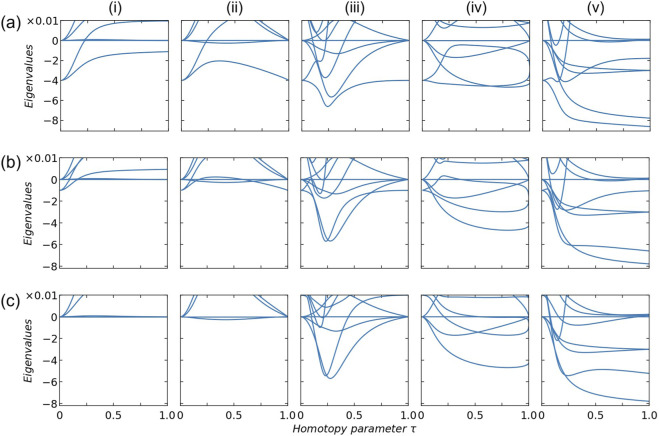
Negative eigenvalues of the equilibria as functions of τ∈[0,1] , corresponding to cases (i)–(v) in Figure 3, under different frustration strengths: **(a)** $\kappa_{f}=0.1$ , **(b)** $\kappa_{f}=0.4$ , and **(c)** $\kappa_{f}=0.7$ . Panel **(b)** is the same as Figure 3c and is reproduced here for convenient comparison. Increasing $\kappa_{f}$ lifts certain negative eigenvalues, sometimes above zero, at τ=1 , thereby decreasing the index of the equilibrium. Specifically, in case (i), the index decreases from 1 to 0 when $\kappa_{f}=0.4$ , and in cases (ii), (iii), and (v), the indices change from 1, 1, and 5 to 0, 0, and 4, respectively, when $\kappa_{f}=0.7$ .

Similar to the case of solution #1, the frustration strength $\kappa_{f}$ has a profound influence on the continuation from solution #30. Calculations show that, although the continuation curves r(τ) and θ(τ) differ only slightly from those in Figure 3, significant changes appear in the eigenspectra of the equilibria when $\kappa_{f}$ increases from 0.1 to 0.7, as shown in Figures 5a–c. In all cases, increasing $\kappa_{f}$ lifts certain eigenvalues toward positive values; consequently, the number of negative eigenvalues tends to decrease, resulting in increased stability (in the sense of fewer unstable directions in the state space). For example, the index-1 equilibrium at τ=1 in Figure 5a (case (i)) with $\kappa_{f}=0.1$ becomes index-0 in Figure 5b with $\kappa_{f}=0.4$ . Similar changes occur in (ii) and (iii) for stronger frustration $\kappa_{f}=0.7$ in Figure 5c, when the index-5 equilibrium in (v) becomes index-4.

#### 3.3.3 Solution #25

**FIGURE 6:**
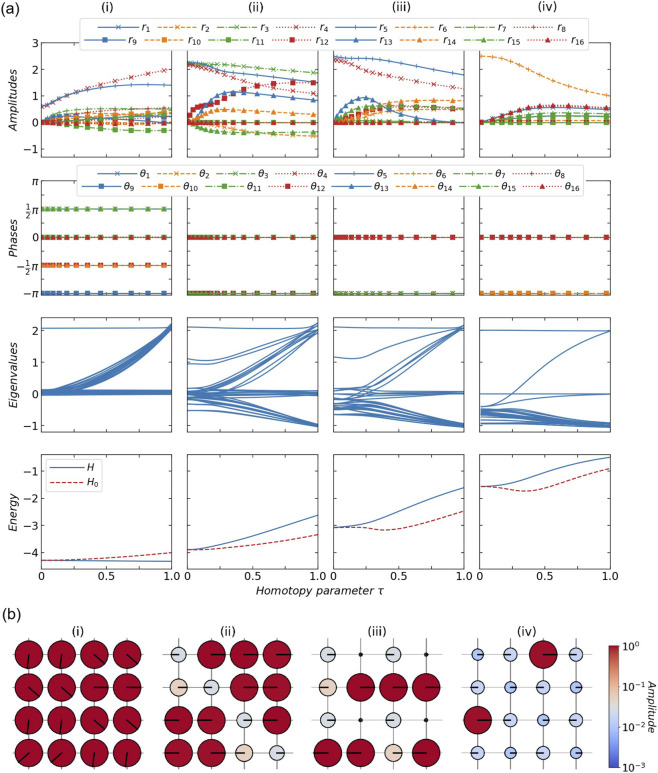
Representative continuation results initialized from solutions #25, #28, #24, and #32 with $\kappa_{f}=0.7$ , shown in columns (i), (ii), (iii), and (iv), respectively. **(a)** The amplitudes r , phases θ , eigenvalues of the equilibrium, and energy H(r,θ;τ) (together with $H_{0}\left(r\right)$ ) as functions of τ∈[0,1] . From (i) to (iv), we observe an overall increase in energy along with the increasing number of negative eigenvalues, indicating increasing instability. **(b)** Equilibrium configurations in the original oscillator lattice at τ=1 . The amplitudes ρ are indicated by the radii and colors of the circles on a log scale for better visualization. We note that, for these high-index saddles, many oscillators have very small but nonzero amplitudes.

This solution appears for sufficiently strong frustration such as $\kappa_{f}=0.7$ , where $A_{22}^{\left(0\right)}$ has a rank of 13 (and the coefficient matrix has rank 29), corresponding to a weak singularity. Two additional degrees of freedom emerge for satisfying the equilibrium condition to the first order: $\theta_{16}$ is unconstrained, and $\theta_{1},\theta_{2},\ldots ,\theta_{15}$ fall into two internally collinear groups with a free inter-group phase offset. We could find eight initial $\theta_{0}$ satisfying the equilibrium condition to the second order, among which two pairs of quadrature-locked $\theta_{0}$ extend to τ=1 . In these cases, the homotopy system exhibits no mode reduction, and the equilibria are initially index-4 saddles that become index-2 saddles at τ=1 . A typical continuation result, including the final oscillator configuration, is illustrated in column (i) of Figures 6a,b.

#### 3.3.4 Solutions #28, #24, and #32

These solutions all correspond to singular initialization where $A_{22}^{\left(0\right)}$ has a rank of 7 (the coefficient matrix has rank 23). All additional degrees of freedom reside in the phases of single modes. The homotopy system reduces to nonsingular 8-mode systems, with support {2,3,4,5,12,13,14,15} for solutions #28 and #24, or {1,6,7,8,9,10,11,16} for solution #32. For solution #28, we can find three collinear $\theta_{0}$ that satisfy the second-order equilibrium condition and extend to τ=1 . The equilibrium has index 16 initially and continues to index 10–16 at τ=1 . For solution #24, two such collinear $\theta_{0}$ are found; the equilibrium has index 24 initially and continues to index 20–23 at τ=1 . For solution #32, only one $\theta_{0}$ is found; starting with index 30 and $H_{0}=-1.567$ , the equilibrium still has a high index of 29 and a high energy H=−0.494 when reaching τ=1 . Varying $\kappa_{f}$ within [0.1,0.7] makes no fundamental difference. Typical continuation results, including the corresponding oscillator configurations, are shown for $\kappa_{f}=0.7$ in Figures 6a,b as cases (ii), (iii), and (iv).

### 3.4 Partial selectivity of the continuation

**FIGURE 7:**
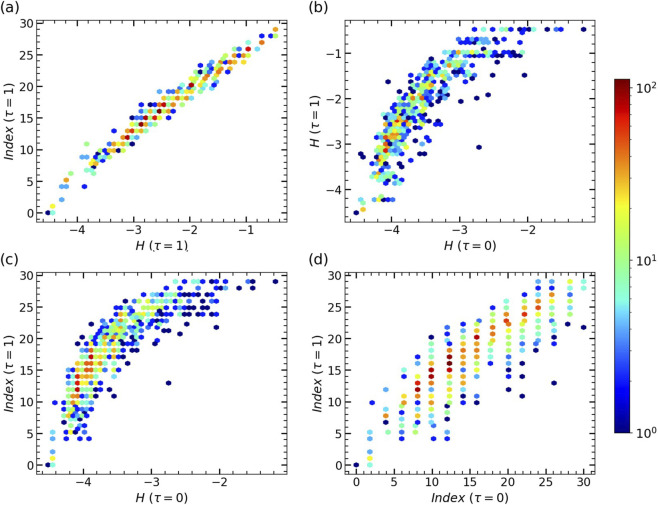
Partial selectivity of the equilibrium energy and index at τ=1 with respect to the initialization at τ=0 under our homotopy continuation. The data comprise about $2.6\times 10^{3}$ target equilibria obtained from about 360 approximate solutions $r_{0}$ at $\kappa_{\text{f}}=0.4$ , importance-sampled to yield an approximately uniform distribution of the initial energy H(τ=0) (i.e., $H_{0}\left(r_{0}\right)$ ). Log-scale hexagonal binning indicates point density in all panels. **(a)** Equilibrium index versus energy in the target system (τ=1) , showing an approximately linear trend. **(b)** Target energy H(τ=1) (i.e., $H{\left(r,\theta ;\tau \right)}_{\tau =1}$ ) versus initial energy H(τ=0) , exhibiting a positive association. **(c)** Target index versus initial energy, exhibiting a positive association. **(d)** Target index versus initial index; despite substantial scatter, a clear positive correlation persists.

From the preceding results, we have observed a possible selectivity in the energy and index of the equilibria reached by continuation: starting from an approximate solution $r_{0}$ at τ=0 with relatively high (or low) energy, the equilibria obtained at τ=1 also tend to have relatively high (or low) energy and indices. To examine this effect, we performed homotopy continuation from a collection of approximate solutions randomly sampled from the full set. Specifically, for $\kappa_{f}=0.4$ , we resampled about 430 approximate solutions (i.e., 10% of the total) using importance sampling to obtain an approximately uniform distribution of the initial energy $H_{0}$ . Among these, about 360 equilibria $r_{0}$ with about $1.2\times 10^{4}$ initial phases $\theta_{0}$ (among a total of $1.3\times 10^{4}$ ) produce about $2.6\times 10^{3}$ equilibria at τ=1 . The results are summarized in the hexagonal binning plots shown in Figure 7.

Figure 7a shows that, in the target system (τ=1) , the index and energy of an equilibrium are positively associated with a roughly linear trend; both global and local index-0 minima are also evident from the plot. Figures 7b,c further indicate that both the target energy $H{\left(r,\theta ;\tau \right)}_{\tau =1}$ (or H(τ=1) for brevity) and the target index increase with the initial energy $H_{0}\left(r_{0}\right)$ (or, H(τ=0) ). Finally, Figure 7d shows the comparison of the target and initial indices: although the relationship exhibits substantial scatter, a clear positive correlation remains. While Figure 7 is based on continuation at $\kappa_{f}=0.4$ with E chosen as in Figure 1e, qualitatively similar behavior is observed for other random bases and other values of $\kappa_{f}$ of interest. Together, these observations suggest partial selectivity of this eigenspace-based homotopy continuation procedure, in the sense that it preferentially finds equilibria with higher (or lower) energy and, correspondingly, higher (or lower) index, depending on the initialization energy. Such selectivity may greatly facilitate the exploration of low-index critical points, including minima and index-1 saddles, which usually represent macrostates and transition mediators.

The observed partial selectivity is closely related to how the approximation is constructed. The phase-decoupled approximation is formulated in the eigenspace coordinates of the linear part of the dynamics and is not an arbitrary surrogate. In this representation, the effect of the linear coupling term, which is central to shaping the collective energy landscape, is already partially incorporated, albeit at the cost of a modified higher-order structure. Therefore, the approximate and full landscapes remain structurally close in the sense relevant to continuation, and the homotopy acts as a structured deformation rather than a complete reorganization, along which some properties, such as the energy and index, undergo only limited changes. Consequently, equilibria with a relatively low or high energy and index in the approximation tend to continue to equilibria with similar properties in the full system.

### 3.5 Coverage of low-index equilibria

To better understand the multistability of the system, we usually aim to identify as many low-index equilibria as possible. However, in the presence of repeated eigenvalues of W , initialization based on a single randomly rotated E may reveal only a subset of equilibria in the full system, as illustrated by the foregoing results. Moreover, the minima and index-1 saddles shown in Figure 3 possess additional zero eigenvalues and identical energy, indicating degeneracy and possibly non-isolation. We, therefore, perform a coverage analysis of the low-index equilibria obtained by continuation from low-energy approximate solutions across repeated continuation trials with different random bases E (up to roughly 200 choices of E and $8\times 10^{3}$ trials). To identify equilibria while properly accounting for the invariance under global phase shifts, we define the distance between two equilibria $z_{1}=r_{1}◦\exp\left(i\theta_{1}\right)$ and $z_{2}=r_{2}◦\exp\left(i\theta_{2}\right)$ by

$$\begin{array}{cc} D\left(z_{1},z_{2}\right) & :=\underset{\gamma \in R}{\min}\left\|z_{1}-\exp\left(i\gamma \right)z_{2}\right\|\\ & =\sqrt{{\left\|z_{1}\right\|}^{2}+{\left\|z_{2}\right\|}^{2}-2|z_{1}^{†}z_{2}|}\\ & =\sqrt{{\left\|r_{1}\right\|}^{2}+{\left\|r_{2}\right\|}^{2}-2\left|{\left(r_{1}◦r_{2}\right)}^{⊤}\exp\left[i\left(\theta_{1}-\theta_{2}\right)\right]\right|}, \end{array}$$ (49)

where the minimum is attained at $\gamma =\gamma_{*}=-\arg\left(z_{1}^{†}z_{2}\right)$ . In other words, $D\left(z_{1},z_{2}\right)$ is the minimum Euclidean distance in state space between the two deterministic oscillatory solutions represented by $z_{1}$ and $z_{2}$ . It is also invariant under the orthonormal transformation $z=E^{⊤}s$ . We adopt a numerical threshold value $D_{th}=1\times 10^{-3}$ to determine whether a newly obtained equilibrium should be recorded as distinct. Because there is no prior knowledge of which equilibrium will be obtained in each trial, we treat the process as a finite-population discovery model under sampling with replacement. In each continuation trial, a particular equilibrium type is drawn with probability p . The count q of distinct equilibria discovered then increases with the number of draws (trials) m and eventually saturates. The expected value of q(m) is given by

$$E\left[q\left(m\right)\right]=n\left[1-{\left(1-pn^{-1}\right)}^{m}\right]\approx n\left[1-\exp\left(-mpn^{-1}\right)\right],$$ (50)

where n denotes the total number of distinct equilibria of interest. Here, the number of draws m may be defined either as the number of continuation trials that successfully reach τ=1 or as the total number of continuation trials including those that terminate without yielding an equilibrium, with corresponding interpretation of p . We adopt the former convention in the following analysis. For convenience, we use subscripts on D and q to label the starting solution for continuation, and we use superscripts to denote the indices of the target equilibria in the discussion below.

**FIGURE 8:**
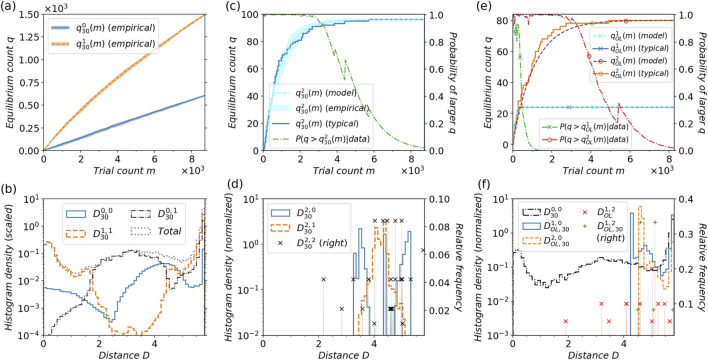
Coverage analysis of low-index equilibria obtained by repeated continuation with various bases E , using the distance D defined by Equation 49 and the discovery model (Equation 50). **(a)** Distinct counts $q_{30}^{0}\left(m\right)$ and $q_{30}^{1}\left(m\right)$ of index-0 and index-1 equilibria after m continuation trials from solution #30 at $\kappa_{f}=0.4$ , shown as means with sample standard deviations. The lack of saturation suggests non-isolation. **(b)** Histogram densities of the within-group distances $D_{30}^{0,0}$ and $D_{30}^{1,1}$ and the between-group distances $D_{30}^{0,1}$ for equilibria obtained in **(a)**. The density near $D_{30}^{0,1}=0$ suggests adjacency between the index-0 and index-1 sets, and the multimodal within-group distributions suggest isolated substructures. **(c,d)** Corresponding results for index-2 equilibria continued from solution #30 at $\kappa_{f}=0.4$ : the saturating $q_{30}^{2}\left(m\right)$ , which is well modeled by (n,p)=(96,0.106) , and the discrete-valued $D_{30}^{2,2}$ indicate isolated equilibria. The posterior probability for more such equilibria being undiscovered decreases to about 0.02 by the end of a typical search. **(e,f)** Coverage analysis at $\kappa_{f}=0.7$ for continuation from the lowest-energy solution #30 and the three next-lowest-energy solutions (collectively denoted by OL). The histogram of $D_{30}^{0,0}$ reproduces the overall one in **(b)**, suggesting stabilization of all previously found index-1 equilibria. Additional index-1 and index-2 equilibria are found, with $q_{OL}^{1}\left(m\right)$ and $q_{OL}^{2}\left(m\right)$ well modeled by (n,p)=(24,0.187) and (n,p)=(80,0.073) , respectively. Finally, we note that the data in panels **(a,e)** were collected from about 200 random bases; in these two cases, approximately $8.7\times 10^{3}$ and $8.3\times 10^{3}$ continuation trials reached τ=1 , of a total of $1.0\times 10^{4}$ and $2.1\times 10^{4}$ continuation trials, respectively.

At the frustration strength $\kappa_{f}=0.4$ , we focus on continuation from approximate solution #30, which persists under any randomly rotated E and consistently has the lowest initial energy except for the non-branching starting solution #1. Figure 8a plots the distinct counts $q_{30}^{0}\left(m\right)$ and $q_{30}^{1}\left(m\right)$ of index-0 and index-1 equilibria obtained after m continuation trials, shown as mean values with bootstrapped sample standard deviations. Both $q_{30}^{0}\left(m\right)$ and $q_{30}^{1}\left(m\right)$ increase with little tendency of saturation, and the resultant target equilibria all have two or four zero eigenvalues (i.e., one or three eigenvalues modulo the global phase shift); fitting Equation 50 is either unsuccessful or yields quite large n , suggesting non-isolated equilibria. Figure 8b shows the histogram densities of the distances $D_{30}^{0,0}$ among index-0 equilibria, $D_{30}^{1,1}$ among index-1 equilibria, and $D_{30}^{0,1}$ between index-0 and index-1 equilibria, all of which exhibit continuous distributions. The noticeable density for small values of $D_{30}^{0,1}$ suggests adjacency between the index-0 and index-1 sets, while the multimodal distribution of $D_{30}^{0,0}$ and $D_{30}^{1,1}$ suggests isolated substructures within them. For comparison, Figures 8c,d show a saturating sequence $q_{30}^{2}\left(m\right)$ and discrete values of $D_{30}^{2,2}$ for index-2 equilibria continued from solution #30, all with no additional zero eigenvalues, suggesting that they are isolated. The empirical $q_{30}^{2}\left(m\right)$ profiles are well described by the discovery model with n=96 and p=0.106 ; for a typical sequence, we also computed the posterior probability that $n>q_{30}^{2}\left(m\right)$ under this model and found that it decreases to about 0.02. This suggests that it is unlikely that more than these 96 index-2 equilibria remain undiscovered.

Coverage analysis is also conducted for low-index equilibria at $\kappa_{f}=0.7$ , as summarized in Figures 8e,f. In this case, the energy ordering of approximate solutions #1 and #30 reverses, as shown previously in Figure 2. Continuation from the minimum-energy solution #30 again produces 96 index-2 equilibria, together with a set of index-0 equilibria whose distance histogram resembles the overall one in Figure 8b. This suggests that the stabilization shown in Figure 5 has occurred for all previously identified index-1 equilibria. Meanwhile, we also investigate continuation from the three approximate solutions with the next-lowest energies after #30 under various random choices of E (with labels ranging from ∼10 to ∼20000 , collectively denoted by “OL”). As shown in Figure 8e, we find 24 isolated index-1 equilibria and an additional 80 isolated index-2 equilibria, for which the posterior probabilities that more remain undiscovered decrease to nearly 0 and 0.012, respectively. These index-1 saddles, all with H=−4.328 , may lie on the ridges separating multiple subsets of the equi-energy index-0 minima at H=−4.447 . Our observations in this subsection also clarify that the discovery behavior can be meaningfully characterized only when the target equilibria are isolated modulo the global phase shift, as indicated by the absence of additional zero eigenvalues. We note that, in such cases, Equation 50 can be fitted once a clear saturation trend appears, even before full saturation is reached, to estimate the total number n of distinct equilibria.

### 3.6 Energy landscapes: embedding-based visualization

**FIGURE 9:**
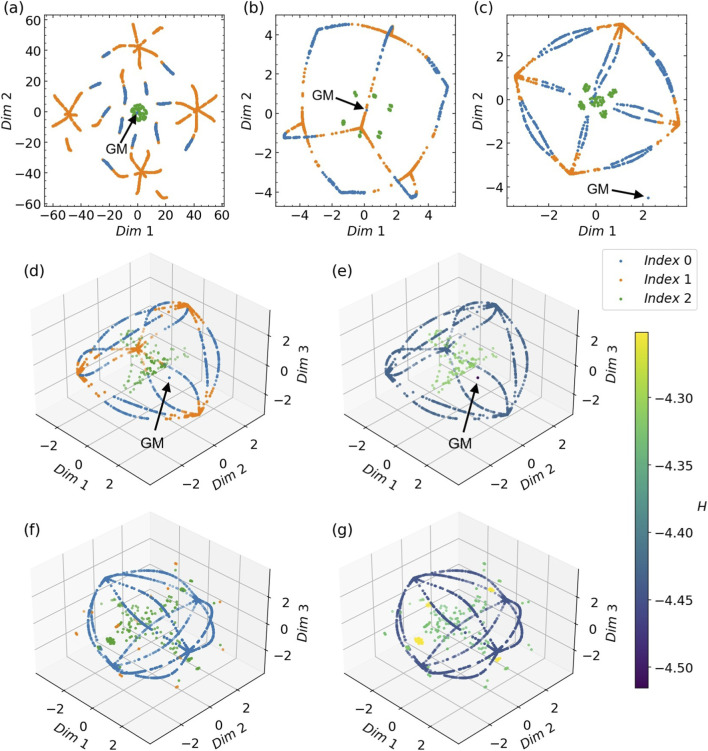
Manifold embeddings of low-index equilibria. At $\kappa_{f}=0.4$ , two-dimensional embeddings with points labeled by their indices are obtained with several commonly used methods: **(a)** tSNE, which resolves local structures including four six-spoked, asterisk-shaped sets of index-1 equilibria and 12 segments of index-0 equilibria; **(b)** Isomap, which reveals the global structure of a complete graph on four vertices, where index-0 equilibria occupy the middle parts of its six edges; **(c)** MDS, which clearly recovers the full organization as a complete multigraph with four vertices and double edges between each pair of vertices. The vertices correspond to the Hadamard modes (basis vectors) for the second largest eigenvalue of L , and points on the edges correspond to phase-offset superpositions of two modes. Three-dimensional MDS embeddings are shown in **(d)** for $\kappa_{f}=0.4$ , revealing the structure in **(c)** more clearly; **(e)** corresponds to **(d)**, with energy values indicated by color; **(f)** for $\kappa_{f}=0.7$ , revealing an entire stabilized multigraph of index-0 equilibria, with newly appearing index-1 and index-2 equilibria; and **(g)** corresponds to **(f)**, with energy values indicated by color. In **(a**–**e)**, the isolated global minimum (GM) is also marked. The manifold embedding here was implemented using scikit-learn 1.8.0 with Python 3.12.7.

After we have identified the low-index equilibria, we next investigate their geometric organization using manifold embedding (nonlinear dimensionality reduction), using the pre-computed distances defined in Equation 49. In principle, manifold embedding seeks a low-dimensional Euclidean representation that preserves these distances as well as possible. Commonly used methods include t -distributed stochastic neighbor embedding (tSNE), multidimensional scaling (MDS), and Isomap. Figures 9a–c show the embeddings of the low-index equilibria at $\kappa_{f}=0.4$ , obtained using two-dimensional tSNE, Isomap, and MDS, respectively. We find that tSNE, which is well suited to capturing local structures, reveals four six-spoked, asterisk-shaped sets of index-1 equilibria and 12 segments of connected index-0 equilibria, although the global arrangement of these sets appears irregular. In contrast, Isomap reveals a cleaner global structure, namely, the skeleton of a 3-simplex, or a complete graph on four vertices $\left(K_{4}\right)$ , where the index-1 equilibria occupy regions near its vertices and the index-0 equilibria lie in each of its six edges. The geometry of the full equi-energy set of index-0 and index-1 equilibria is most clearly recovered by MDS, which yields a complete multigraph with four vertices and 12 edges; this structure is captured even more clearly by three-dimensional MDS, as shown in Figures 9d,e.

These results are also consistent with the number of additional zero eigenvalues found previously in Figure 5, namely, one flat direction for the index-0 equilibrium and three for the index-1 equilibria. We also confirm that the vertices represent the Hadamard modes or basis vectors in $E_{H}$ corresponding to the second largest eigenvalue of L (with multiplicity 4), and a point on an edge represents the superposition of two Hadamard modes with certain amplitudes and phase offset Δθ ; this has been suggested by previous results shown in Figure 4. At $\kappa_{f}=0.7$ , three-dimensional MDS reveals a similar geometric organization, in which the entire graph is stabilized, and newly appearing isolated index-1 equilibria emerge around it, as shown in Figures 9f,g. We therefore infer that, at intermediate frustration, the energy landscape mainly consists of an isolated global minimum (i.e., the full synchronization) and 12 flat 1D troughs (i.e., proper superposition of two Hadamard modes with sufficiently large |Δθ| ), separated by unstable breaches. At sufficiently large frustration, these breaches heal, and the troughs merge into a connected set of global minima.

### 3.7 Simulated dynamics: synergetics and transitions

#### 3.7.1 Spatiotemporal dynamics under noise

The results above show that varying the frustration strength $\kappa_{f}$ profoundly deforms the energy landscape of the coupled oscillator network. Under sufficiently weak frustration, the fully synchronized state is the unique energy minimum. As $\kappa_{f}$ increases, the synchronized state gradually loses stability, while some low-index, low-energy saddles on a complete multigraph progressively stabilize. At an intermediate frustration level (e.g., $\kappa_{f}=0.4$ ), saddles in the middle portions of the edges become index-0 and coexist with the synchronized state as additional local minima. For stronger frustration (e.g., $\kappa_{f}=0.7$ ), all saddles on the graph turn into index-0 equilibria, whereas the synchronized state turns into a saddle. In contrast, the high-index saddles undergo no qualitative change over this range, and we do not consider larger values of $\kappa_{f}$ in this article. Such an energy-based structure supports rich spatiotemporal dynamics, including emergent (synergetic) rhythms and noise-driven state transitions.

**FIGURE 10:**
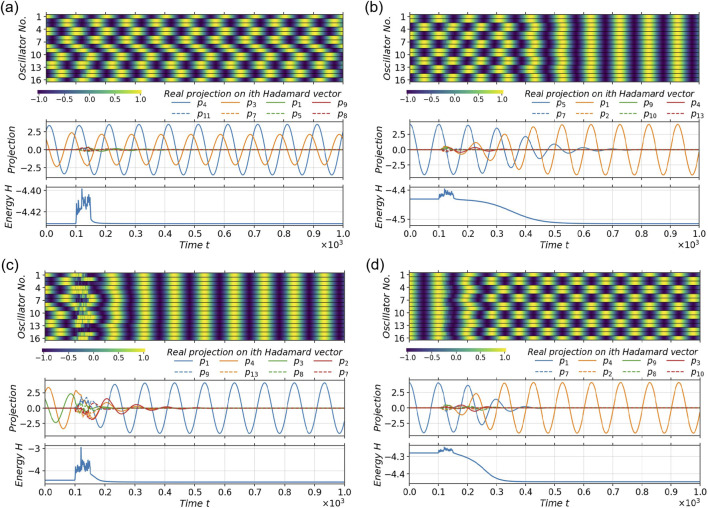
Typical spatiotemporal dynamics of the coupled oscillator network in response to transient noise, simulated with δt=0.01 under different conditions. In each subfigure, the upper, middle, and lower panels show, respectively, the spatiotemporal patterns Re s (oscillators stacked vertically), the projections onto the Hadamard basis vectors $p_{i}=\text{Re }{\left(E_{H}^{⊤}s\right)}_{i}$ , and the energy H , all as functions of time t . In all cases, i.i.d. Gaussian noise is applied over t∈[100,150] . **(a)** $\kappa_{f}=0.4$ and σ=0.05 , initialized at the index-0 equilibrium (i) in Figure 3: small transient noise only causes slight changes in the amplitudes and phase difference of its constituent modes, indicating stability transverse to the trough and neutral stability along it. **(b)** $\kappa_{f}=0.4$ and σ=0.05 , initialized at the index-1 saddle (ii) in Figure 3: even small noise induces synchronization. **(c)** $\kappa_{f}=0.4$ and σ=0.3 , initialized as in **(a)**: large noise drives the network out of the energy trough and induces synchronization. **(d)** $\kappa_{f}=0.7$ and σ=0.05 , initialized at the synchronized index-4 saddle: a small noise perturbation breaks synchronization.

**FIGURE 11:**
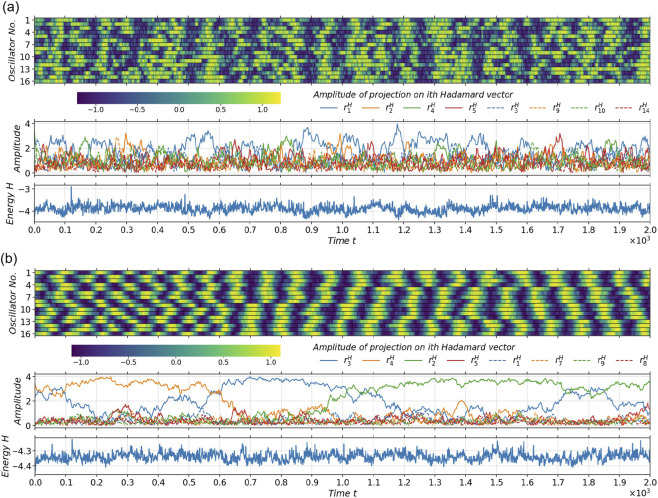
Typical spatiotemporal dynamics of the coupled oscillator network under stationary noise, simulated with δt=0.01 . In each subfigure, the upper and lower panels show the same quantities as in Figure 10, while the middle panel shows the amplitudes $r_{i}^{H}$ of the projections onto the Hadamard basis vectors, as functions of time t . **(a)** $\kappa_{f}=0.4$ , and σ=0.3 . The network does not attain ideal synchronization but intermittently visits highly coherent patterns (with large $r_{1}^{H}$ ), interspersed with partial approaches to other Hadamard patterns. **(b)** $\kappa_{\text{f}}=0.7$ , and σ=0.12 . Under stationary noise, the system exhibits excursions on the graph of energy troughs, with clear visits to the vertices (where a single $r_{i}^{H}$ , i∈{2,3,4,5} , is large) and motion along the edges (where two $r_{i}^{H}$ are appreciable).

To illustrate this, we numerically integrated Equation 1 using the Euler–Maruyama scheme with time step δt=0.01 . Figures 10, 11 show representative spatiotemporal patterns, together with the corresponding time evolution of the energy and the projections (or their amplitudes) onto the Hadamard basis vectors, to aid visualization of transitions. In Figures 10a–d, we apply transient noise over the interval t∈[100,150] . For $\kappa_{f}=0.4$ , the network initialized at the index-0 equilibrium (i) in Figure 3 remains near its initial state under a small perturbation ( σ=0.05 ; Figure 10a); meanwhile, the amplitudes of the two constituent Hadamard modes and their phase offset undergo slight changes before and after the perturbation. This is consistent with a local minimum in a trough, where small transient noise induces a slight displacement along the flat direction. By contrast, when initialized at the index-1 saddle (ii) in Figure 3, which is in a breach, the system lingers there for some time but readily relaxes to synchronization after a small perturbation (Figure 10b). A substantially larger perturbation is required to induce synchronization from the index-0 local minimum ( σ=0.3 ; Figure 10c), which drives the trajectory out of the current energy trough. For $\kappa_{f}=0.7$ , synchronization itself is a saddle, and even small noise induces desynchronization ( σ=0.05 ; Figure 10d), in contrast to the noise-induced synchronization in Figure 10b. In Figure 11, stationary i.i.d. Gaussian noise is applied over the entire time range. For $\kappa_{f}=0.4$ and relatively large noise σ=0.3 , the spatiotemporal dynamics appears to be consistent with a Gibbs (Boltzmann) distribution: the global energy minimum (synchronization) is visited transiently from time to time, interspersed with visits to other patterns. For $\kappa_{f}=0.7$ and intermediate noise (σ=0.12) , the network exhibits persistent wandering on the graph of troughs: typically, two Hadamard modes attain appreciable amplitudes at the same time, indicating a mixed state on an edge between two vertices, while the energy is approximately maintained within [−4.4,−4.3] .

#### 3.7.2 Stationary transition kinetics

In this subsection, we conduct a simple investigation of the system’s transition mechanisms through the transition kinetics of its stationary dynamics, focusing in particular on their scaling with the noise level. We adopt a simple relative-distance-based partition of the state space, with a hysteresis rule to reduce flickering. Specifically, let $D_{i}\left(t\right)$ denote the distance from the network state to the index-0 equilibrium set $S_{i}$ ; when $D_{i}\left(t_{1}\right)<\alpha \min_{j\neq i}D_{j}\left(t_{1}\right)$ , the system is classified as entering macrostate i at time $t_{1}$ . It is then labeled as exiting macrostate i at the first time $t_{2}>t_{1}$ such that $D_{i}\left(t_{2}\right)>\beta \min_{j\neq i}D_{j}\left(t_{2}\right)$ . The sojourn time $T_{s}$ , defined as the time spent in a macrostate from entry to exit, and the first-passage time $T_{p}$ from macrostate i to j , defined as the time from entry into macrostate i to the first subsequent entry into macrostate j , can then be analyzed statistically. We numerically integrated Equation 1 with time step δt=0.1 over a duration up to $\Delta t=2\times 10^{5}$ (after discarding a transient length of $5\times 10^{3}$ ), and obtained the following results.

**FIGURE 12:**
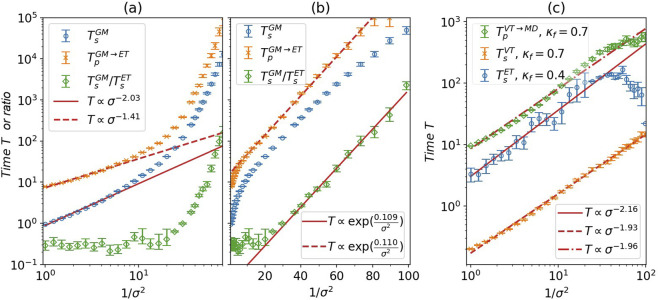
Transition kinetics of the stationary dynamics under a relative-distance-based state-space partition with hysteresis. **(a,b)** Uphill kinetics from the global minimum (GM) to the union of all energy troughs (ET) at $\kappa_{f}=0.4$ . At small noise, $T_{s}^{\text{GM}}/T_{s}^{\text{ET}}$ and $T_{p}^{\text{GM}\to \text{ET}}$ follow Arrhenius–Kramers-type scaling, with an effective energy difference ${\left(\Delta H\right)}_{\mathrm{eff}}\approx 0.055$ estimated as halves of the slopes of the linear fits in the semi-log plots in **(b)**. Note that the vertical axis here is plotted on a $\log_{10}$ scale for readability; accordingly, the fitted slope reported in the legend differs from the apparent slope on the plot by a constant conversion factor of ln⁡10 . At larger noise $\left(1/\sigma^{2}<6\right)$ , $T_{s}^{\text{GM}}$ and $T_{p}^{\text{GM}\to \text{ET}}$ are better described by power laws, as indicated by the linear fits in the log-log plots in **(a)**, suggesting diffusion-dominated behavior. **(c)** Kinetics on the equi-energy graph, shown by $T_{s}^{\text{ET}}$ at $\kappa_{f}=0.4$ and by the sojourn time $T_{s}^{\text{VT}}$ near a vertex and the first-passage time $T_{p}^{\text{VT}\to \text{MD}}$ from a vertex to a nearest edge midpoints at $\kappa_{f}=0.7$ . In these cases, the approximate power-law scaling $T\propto \sigma^{-2}$ is consistent with diffusion on the graph.

For $\kappa_{f}=0.4$ , we adopt a coarse-grained description in which the union of all energy troughs is treated as a single macrostate (ET), together with the macrostate corresponding to the global minimum (GM), using α=0.7 and β=1.0 . Figures 12a,b show the sojourn time in GM, $T_{s}^{\text{GM}}$ , the first-passage time from GM to ET, $T_{p}^{\text{GM}\to \text{ET}}$ , and the ratio of sojourn times $T_{s}^{\text{GM}}/T_{s}^{\text{ET}}$ as functions of $1/\sigma^{2}$ , plotted on log–log and semi-log scales, respectively. For relatively small σ , both $T_{s}^{\text{GM}}/T_{s}^{\text{ET}}$ and $T_{p}^{\text{GM}\to \text{ET}}$ can be well described by Equation 51:

$$\frac{T_{s}^{\text{GM}}\left(\sigma \right)}{T_{s}^{\text{ET}}\left(\sigma \right)}\propto \exp\left[\frac{2{\left(\Delta H\right)}_{\mathrm{eff}}^{\left(1\right)}}{\sigma^{2}}\right],\;T_{p}^{\text{GM}\to \text{ET}}\left(\sigma \right)\propto \exp\left[\frac{2{\left(\Delta H\right)}_{\mathrm{eff}}^{\left(2\right)}}{\sigma^{2}}\right],$$ (51)

as predicted by simple effective two-state models with effective energy differences ${\left(\Delta H\right)}_{\mathrm{eff}}^{\left(1\right)}$ and ${\left(\Delta H\right)}_{\mathrm{eff}}^{\left(2\right)}$ . In practice, these effective energy differences are estimated by fitting a straight line to $\ln\left(T_{s}^{\text{GM}}/T_{s}^{\text{ET}}\right)$ *versus* $1/\sigma^{2}$ or $\ln T_{p}^{\text{GM}\to \text{ET}}$ *versus* $1/\sigma^{2}$ over the low-noise regime, with ${\left(\Delta H\right)}_{\mathrm{eff}}$ estimated as one-half of the fitted slope. In the present case, we obtained ${\left(\Delta H\right)}_{\mathrm{eff}}^{\left(1\right)}=\left(5.45\pm 0.17\right)\times 10^{-2}$ over $1/\sigma^{2}\in \left[36,100\right]$ and ${\left(\Delta H\right)}_{\mathrm{eff}}^{\left(2\right)}=\left(5.49\pm 0.05\right)\times 10^{-2}$ over $1/\sigma^{2}\in \left[9,81\right]$ . These results are consistent with an Arrhenius–Kramers law, indicating thermally activated escape. The effective energy differences are smaller than the actual energy gap of 0.0846 between the global minimum and the equi-energy graph, likely because of the coarse partition of the state space to define the two macrostates. In contrast, for sufficiently large σ , $T_{s}^{\text{GM}}$ and $T_{p}^{\text{GM}\to \text{ET}}$ appear to follow power-law relations given in Equation 52:

$$T_{s}^{\text{GM}}\left(\sigma \right)\propto {\left(\frac{1}{\sigma^{2}}\right)}^{C_{1}},\;T_{p}^{\text{GM}\to \text{ET}}\left(\sigma \right)\propto {\left(\frac{1}{\sigma^{2}}\right)}^{C_{2}},$$ (52)

with $C_{1}=1.01\pm 0.03$ and $C_{2}=0.703\pm 0.027$ over $1/\sigma^{2}\in \left[1,6\right]$ . This behavior, with an exponent close to 1, suggests diffusion-dominated motion under strong noise. In contrast, the smaller exponent $C_{2}$ may reflect the increasing difficulty of reaching ET as the accessible state space and the effective transition region expand for large σ . Moreover, the sojourn time $T_{s}^{\text{ET}}$ shown in Figure 12c exhibits diffusion-like behavior over a broad range $1/\sigma^{2}\in \left[1,49\right]$ . A fit of the form ${\left(1/\sigma^{2}\right)}^{C_{3}}$ gives $C_{3}=1.08\pm 0.04$ , indicating diffusion-limited escape from ET. Meanwhile, the decrease of $T_{s}^{\text{ET}}$ for $1/\sigma^{2}>49$ is possibly attributable to the imperfect state-space partition, whereby thermally driven excursions outside the troughs can be counted as stays in, or escapes from, ET.

For the larger frustration $\kappa_{f}=0.7$ , at which the entire multigraph forms a connected trough, no natural distinction between metastable states remains. Nevertheless, we can still examine the residence within prescribed regions, or quasi-states. To do so, we naïvely partition the state space to form 16 quasi-states, associated with the four vertices and 12 edge midpoints, using the common parameters α=0.95 and β=1.0 . The sojourn time $T_{s}^{\text{VT}}$ near a vertex and the first-passage time from a vertex to one of its nearest edge midpoints $T_{p}^{\text{VT}\to \text{MD}}$ are also shown in Figure 12c. Both quantities exhibit nearly linear behavior in the log–log plot over almost the full investigated range $1/\sigma^{2}\in \left[1,100\right]$ , consistent with the previously mentioned scaling relation with fitted exponents $C_{4}=0.966\pm 0.008$ and $C_{5}=0.979\pm 0.008$ , respectively. This supports the interpretation of diffusion motion on the graph. Overall, despite possible inaccuracies introduced by the naïve partition of the state space, these results suggest an Arrhenius–Kramers-like scaling for uphill escape at low noise and a diffusion-dominated scaling for motion at large noise or on the equi-energy graph; these mechanisms are also consistent with the previously obtained energy landscapes.

### 3.8 Phase-decoupled approximation revisited

**FIGURE 13:**
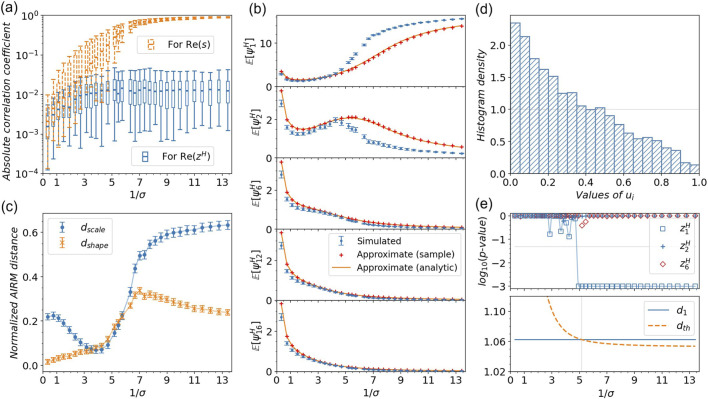
Statistical assessment of the phase-decoupled approximation for the projected oscillator dynamics $z^{H}=E_{\text{H}}^{⊤}s$ at $\kappa_{f}=0.4$ . **(a)** Absolute Pearson correlation coefficients between different components of Re (s) and $\text{Re }\left(z^{\text{H}}\right)$ , as functions of 1/σ ; the boxes show the mean values and [0.25,0.75] quantile ranges, and the whiskers show the [0.05,0.95] quantiles. The weak correlation of the projected components supports the approximate simultaneous diagonalization assumption. **(b)** Comparison of $E\left[\psi_{i}^{\text{H}}\right]$ obtained from simulation and from the approximation (by sampling or analytically) for selected modes, showing qualitative agreement but delayed growth of $E\left[\psi_{1}^{\text{H}}\right]$ in the approximation. **(c)** Scale and shape components of the dimension-normalized AIRM distance between the simulated covariance Cov (Re (s)) and that predicted by the approximation. The error is mainly in scale, and the shape mismatch remains comparatively small. **(d)** Histogram of PIT values $u_{i}$ for $\psi^{\text{H}}$ at σ=0.25 , showing deviation from the uniform distribution expected under exact conditional truncated normality. **(e)** Threshold for stochastic oscillation death. The simulated loss of unimodality of $z_{1}^{\text{H}}$ near 1/σ≈4.9 , indicated by Hartigan’s dip test (in the top panel), agrees closely with the theoretical threshold $\sigma_{th}=5.15$ predicted by the approximation, corresponding to $d_{1}=d_{th}$ in the bottom panel.

Finally, we return to the phase-decoupled approximation used to initiate the continuation and revisit its performance in describing the statistical properties of the oscillator network dynamics. Specifically, we examine the modeled properties of $z^{\text{H}}$ and compare them with those computed from the projections $z^{H}=E_{H}^{⊤}s$ with simulated s . Here, we fix $\kappa_{f}=0.4$ and focus on the following aspects:

- *Simultaneous diagonalization.* The phase-decoupled approximation implicitly assumes simultaneous diagonalization of the coupling matrix W and the covariance matrix Cov (Re (s)) or Cov (Im (s)) because the amplitude dynamics (Equation 11) does not favor any particular phases and thus predicts uncorrelated components of $z^{\text{H}}$ . Figure 13a shows the ranges of the absolute Pearson correlation coefficients for pairs of components of Re (s) and $\text{Re }\left(z^{\text{H}}\right)=E_{H}^{⊤}\text{Re }\left(s\right)$ obtained from the simulation. Even in the deterministic limit, approximately 95% of these correlation coefficients after projection onto $E_{\text{H}}$ have absolute values below 0.04, supporting this assumption.
- *Variance of* $\text{Re }\left(z^{\text{H}}\right)$ *,* that is, $\frac{1}{2}E\left[\psi^{\text{H}}\right]$ $\left(\psi^{H}=r^{H}◦r^{H}\right)$ *.* The approximate energy model (Equation 13) induces the exponential-family distribution $P_{\mathrm{approx}}^{\left(\psi \right)}\left(\psi \right)\propto \exp\left(-2H_{\mathrm{approx}}^{\left(\psi \right)}\left(\psi \right)/\sigma^{2}\right)$ for ψ . This remains valid even when $B^{⊤}B$ is singular, provided that the distribution exists, that is, it is integrable. For the network and basis $E_{H}$ considered here, this gives rise to the degenerate distribution Equation 17, which can be sampled numerically using a Metropolis–Hastings sampler with a ridge-perturbed random-scan Gibbs proposal, or treated analytically as in Li et al. (2022). Figure 13b shows the comparison of $E\left[\psi_{i}^{\text{H}}\right]$ ( i=1,2,6,12,16 for different eigenvalues) calculated from simulated $z^{\text{H}}=E_{H}^{⊤}s$ with the corresponding values obtained from the approximation (either analytically or by sampling). The phase-decoupled approximation captures the qualitative changes in the variances but exhibits a substantial lag in the growth of the dominant mode as the noise level decreases, similar to the results in Li et al. (2022).
- *Covariance* Cov (Re (s)) *.* Under the phase-decoupled approximation, the covariance matrix of the oscillators is given by $\text{Cov }\left(\text{Re }\left(s\right)\right)=\frac{1}{2}E_{H}\text{Diag }\left(E\left[\psi^{\text{H}}\right]\right)E_{H}^{⊤}$ . We compare this with the covariance matrix obtained from simulation, using the dimension-normalized affine-invariant Riemannian metric (AIRM) distance for positive definite matrices $M_{1},M_{2}\in R^{n\times n}$ , defined as Equation 53:
$$d_{\text{nAIRM}}\left(M_{1},M_{2}\right):={\left[\frac{1}{n}\overset{n}{\underset{i=1}{\sum }}{\left(\ln\lambda_{i}\right)}^{2}\right]}^{1/2}=\sqrt{d_{\text{scale}}{\left(M_{1},M_{2}\right)}^{2}+d_{\text{shape}}{\left(M_{1},M_{2}\right)}^{2}},$$ (53)
  where $\lambda_{i}$ are the generalized eigenvalues defined by $M_{2}v=\lambda M_{1}v$ , and
$$d_{\text{scale}}\left(M_{1},M_{2}\right):=\left|\bar{\ln\lambda }\right|=\left|\frac{1}{n}\ln\frac{\det M_{2}}{\det M_{1}}\right|,$$ (54a)
$$d_{\text{shape}}\left(M_{1},M_{2}\right):={\left[\frac{1}{n}\overset{n}{\underset{i=1}{\sum }}{\left(\ln\lambda_{i}-\bar{\ln\lambda }\right)}^{2}\right]}^{1/2},$$ (54b)
  with det (⋅) denoting the determinant. In other words, Equations 54a, b decompose the log-spectrum underlying the AIRM distance into its mean and standard deviation, which represent the overall scale change and the anisotropic deformation, respectively. Figure 13c shows $d_{\text{scale}}$ and $d_{\text{shape}}$ between the simulated covariance Cov (Re (s)) and that obtained from the approximation, as functions of 1/σ . We observe notable scale mismatches in both the large- and small-noise regimes. The error accompanying the slow growth of the dominant mode primarily stems from the scale, whereas the shape error remains comparatively small.
- *Probability integral transform (PIT) diagnostics for truncated normality.* Although the probability distribution for ψ given by Equation 14 may be degenerate (when $B^{⊤}B$ is singular), the conditional distribution of each $\psi_{i}$ given the remaining components is still a truncated normal shown by Equation 55:
$$\psi_{i}\;|\;\psi_{j},j\neq i∼TN\left(\frac{d_{i}-\underset{j\neq i}{\sum }{\left(B^{⊤}B\right)}_{ij}\psi_{j}}{{\left(B^{⊤}B\right)}_{ii}},\;\frac{\sigma^{2}}{{\left(B^{⊤}B\right)}_{ii}};\;\psi_{i}\geq 0\right).$$ (55)
  For each sampled $\psi_{i}$ from the simulation, we then compute $u_{i}:=F_{i}\left(\psi_{i}\;|\;\psi_{j},j\neq i\right)$ , where $F_{i}$ denotes the cumulative distribution function of the truncated normal above. If the approximation were exact, the resulting $u_{i}$ would follow the uniform distribution on [0,1] for all i=1,2,…,N . Figure 13d shows the histogram of all $u_{i}$ values computed for $\psi_{i}^{H}$ at σ=0.25 . The distribution deviates from uniformity with a bias toward smaller values and a mean of 0.33. Across different i and a range of σ , the mean value of $u_{i}$ lies roughly in the interval [0.25,0.45] , indicating that the conditional truncated normality implied by the phase-decoupled approximation is not exactly followed.
- *Threshold* $\sigma_{th}$ *for stochastic oscillation death.* It is natural to expect that, as the noise level σ increases, the deterministic synchronized oscillation is eventually overwhelmed by noise and becomes invisible. In other words, the distribution of the dominant mode $z_{1}^{H}$ changes from ring-like to unimodal. Under the phase-decoupled approximation, the marginal distribution of $z_{1}^{H}$ predicts that the largest eigenvalue $d_{1}$ of W+Diag (a) should satisfy $d_{1}>d_{th}\left(d_{2},d_{3},\ldots ,d_{N};N,\sigma \right)$ for the distribution to remain ring-like; for fixed d , this yields a threshold value $\sigma_{th}$ for σ . Figure 13e shows the comparison of the theoretical threshold $1/\sigma_{th}=5.15$ with the result of Hartigan’s dip test for unimodality, which yields a p -value below 0.05 when 1/σ≳4.9 (thereby rejecting unimodality), in close agreement with the approximation. Meanwhile, the projections on other Hadamard basis vectors, such as $z_{2}^{H}$ and $z_{6}^{H}$ , always have a unimodal distribution.

Overall, our numerical investigations in the present and previous studies suggest empirically that the phase-decoupled approximation performs well quantitatively when couplings and parameter heterogeneity are weak, and the noise level is at least intermediate, namely, when Equation 56 is satisfied:

$$\frac{\rho \left(W+\text{Diag }\left(a\right)-\bar{a}I\right)}{\bar{a}}=\frac{{\left\|d-\bar{d}1\right\|}_{\infty }}{\bar{d}}≲0.05,$$ (56)

where ρ( ⋅ ) denotes the spectral radius and $\bar{a}>0$ and $\bar{d}>0$ denote the mean values of the components of a and d , respectively ( $\bar{a}=\bar{d}$ because W has zero diagonal), and Equation 57 holds:

$$\frac{\sigma }{\bar{a}}≳\sqrt{\frac{\|d-\bar{d}1{\|}_{\infty }}{\bar{d}}},\text{ or }\sigma ≳\sqrt{\bar{d}\|d-\bar{d}1{\|}_{\infty }}.$$ (57)

These results show that, although the phase-decoupled mode approximation is not exact, it yields a probability distribution that captures the statistical properties of the full system at a rough semi-quantitative level. We therefore view this approximation as a surrogate system that is energetically and statistically close to the full system, making it a plausible basis for constructing the homotopy used in the continuation.

## 4 Conclusion and discussions

### 4.1 Energy-based coupled oscillator network and its homotopy continuation

The Stuart–Landau oscillator network with homogeneous frequency and symmetric coupling provides a simple yet expressive gradient system for modeling collective dynamics from the viewpoint of its energy function. In this work, we revisited this classical setting and analyzed its energy landscape using a combination of a mode-based, phase-decoupled approximation and homotopy continuation:

- We introduced the parametric homotopy Equation 19 (and its equivalent form Equation 32) in the eigenbasis of the coupling matrix, which continuously deforms a tractable mean-field mode-amplitude system at τ=0 into the full system at τ=1 .
- We developed a systematic approach to initialize continuation at τ=0 from a singularity, arising from non-isolated equilibria in the phase subspace (after excluding the global phase-shift degree of freedom), by incorporating a second-order phase equilibrium condition.
- We numerically tracked how the approximate equilibria split into multiple equilibria of the full system, and how their energy values and indices change with the frustration strength $\kappa_{f}$ in a 2D toroidal lattice with local coupling and global frustration.
- By launching extensive continuations, we revealed a partial selectivity of our proposed homotopy: approximate solutions with relatively high (or low) energy and indices at τ=0 continue to target equilibria at τ=1 that also have relatively high (or low) energy and index.
- We identified the metastable energy landscape of the 2D lattice, at an appropriate frustration strength, as consisting of an isolated global minimum and a family of 1D energy troughs arranged along the middle portions of the edges of a multigraph, with transitions involving both the Arrhenius–Kramers and diffusion-dominated mechanisms.

In our investigation, varying the frustration strength $\kappa_{f}$ reshapes the energy landscape and hence the set of equilibria. Under weak frustration, the fully synchronized state is the unique minimum, whereas increasing $\kappa_{f}$ stabilizes some low-index saddles into additional local minima and destabilizes the fully synchronized state into a saddle. Consequently, multiple sets of isolated and non-isolated low-energy minima, including synchronized and asynchronous states, may coexist over an intermediate range of $\kappa_{f}$ . In the spirit of synergetics, these minima can be represented by ordered macroscopic patterns described by a few collective modes, while the index characterizes the stability and instability of the corresponding order parameters. Noise then induces switching between ordered states, as reflected in the changes in the dominant Hadamard modes in our simulations. These results imply that the high-dimensional coupled oscillator dynamics is effectively governed by a small set of slow collective variables, in line with the so-called slaving principle.

At the same time, several theoretical and algorithmic questions concerning the homotopy method remain open as follows:

- *Origin of selectivity.* The proposed homotopy method uses a phase-free amplitude system as the starting system, while the phase initialization relies on the conventional Newton–Raphson solution of the leading XY Hamiltonian (in a generic case) or the constrained-solvability problem (in singular cases). Compared with a purely local Newton–Raphson search in the full (r,θ) space, the homotopy method exhibits a certain selectivity, which facilitates the search for low-index equilibria. Although we intuitively attribute this selectivity to the structural closeness between the approximate and full systems, achieved by incorporating the coupling effects into the eigenspace coordinates, this selectivity is at present largely empirical, and its theoretical origin and performance remain to be clarified more rigorously.
- *Completeness of homotopy.* In homotopy continuation, a homotopy is said to be complete if, after tracking all start solutions, every isolated solution of the target system is reached by at least one continuation path. We have not yet established whether the proposed homotopy is complete in this sense, namely, whether every isolated equilibrium of the target system, or at least every low-index one, is reachable from some equilibrium of the surrogate approximation. Moreover, when W+Diag (a) has repeated eigenvalues, and $B^{⊤}B$ is singular, the equilibria found by continuation depend on the choice of eigenbasis E . Although randomization of E may exhaust the set of reachable equilibria, it remains unknown whether some equilibria are still inaccessible under this procedure. These questions call for further theoretical study of the proposed homotopy.
- *Computational complexity and scalability.* A systematic analysis of the computational complexity and scalability of the present continuation framework remains critical for the broader applicability of the method. In particular, it would be important to clarify how the computational cost, numerical stability, and equilibrium coverage vary with lattice size, and whether changes in system size introduce additional failure modes in branch tracking or correction. It would also be of interest to investigate how these aspects depend on the symmetry structure of the system as symmetry may affect both the organization of equilibria and the behavior of the continuation procedure.

All of these issues constitute important directions for future research.

### 4.2 Energy landscape analysis based on coupled oscillator models

The idea of organizing dynamics in terms of an energy landscape has a long-standing history. In statistical physics and chemistry, the potential-energy landscape framework for viscous liquids and glasses, originating with Goldstein’s barrier picture (Goldstein, 1969) and developed into the “inherent-structure” formalism (Stillinger and Weber, 1982), interprets slow relaxation as thermally activated motion among local minima separated by energy barriers (Stillinger, 1995; Debenedetti and Stillinger, 2001). In neural computation, the Amari–Hopfield neural network (Amari, 1972; Hopfield, 1982) is endowed with an energy (Lyapunov) function, whose local minima are attractors encoding stored patterns, and basins of attraction implement content-addressable recall (Hopfield, 1982). Recently, its extensions with higher-order interactions have been applied to modern machine learning tasks (Demircigil et al., 2017; Krotov and Hopfield, 2018; Ramsauer et al., 2021). In developmental and stem-cell biology, Waddington’s epigenetic landscape (Waddington, 1957) is now interpreted in dynamical-systems terms, with cell fates as attractors and fate decisions as transitions between basins, in the state space of gene regulatory networks (Kauffman, 1993; Bhattacharya et al., 2011). This picture has been made quantitative, considering quasi-potential and flux descriptions of non-equilibrium gene regulatory dynamics (Wang et al., 2011; Zhou et al., 2012) and birth-death-based decompositions into steady-state and pluripotency landscapes (Shi et al., 2022), along with data-driven methods that infer attractors and branching differentiation trajectories directly from high-dimensional single-cell transcriptomic data (Guo and Zheng, 2017; Shi et al., 2019; Zhang et al., 2019). More broadly, there is a growing trend to model disease as abnormal energy-minimum attractors and develop early-warning indicators for impending shifts from healthy to pathological states (Huang et al., 2009; Fard and Ragan, 2017; Uthamacumaran, 2021; Chen et al., 2012; Aihara et al., 2022).

In contemporary neuroscience, a prominent energy-based framework for modeling brain-wide neural dynamics, especially intrinsic or spontaneous activity, is the energy landscape analysis (Watanabe et al., 2014; Ezaki et al., 2017) implemented on the basis of a kinetic Ising model of spins (Schneidman et al., 2006; Fraiman et al., 2009; Watanabe et al., 2013). Such an Ising model can be viewed as an Amari–Hopfield network with stochastic updates (Sompolinsky, 1988), where the occurrence of binary activity patterns follows a Gibbs (Boltzmann) distribution over the energy. Energy minima thus correspond to frequently visited brain states, and thermally driven barrier crossings generate transitions among them. This approach has been applied to binarized fMRI and other population-level data to identify repertoires of brain states and their transition structure and reveal possible altered state accessibility in psychiatric and neurological disorders, including autism (Watanabe and Rees, 2017), Alzheimer’s disease (Xing et al., 2024), aphasia (Watanabe et al., 2025), and others. It should be noted that energy landscape analysis is also applicable beyond neurodynamics, for example, to the modeling of disease (Fard and Ragan, 2017). Recently, an energy landscape analysis of health checkup data clarified multiple pathways to diabetes development in obese and non-obese subjects (Ito et al., 2025).

Our key motivation for revisiting the energy-based formulation of the Stuart–Landau oscillator network is to use it as a basis for a continuous energy landscape analysis, in contrast to the popular binary approach. This perspective becomes natural when oscillatory behavior is of primary interest, and it also rests on a substantial neuroscientific background. Computational whole-brain models based on coupled oscillators, including Stuart–Landau oscillators, naturally describe collective rhythms (full and partial synchronization), multistability, metastability, and criticality. They have been successfully applied to account for brain dynamics (Deco et al., 2017; Cabral et al., 2017; Deco and Kringelbach, 2020), quantify brain flexibility (Chinichian et al., 2024), and predict disease-related alterations (Demirtaş et al., 2017; Idesis et al., 2024). We speculate that, compared with Ising-based landscapes, oscillator-based energy landscapes offer several potential advantages:

- *Continuous oscillatory states.* Oscillator networks model oscillations in continuous state variables and are suited for faithful modeling of brain signals such as fMRI and electroencephalography (EEG), as well as other oscillatory time series. In particular, they allow stable system states to be described as limit-cycle attractors, rather than static binary patterns.
- *Differential structure.* With a continuous Euclidean state space, equilibria and their stability can be characterized by standard linear spectral methods, and pathways can be treated as curves in this space. This avoids combinatorial searches over the ${\left\{\pm 1\right\}}^{N}$ hypercube and the need to analyze high-dimensional transition operators or Boolean Jacobians over $F_{2}$ .
- *Explicit noise scale.* Due to a lack of local dynamics of the node in the spin network, Ising-based approaches typically recover landscapes and couplings only relative to an overall noise level (temperature). In oscillator networks, the noise level is encoded in the diffusive wandering of trajectories and can be estimated explicitly, allowing the effects of couplings and temperature to be distinguished.

With these potential advantages, a practical workflow for energy landscape analysis based on coupled oscillator networks may parallel the now-familiar Ising-based framework (Masuda et al., 2025) while extending it to continuous oscillatory dynamics. In general, it may include the following steps:

1. Multivariate time series from the nodes of a physiological network are preprocessed by standard procedures such as normalization, detrending, and filtering, depending on the recording modality and the frequency range of interest. When the number of channels is large, clustering, parcellation, or other dimension-reduction methods may be introduced to obtain a tractable set of effective variables.
2. The processed data are then fitted with the energy-based Stuart–Landau oscillator model (Equation 1), for example, by Monte Carlo maximum likelihood estimation. In contrast to the Ising setting, this formulation allows not only the effective coupling matrix W but also the local parameters a and the noise intensity σ to be inferred from data.
3. Using the inferred W and a , one constructs the corresponding energy landscape and investigates its low-energy equilibrium structures, particularly local minima, which represent macrostates, and low-index saddles, which may mediate transitions between macrostates. The homotopy method developed in the present study is intended to contribute to this stage of the analysis.
4. The identified states may then be further analyzed in terms of their mutual distances (Equation 49), low-dimensional embeddings, and transition statistics, thereby providing a coarse-grained description of the system’s dynamical organization in a form suitable for comparison across conditions, subjects, and physiological states.

Therefore, the present study forms a preparatory step toward an oscillator-based energy landscape framework. By invoking more systematic methods for finding low-index equilibria and connecting paths (E and Zhou, 2011; Quapp and Bofill, 2014; Yin et al., 2020), a more comprehensive analysis of states and transition paths in the energy landscape of Stuart–Landau oscillator networks will become accessible, paving the way for applications to real-world systems and empirical data.

### 4.3 State transitions in energy-based models and beyond

Although a landscape with deterministic energy-descent dynamics conveniently models stable states in complex systems, it usually serves only as a diagnostic reference once we seek to describe transitions among these states because the ideal energy-descent structure is then broken. In addition to thermal activation, which yields a steady-state distribution over states, many mechanisms lead to state changes even at the deterministic level. Recently, there has been growing interest in modeling transitions among metastable states by a sequence of saddles connected by heteroclinic orbits in explicitly non-gradient systems (Afraimovich et al., 2004a; Afraimovich et al., 2004b; Rabinovich et al., 2008; Meyer-Ortmanns, 2023). Such a setting, in a more complicated form, leads to chaotic itinerancy (Tsuda, 2001; Kaneko and Tsuda, 2003; Tsuda, 2015; Inoue et al., 2020) and transitive chaotic synchronization (Kanamaru et al., 2023). Here, we can distinguish several mechanisms that induce transitions among states defined by an underlying landscape, including but not limited to the following:

- *Direct perturbations of the state.* Transitions can be caused by perturbations that act directly in the state space. One extreme is transient forcing by external inputs or pulses that push the system into another basin; the other extreme is persistent stochastic forcing that drives ongoing escapes, as in kinetic Ising models and stochastic gradient systems.
- *Non-gradient flux-driven wandering.* Here, the deterministic dynamics in the original state space is Markovian but no longer gradient: the underlying landscape still defines states, while an additional flux component introduces circulatory flow (Wang et al., 2011). Such a situation may arise from parameter inhomogeneities, asymmetric couplings (e.g., in sequential associative memory (Amari, 1972; Sompolinsky and Kanter, 1986)), or other mild non-gradient perturbations. In many models of this type, original minima become metastable waypoints that are organized by heteroclinic orbits, as mentioned above.
- *Delay-induced transitions.* When the vector field depends on time-delayed state variables, the deterministic dynamics is non-Markovian and, except in special cases, admits no finite-dimensional gradient–flux decomposition. Nonetheless, delay can break monotone descent in the underlying energy landscape and induce transitions between corresponding states (Kleinfeld, 1986; Janson and Marsden, 2021).
- *Fast or slow landscape deformation, or tipping.* In this case, the energy landscape itself changes in time with a drifting control parameter, externally prescribed or internally generated. If the parameter slowly crosses a bifurcation point, a minimum disappears or loses stability, and the system jumps to another state (bifurcation-induced tipping, B-tipping). If the parameter stays in a regime where the minimum exists but changes too fast for the system to track it, the trajectory can lose the branch and fall into a different basin (rate-induced tipping, R-tipping) (Kuehn, 2011; Ashwin et al., 2017). In particular, B-tipping is frequently adopted in modeling cell differentiation processes and disease progression (Bhattacharya et al., 2011; Aihara et al., 2022).
- *Strongly non-gradient chaotic itinerancy and transitive chaotic synchronization.* Here, the deterministic dynamics is strongly modified so that the original minima merely correspond to preferred regions (attractor ruins) in the state space, embedded in a more intricate heteroclinic skeleton than in the second case above. The resulting dynamics exhibits chaotic itinerancy and transitive chaotic synchronization: stationary epochs near one quasi-stable attractor ruin are separated by irregular transitions to others. Representative examples include the chaotic associative memory model (Adachi and Aihara, 1997; Kitajima et al., 2003), in which chaotic neurons replace spins in the Amari–Hopfield network and coupled Stuart–Landau oscillators of a more general form (Uchiyama and Fujisaka, 2004), where its equivalent Ginzburg–Landau map shows strongly nonrelaxational dynamics.

The above mechanisms can act separately or in combination in shaping state transitions. Although these mechanisms have been well studied, our interest lies in modeling and predicting real-world systems while explicitly accounting for them. Developing and generalizing oscillator-based energy landscape analysis to incorporate them may lead to more realistic and mechanistic interpretations of complex systems and remains an important direction for future work.

## Data Availability

The original contributions presented in the study are included in the article/Supplementary Material; further inquiries can be directed to the corresponding author.
