# Supplementary material for: Energy landscapes and synergetic state transitions in frustrated Stuart–Landau oscillator networks: a homotopy continuation study
Source: Front Netw Physiol. 2026 Jun 26;6:1778380. doi: 10.3389/fnetp.2026.1778380 (PMC13349764; doi:10.3389/fnetp.2026.1778380)
Supplement: Supplementary file 1 [file Supplementaryfile1.pdf]

Supplementary Appendix: Details for Homotopy Continuation

This document is the supplementary appendix for the research article “Li, Y., and Aihara, K. (2026). Energy landscapes and synergetic state transitions in frustrated Stuart–Landau oscillator networks: a homotopy continuation study. Front. Netw. Physiol. 6, 1778380. doi:10.3389/fnetp.2026.1778380”. This appendix provides detailed derivation of the explicit expressions for the homotopy system in Section 2, after introducing necessary preparatory relations in Section 1. The formulation of the second-order phase equilibrium condition as a constrained solvability problem, as well as the corresponding algorithm, is elaborated in Section 3. For clarity, the equations in this appendix are numbered consecutively after those in the main text, but with a prefix “S” to distinguish them as supplements. As the main text ends with Equation (57), this appendix begins with Equation (S58).

1 BASIC RELATIONS

The derivatives involved in the augmented coefficient matrix of the homotopy system, Equation (22) in the main text, are subtle and only become apparent upon closer examination. We specify their expressions and provide derivation here for the reader's convenient reference. For the notations, we let diag($\mathbf{M}$) denote the column vector obtained from the diagonal of a square matrix $\mathbf{M}$, and let Diag($\boldsymbol{x}$) denote the diagonal matrix constructed with a vector $\boldsymbol{x}$. In the derivation later, the identity about the Hadamard product of matrices $\mathbf{M}_1$ and $\mathbf{M}_2$,

$$\left(\mathbf{M}_1 \circ \mathbf{M}_2\right)\boldsymbol{x} = \operatorname{diag}\left(\mathbf{M}_1 \operatorname{Diag}(\boldsymbol{x})\mathbf{M}_2^\top\right), \tag{S58}$$

will be frequently used. We let $\mathbf{M}_{\cdot,k}$ and $\mathbf{M}_{k,\cdot}$ represent the kth column and kth row of a matrix $\mathbf{M}$, respectively, and let $\mathbf{M}_{*,k}$ and $\mathbf{M}_{k,*}$ represent the matrix with zero-out entries keeping only the kth column and that keeping only the kth row, respectively. We also adopt the convention that these operations precede transpose; that is, $\mathbf{M}_{k,\cdot}^\top$ represents the transpose of $\mathbf{M}_{k,\cdot}$ so that $\mathbf{M}_{k,\cdot}^\top$ is the kth column, rather than the kth row, of $\mathbf{M}^\top$. As already introduced in Sections 2.2 and 2.3 in the main text, we define quantities

$$\begin{aligned}
\mathbf{R} &= \operatorname{Diag}(\boldsymbol{r}),\\
\mathbf{V}(\boldsymbol{r}) &= \mathbf{E}^\top \operatorname{Diag}(\mathbf{B}\boldsymbol{r}^{\circ 2})\mathbf{E},\\
\mathbf{C}(\boldsymbol{\theta}) &= \left[c_{ij}(\boldsymbol{\theta})\right] = \left[\cos(\theta_i - \theta_j) - \delta_{ij}\right],\\
\mathbf{S}(\boldsymbol{\theta}) &= \left[s_{ij}(\boldsymbol{\theta})\right] = \left[\sin(\theta_i - \theta_j)\right],\\
\mathbf{G}(\boldsymbol{r},\boldsymbol{\theta}) &= \mathbf{E} \circ \left(\mathbf{E}\mathbf{R}\mathbf{C}(\boldsymbol{\theta})\right),\\
\mathbf{F}(\boldsymbol{r},\boldsymbol{\theta}) &= \mathbf{E} \circ \left(\mathbf{E}\mathbf{R}\,\mathbf{S}(\boldsymbol{\theta})\right),
\end{aligned} \tag{S59}$$

for compact expression, and later we may suppress the $\boldsymbol{r}$- and $\boldsymbol{\theta}$-dependence and simply write $\mathbf{V}$, $\mathbf{C}$, $\mathbf{S}$, $\mathbf{G}$, and $\mathbf{F}$. The matrix $\mathbf{C}$ is symmetric, while $\mathbf{S}$ is skew-symmetric, i.e., $\mathbf{S}^\top = -\mathbf{S}$. Given the definitions in Equation (8) in the main text and Equation (S59), we introduce several relations for these quantities as follows:

$$\left(\mathbf{W}^{(k)} \circ \mathbf{C}\right)\boldsymbol{r} = \operatorname{diag}\left(\mathbf{W}^{(k)}\mathbf{R}\mathbf{C}^\top\right) = \operatorname{diag}\left(\mathbf{E}_{k,\cdot}^\top \mathbf{E}_{k,\cdot}\mathbf{R}\mathbf{C}\right) = \mathbf{E}_{k,\cdot}^\top \circ \left(\mathbf{E}_{k,\cdot}\mathbf{R}\mathbf{C}\right)^\top = \mathbf{G}_{k,\cdot}^\top, \tag{S60a}$$

$$-\left(\mathbf{W}^{(k)} \circ \mathbf{S}\right)\boldsymbol{r} = -\operatorname{diag}\left(\mathbf{W}^{(k)}\mathbf{R}\mathbf{S}^\top\right) = \operatorname{diag}\left(\mathbf{E}_{k,\cdot}^\top \mathbf{E}_{k,\cdot}\mathbf{R}\mathbf{S}\right) = \mathbf{E}_{k,\cdot}^\top \circ \left(\mathbf{E}_{k,\cdot}\mathbf{R}\mathbf{S}\right)^\top = \mathbf{F}_{k,\cdot}^\top, \tag{S60b}$$

$$\left(\mathbf{W}^{(k)} \circ \mathbf{I}\right)\boldsymbol{r} = \operatorname{diag}\left(\mathbf{W}^{(k)}\mathbf{R}\right) = \operatorname{diag}\left(\mathbf{E}_{k,\cdot}^\top \mathbf{E}_{k,\cdot}\mathbf{R}\right) = \mathbf{E}_{k,\cdot}^\top \circ \left(\mathbf{E}_{k,\cdot}\mathbf{R}\right)^\top = \left(\mathbf{B}\mathbf{R}\right)_{k,\cdot}^\top, \tag{S60c}$$

all of which follow from Equation (S58). As a consequence, it is obvious that

$$\begin{bmatrix} \boldsymbol{r}^\top(\mathbf{W}^{(1)} \circ \mathbf{C}) \\ \boldsymbol{r}^\top(\mathbf{W}^{(2)} \circ \mathbf{C}) \\ \vdots \\ \boldsymbol{r}^\top(\mathbf{W}^{(N)} \circ \mathbf{C}) \end{bmatrix} = \mathbf{G}, \quad \begin{bmatrix} \boldsymbol{r}^\top(\mathbf{W}^{(1)} \circ \mathbf{S}) \\ \boldsymbol{r}^\top(\mathbf{W}^{(2)} \circ \mathbf{S}) \\ \vdots \\ \boldsymbol{r}^\top(\mathbf{W}^{(N)} \circ \mathbf{S}) \end{bmatrix} = \mathbf{F}, \quad \begin{bmatrix} \boldsymbol{r}^\top(\mathbf{W}^{(1)} \circ \mathbf{I}) \\ \boldsymbol{r}^\top(\mathbf{W}^{(2)} \circ \mathbf{I}) \\ \vdots \\ \boldsymbol{r}^\top(\mathbf{W}^{(N)} \circ \mathbf{I}) \end{bmatrix} = \mathbf{B}\mathbf{R}, \tag{S61}$$

according to Equations (S60a), (S60b), and (S60c), respectively. Moreover, we can similarly obtain

$$\begin{aligned} \left(\mathbf{V} \circ \mathbf{C}\right)\boldsymbol{r} = \operatorname{diag}\left(\mathbf{V}\mathbf{R}\mathbf{C}^\top\right) &= \operatorname{diag}\left(\mathbf{E}^\top \operatorname{Diag}(\mathbf{B}\boldsymbol{r}^{\circ 2})\mathbf{E}\mathbf{R}\mathbf{C}\right) \\ &= \left(\mathbf{E}^\top \circ (\mathbf{E}\mathbf{R}\mathbf{C})^\top\right)\mathbf{B}\boldsymbol{r}^{\circ 2} = \mathbf{G}^\top\mathbf{B}\mathbf{R}\boldsymbol{r}, \end{aligned} \tag{S62a}$$

$$\begin{aligned} -\left(\mathbf{V} \circ \mathbf{S}\right)\boldsymbol{r} = -\operatorname{diag}\left(\mathbf{V}\mathbf{R}\mathbf{S}^\top\right) &= \operatorname{diag}\left(\mathbf{E}^\top \operatorname{Diag}(\mathbf{B}\boldsymbol{r}^{\circ 2})\mathbf{E}\mathbf{R}\mathbf{S}\right) \\ &= \left(\mathbf{E}^\top \circ (\mathbf{E}\mathbf{R}\mathbf{S})^\top\right)\mathbf{B}\boldsymbol{r}^{\circ 2} = \mathbf{F}^\top\mathbf{B}\mathbf{R}\boldsymbol{r}, \end{aligned} \tag{S62b}$$

$$\begin{aligned} \left(\mathbf{V} \circ \mathbf{I}\right)\boldsymbol{r} = \operatorname{diag}\left(\mathbf{V}\mathbf{R}\right) &= \operatorname{diag}\left(\mathbf{E}^\top \operatorname{Diag}(\mathbf{B}\boldsymbol{r}^{\circ 2})\mathbf{E}\mathbf{R}\right) \\ &= \left(\mathbf{E}^\top \circ (\mathbf{E}\mathbf{R})^\top\right)\mathbf{B}\boldsymbol{r}^{\circ 2} = (\mathbf{B}\mathbf{R})^\top\mathbf{B}\mathbf{R}\boldsymbol{r}, \end{aligned} \tag{S62c}$$

by repeated application of Equation (S58).

Next, we introduce some preparatory identities involving the derivatives of the quantities defined above, which will be used frequently in the derivation later. We have elementwise derivatives

$$\frac{\partial \mathbf{G}}{\partial r_k} = \mathbf{E} \circ \left(\mathbf{E}\frac{\partial \mathbf{R}}{\partial r_k}\mathbf{C}\right) = \mathbf{E} \circ \left(\mathbf{E}_{\cdot,k}\mathbf{C}_{k,\cdot}\right) = \operatorname{Diag}(\mathbf{E}_{\cdot,k})\,\mathbf{E}\,\operatorname{Diag}(\mathbf{C}_{\cdot,k}), \tag{S63a}$$

$$\frac{\partial \mathbf{F}}{\partial r_k} = \mathbf{E} \circ \left(\mathbf{E}\frac{\partial \mathbf{R}}{\partial r_k}\mathbf{S}\right) = \mathbf{E} \circ \left(\mathbf{E}_{\cdot,k}\,\mathbf{S}_{k,\cdot}\right) = -\operatorname{Diag}(\mathbf{E}_{\cdot,k})\,\mathbf{E}\,\operatorname{Diag}(\mathbf{S}_{\cdot,k}), \tag{S63b}$$

$$\begin{aligned} \frac{\partial \mathbf{G}}{\partial \theta_k} &= \mathbf{E} \circ \left(\mathbf{E}\mathbf{R}\frac{\partial \mathbf{C}}{\partial \theta_k}\right) = -\mathbf{E} \circ \left[\mathbf{E}\mathbf{R}(\mathbf{S}_{k,*} - \mathbf{S}_{*,k})\right] \\ &= -r_k\mathbf{E} \circ \left(\mathbf{E}\mathbf{S}_{k,*}\right) + \left[\mathbf{E} \circ (\mathbf{E}\mathbf{R}\mathbf{S})_{*,k}\right] = -r_k\frac{\partial \mathbf{F}}{\partial r_k} + \mathbf{F}_{*,k}, \end{aligned} \tag{S63c}$$

$$\begin{aligned} \frac{\partial \mathbf{F}}{\partial \theta_k} &= \mathbf{E} \circ \left(\mathbf{E}\mathbf{R}\frac{\partial \mathbf{S}}{\partial \theta_k}\right) = \mathbf{E} \circ \left[\mathbf{E}\mathbf{R}(\mathbf{C}_{k,*} - \mathbf{C}_{*,k})\right] \\ &= r_k\mathbf{E} \circ \left(\mathbf{E}\mathbf{C}_{k,*}\right) - \left[\mathbf{E} \circ (\mathbf{E}\mathbf{R}\mathbf{C})_{*,k}\right] = r_k\frac{\partial \mathbf{G}}{\partial r_k} - \mathbf{G}_{*,k}. \end{aligned} \tag{S63d}$$

Using these relations, we then obtain their products with $\boldsymbol{r}$ as

$$\frac{\partial \mathbf{G}}{\partial r_k}\boldsymbol{r} = \left[\mathbf{E} \circ \left(\mathbf{E}_{\cdot,k}\mathbf{C}_{k,\cdot}\right)\right]\boldsymbol{r} = \operatorname{diag}\left(\mathbf{E}\mathbf{R}\mathbf{C}_{\cdot,k}\mathbf{E}_{\cdot,k}^\top\right) = \operatorname{diag}\left((\mathbf{E}\mathbf{R}\mathbf{C})_{\cdot,k}\mathbf{E}_{\cdot,k}^\top\right) = \mathbf{G}_{\cdot,k}, \tag{S64a}$$

$$\frac{\partial \mathbf{F}}{\partial r_k}\boldsymbol{r} = \left[\mathbf{E} \circ \left(\mathbf{E}_{\cdot,k}\,\mathbf{S}_{k,\cdot}\right)\right]\boldsymbol{r} = -\operatorname{diag}\left(\mathbf{E}\mathbf{R}\mathbf{S}_{\cdot,k}\mathbf{E}_{\cdot,k}^\top\right) = -\operatorname{diag}\left((\mathbf{E}\mathbf{R}\mathbf{S})_{\cdot,k}\mathbf{E}_{\cdot,k}^\top\right) = -\mathbf{F}_{\cdot,k}, \tag{S64b}$$

$$\frac{\partial \mathbf{G}}{\partial \theta_k}\boldsymbol{r} = -r_k\frac{\partial \mathbf{F}}{\partial r_k}\boldsymbol{r} + \mathbf{F}_{*,k}\boldsymbol{r} = -r_k\left(-\mathbf{F}_{\cdot,k}\right) + r_k\mathbf{F}_{\cdot,k} = 2r_k\mathbf{F}_{\cdot,k}, \tag{S64c}$$

$$\frac{\partial \mathbf{F}}{\partial \theta_k}\boldsymbol{r} = r_k\frac{\partial \mathbf{G}}{\partial r_k}\boldsymbol{r} - \mathbf{G}_{*,k}\boldsymbol{r} = r_k\mathbf{G}_{\cdot,k} - r_k\mathbf{G}_{\cdot,k} = \mathbf{0}, \tag{S64d}$$

after applying Equation (S58) in Equations (S64a) and (S64b) and substituting Equations (S64a) and (S64b) into Equations (S64c) and (S64d). In addition,

$$\frac{\partial (\mathbf{BR})}{\partial r_k}\boldsymbol{r} = \mathbf{B}_{*,k}\boldsymbol{r} = (\mathbf{BR})_{\cdot,k}. \tag{S65}$$

Using the relations above, we can further obtain corresponding formulas for the products involving **BR**, **G**, and **F**. This leads to

$$\begin{aligned}
\frac{\partial (\mathbf{G}^\top\mathbf{G})}{\partial r_k}\boldsymbol{r} &= \mathbf{G}^\top\frac{\partial \mathbf{G}}{\partial r_k}\boldsymbol{r} + \frac{\partial \mathbf{G}^\top}{\partial r_k}\mathbf{G}\boldsymbol{r} = \mathbf{G}^\top\mathbf{G}_{\cdot,k} + \mathrm{Diag}(\mathbf{C}_{\cdot,k})\mathbf{E}^\top\,\mathrm{Diag}(\mathbf{E}_{\cdot,k})\mathbf{G}\boldsymbol{r}\\
&= \mathbf{G}^\top\mathbf{G}_{\cdot,k} + \mathrm{Diag}(\mathbf{C}_{\cdot,k})\mathbf{E}^\top\,\mathrm{Diag}\left(\mathbf{G}\boldsymbol{r}\right)\mathbf{E}_{\cdot,k}\\
&= \mathbf{G}^\top\mathbf{G}_{\cdot,k} + \mathbf{C}_{\cdot,k}\circ\left[\mathbf{E}^\top\,\mathrm{Diag}(\mathbf{G}\boldsymbol{r})\mathbf{E}\right]_{\cdot,k},
\end{aligned} \tag{S66a}$$

$$\begin{aligned}
\frac{\partial (\mathbf{F}^\top\mathbf{G})}{\partial r_k}\boldsymbol{r} &= \mathbf{F}^\top\frac{\partial \mathbf{G}}{\partial r_k}\boldsymbol{r} + \frac{\partial \mathbf{F}^\top}{\partial r_k}\mathbf{G}\boldsymbol{r} = \mathbf{F}^\top\mathbf{G}_{\cdot,k} - \mathrm{Diag}(\mathbf{S}_{\cdot,k})\,\mathbf{E}^\top\,\mathrm{Diag}(\mathbf{E}_{\cdot,k})\mathbf{G}\boldsymbol{r}\\
&= \mathbf{F}^\top\mathbf{G}_{\cdot,k} - \mathrm{Diag}(\mathbf{S}_{\cdot,k})\mathbf{E}^\top\,\mathrm{Diag}(\mathbf{G}\boldsymbol{r})\mathbf{E}_{\cdot,k}\\
&= \mathbf{F}^\top\mathbf{G}_{\cdot,k} - \mathbf{S}_{\cdot,k}\circ\left[\mathbf{E}^\top\,\mathrm{Diag}(\mathbf{G}\boldsymbol{r})\mathbf{E}\right]_{\cdot,k},
\end{aligned} \tag{S66b}$$

$$\begin{aligned}
\frac{\partial (\mathbf{G}^\top\mathbf{BR})}{\partial r_k}\boldsymbol{r} &= \mathbf{G}^\top\frac{\partial (\mathbf{BR})}{\partial r_k}\boldsymbol{r} + \frac{\partial \mathbf{G}^\top}{\partial r_k}\mathbf{BR}\boldsymbol{r} = \mathbf{G}^\top(\mathbf{BR})_{\cdot,k} + \mathrm{Diag}(\mathbf{C}_{\cdot,k})\mathbf{E}^\top\,\mathrm{Diag}(\mathbf{E}_{\cdot,k})\mathbf{BR}\boldsymbol{r}\\
&= \mathbf{G}^\top(\mathbf{BR})_{\cdot,k} + \mathrm{Diag}(\mathbf{C}_{\cdot,k})\mathbf{E}^\top\,\mathrm{Diag}(\mathbf{B}\boldsymbol{r}^{\circ 2})\mathbf{E}_{\cdot,k}\\
&= \mathbf{G}^\top(\mathbf{BR})_{\cdot,k} + \mathbf{C}_{\cdot,k}\circ\mathbf{V}_{\cdot,k},
\end{aligned} \tag{S66c}$$

$$\begin{aligned}
\frac{\partial (\mathbf{F}^\top\mathbf{BR})}{\partial r_k}\boldsymbol{r} &= \mathbf{F}^\top\frac{\partial (\mathbf{BR})}{\partial r_k}\boldsymbol{r} + \frac{\partial \mathbf{F}^\top}{\partial r_k}\mathbf{BR}\boldsymbol{r} = \mathbf{F}^\top(\mathbf{BR})_{\cdot,k} - \mathrm{Diag}(\mathbf{S}_{\cdot,k})\mathbf{E}^\top\,\mathrm{Diag}(\mathbf{E}_{\cdot,k})\mathbf{BR}\boldsymbol{r}\\
&= \mathbf{F}^\top(\mathbf{BR})_{\cdot,k} - \mathrm{Diag}(\mathbf{S}_{\cdot,k})\mathbf{E}^\top\,\mathrm{Diag}(\mathbf{B}\boldsymbol{r}^{\circ 2})\mathbf{E}_{\cdot,k}\\
&= \mathbf{F}^\top(\mathbf{BR})_{\cdot,k} - \mathbf{S}_{\cdot,k}\circ\mathbf{V}_{\cdot,k},
\end{aligned} \tag{S66d}$$

$$\begin{aligned}
\frac{\partial \left[(\mathbf{BR})^\top\mathbf{G}\right]}{\partial r_k}\boldsymbol{r} &= \left(\mathbf{BR}\right)^\top\frac{\partial \mathbf{G}}{\partial r_k}\boldsymbol{r} + \frac{\partial (\mathbf{BR})^\top}{\partial r_k}\mathbf{G}\boldsymbol{r} = \left(\mathbf{BR}\right)^\top\mathbf{G}_{\cdot,k} + \mathbf{B}_{*,k}^\top\mathbf{G}\boldsymbol{r}\\
&= \left(\mathbf{BR}\right)^\top\mathbf{G}_{\cdot,k} + \mathrm{Diag}\left(\mathbf{B}^\top\mathbf{G}\boldsymbol{r}\right)_{\cdot,k},
\end{aligned} \tag{S66e}$$

$$\begin{aligned}
\frac{\partial \left[(\mathbf{BR})^\top\mathbf{BR}\right]}{\partial r_k}\boldsymbol{r} &= \left(\mathbf{BR}\right)^\top\frac{\partial (\mathbf{BR})}{\partial r_k}\boldsymbol{r} + \frac{\partial (\mathbf{BR})^\top}{\partial r_k}\mathbf{BR}\boldsymbol{r} = \left(\mathbf{BR}\right)^\top\left(\mathbf{BR}\right)_{\cdot,k} + \mathbf{B}_{*,k}^\top\mathbf{BR}\boldsymbol{r}\\
&= \left(\mathbf{BR}\right)^\top\left(\mathbf{BR}\right)_{\cdot,k} + \mathrm{Diag}\left(\mathbf{B}^\top\mathbf{B}\boldsymbol{r}^{\circ 2}\right)_{\cdot,k},
\end{aligned} \tag{S66f}$$

and

$$\frac{\partial(\mathbf{G}^\top\mathbf{G})}{\partial\theta_k}\boldsymbol{r} = \mathbf{G}^\top\frac{\partial\mathbf{G}}{\partial\theta_k}\boldsymbol{r} + \frac{\partial\mathbf{G}^\top}{\partial\theta_k}\mathbf{G}\boldsymbol{r} = 2r_k\mathbf{G}^\top\mathbf{F}_{\cdot,k} - r_k\frac{\partial\mathbf{F}^\top}{\partial r_k}\mathbf{G}\boldsymbol{r} + \mathbf{F}_{*,k}^\top\mathbf{G}\boldsymbol{r}$$
$$= 2r_k\mathbf{G}^\top\mathbf{F}_{\cdot,k} + r_k\operatorname{Diag}(\mathbf{S}_{\cdot,k})\mathbf{E}^\top\operatorname{Diag}(\mathbf{E}_{\cdot,k})\mathbf{G}\boldsymbol{r} + \mathbf{F}_{*,k}^\top\mathbf{G}\boldsymbol{r}$$
$$= 2r_k\mathbf{G}^\top\mathbf{F}_{\cdot,k} + r_k\mathbf{S}_{\cdot,k}\circ\left[\mathbf{E}^\top\operatorname{Diag}(\mathbf{G}\boldsymbol{r})\mathbf{E}\right]_{\cdot,k} + \operatorname{Diag}\left(\mathbf{F}^\top\mathbf{G}\boldsymbol{r}\right)_{\cdot,k}, \tag{S67a}$$

$$\frac{\partial(\mathbf{F}^\top\mathbf{G})}{\partial\theta_k}\boldsymbol{r} = \mathbf{F}^\top\frac{\partial\mathbf{G}}{\partial\theta_k}\boldsymbol{r} + \frac{\partial\mathbf{F}^\top}{\partial\theta_k}\mathbf{G}\boldsymbol{r} = 2r_k\mathbf{F}^\top\mathbf{F}_{\cdot,k} + r_k\frac{\partial\mathbf{G}^\top}{\partial r_k}\mathbf{G}\boldsymbol{r} - \mathbf{G}_{*,k}^\top\mathbf{G}\boldsymbol{r}$$
$$= 2r_k\mathbf{F}^\top\mathbf{F}_{\cdot,k} + r_k\operatorname{Diag}(\mathbf{C}_{\cdot,k})\mathbf{E}^\top\operatorname{Diag}(\mathbf{E}_{\cdot,k})\mathbf{G}\boldsymbol{r} - \mathbf{G}_{*,k}^\top\mathbf{G}\boldsymbol{r}$$
$$= 2r_k\mathbf{F}^\top\mathbf{F}_{\cdot,k} + r_k\mathbf{C}_{\cdot,k}\circ\left[\mathbf{E}^\top\operatorname{Diag}(\mathbf{G}\boldsymbol{r})\mathbf{E}\right]_{\cdot,k} - \operatorname{Diag}(\mathbf{G}^\top\mathbf{G}\boldsymbol{r})_{\cdot,k}, \tag{S67b}$$

$$\frac{\partial(\mathbf{G}^\top\mathbf{B}\mathbf{R})}{\partial\theta_k}\boldsymbol{r} = \mathbf{F}_{*,k}^\top\mathbf{B}\mathbf{R}\boldsymbol{r} - r_k\frac{\partial\mathbf{F}^\top}{\partial r_k}\mathbf{B}\mathbf{R}\boldsymbol{r} = \mathbf{F}_{*,k}^\top\mathbf{B}\mathbf{R}\boldsymbol{r} + r_k\operatorname{Diag}(\mathbf{S}_{\cdot,k})\mathbf{E}^\top\operatorname{Diag}(\mathbf{E}_{\cdot,k})\mathbf{B}\mathbf{R}\boldsymbol{r}$$
$$= r_k\mathbf{S}_{\cdot,k}\circ\mathbf{V}_{\cdot,k} + \operatorname{Diag}\left(\mathbf{F}^\top\mathbf{B}\boldsymbol{r}^{\circ 2}\right)_{\cdot,k}, \tag{S67c}$$

$$\frac{\partial(\mathbf{F}^\top\mathbf{B}\mathbf{R})}{\partial\theta_k}\boldsymbol{r} = r_k\frac{\partial\mathbf{G}^\top}{\partial r_k}\mathbf{B}\mathbf{R}\boldsymbol{r} - \mathbf{G}_{*,k}^\top\mathbf{B}\mathbf{R}\boldsymbol{r} = r_k\operatorname{Diag}(\mathbf{C}_{\cdot,k})\mathbf{E}^\top\operatorname{Diag}(\mathbf{E}_{\cdot,k})\mathbf{B}\mathbf{R}\boldsymbol{r} - \mathbf{G}_{*,k}^\top\mathbf{B}\mathbf{R}\boldsymbol{r}$$
$$= r_k\mathbf{C}_{\cdot,k}\circ\mathbf{V}_{\cdot,k} - \operatorname{Diag}\left(\mathbf{G}^\top\mathbf{B}\boldsymbol{r}^{\circ 2}\right)_{\cdot,k}, \tag{S67d}$$

$$\frac{\partial\left[(\mathbf{B}\mathbf{R})^\top\mathbf{G}\right]}{\partial\theta_k}\boldsymbol{r} = (\mathbf{B}\mathbf{R})^\top\frac{\partial\mathbf{G}}{\partial\theta_k}\boldsymbol{r} = 2r_k(\mathbf{B}\mathbf{R})^\top\mathbf{F}_{\cdot,k} = 2(\mathbf{B}\mathbf{R})^\top(\mathbf{F}\mathbf{R})_{\cdot,k}. \tag{S67e}$$

These preparatory relations will reduce the derivation later.

2 HOMOTOPY SYSTEM

Here, we present the expressions for the homotopic energy function and its derivatives, using the matrix quantities given previously in Equation (S59). The energy homotopy $H(\boldsymbol{r},\boldsymbol{\theta};\tau)$ given in Equation (19) in the main text can be re-written as

$$H(\boldsymbol{r},\boldsymbol{\theta};\tau) = \frac{1}{4}\sum_{k=1}^{N}\left[\left(\mathbf{B}\boldsymbol{r}^{\circ 2}\right)_k + \tau\boldsymbol{r}^\top\left(\mathbf{W}^{(k)}\circ\mathbf{C}(\boldsymbol{\theta})\right)\boldsymbol{r}\right]^2 - \frac{1}{2}\boldsymbol{d}^\top\boldsymbol{r}^{\circ 2}$$
$$= \frac{1}{4}\sum_{k=1}^{N}\left[(\mathbf{B}\mathbf{R}\boldsymbol{r})_k + \tau(\mathbf{G}\boldsymbol{r})_k\right]^2 - \frac{1}{2}\boldsymbol{r}^\top\operatorname{Diag}(\boldsymbol{d})\boldsymbol{r} \tag{S68}$$
$$= \frac{1}{4}\boldsymbol{r}^\top(\mathbf{B}\mathbf{R}+\tau\mathbf{G})^\top(\mathbf{B}\mathbf{R}+\tau\mathbf{G})\boldsymbol{r} - \frac{1}{2}\boldsymbol{r}^\top\operatorname{Diag}(\boldsymbol{d})\boldsymbol{r},$$

where Equations (S60a) and (S61) have been used for the second equality. In this way, the effect of the phase $\boldsymbol{\theta}$ is incorporated through the term $\tau\mathbf{G}$, which vanishes and reduces to Equation (10) in the main text when $\tau = 0$. Before dealing with its derivatives, we note the matrix identities

$$\frac{\partial}{\partial\boldsymbol{x}}\Big(\mathbf{M}(\boldsymbol{x})\boldsymbol{y}\Big) = \left[\frac{\partial\mathbf{M}(\boldsymbol{x})}{\partial x_1}\boldsymbol{y}\quad\frac{\partial\mathbf{M}(\boldsymbol{x})}{\partial x_2}\boldsymbol{y}\quad\cdots\quad\frac{\partial\mathbf{M}(\boldsymbol{x})}{\partial x_N}\boldsymbol{y}\right], \tag{S69a}$$

$$\frac{\partial}{\partial \boldsymbol{x}}\Big(\mathbf{M}(\boldsymbol{x})\boldsymbol{x}\Big) = \mathbf{M}(\boldsymbol{x}) + \left[\frac{\partial \mathbf{M}(\boldsymbol{x})}{\partial x_1}\boldsymbol{x} \quad \frac{\partial \mathbf{M}(\boldsymbol{x})}{\partial x_2}\boldsymbol{x} \quad \cdots \quad \frac{\partial \mathbf{M}(\boldsymbol{x})}{\partial x_N}\boldsymbol{x}\right], \tag{S69b}$$

for deriving Jacobian matrices. The first-order derivatives with respect to $\boldsymbol{r}$ and $\boldsymbol{\theta}$ are thus given by

$$\begin{aligned}\frac{\partial H}{\partial \boldsymbol{r}} &= \frac{1}{2}\left[\frac{\partial}{\partial \boldsymbol{r}}\Big((\mathbf{BR}+\tau\mathbf{G})\boldsymbol{r}\Big)\right]^{\top}\Big(\mathbf{BR}+\tau\mathbf{G}\Big)\boldsymbol{r} - \boldsymbol{d}\circ\boldsymbol{r} \\ &= \frac{1}{2}\Big[(\mathbf{BR}+\tau\mathbf{G}) + (\mathbf{BR}+\tau\mathbf{G})\Big]^{\top}(\mathbf{BR}+\tau\mathbf{G})\boldsymbol{r} - \boldsymbol{d}\circ\boldsymbol{r} \\ &= \Big(\mathbf{BR}+\tau\mathbf{G}\Big)^{\top}\Big(\mathbf{BR}+\tau\mathbf{G}\Big)\boldsymbol{r} - \boldsymbol{d}\circ\boldsymbol{r},\end{aligned} \tag{S70}$$

where we applied Equations (S69b), (S64a), and (S65) to obtain the second equality, and

$$\begin{aligned}\frac{\partial H}{\tau\boldsymbol{r}\partial\boldsymbol{\theta}} &= \frac{1}{2\tau}\left[\frac{\partial}{\boldsymbol{r}\partial\boldsymbol{\theta}}\Big((\mathbf{BR}+\tau\mathbf{G})\boldsymbol{r}\Big)\right]^{\top}\Big(\mathbf{BR}+\tau\mathbf{G}\Big)\boldsymbol{r} \\ &= \frac{1}{2\tau}\mathbf{R}^{-1}\big(2\tau\mathbf{FR}\big)^{\top}\big(\mathbf{BR}+\tau\mathbf{G}\big)\boldsymbol{r} = \mathbf{F}^{\top}\big(\mathbf{BR}+\tau\mathbf{G}\big)\boldsymbol{r},\end{aligned} \tag{S71}$$

where we applied Equations (S69a) and (S64c) for the second equality. Equations (S70) and (S71) provide compact expressions for writing the specific equilibrium conditions for the coupled oscillator network that we are studying. These results readily lead to the expressions for the last column in the augmented coefficient matrix in the system expressed by Equation (22) in the main text as

$$\boldsymbol{b}_1 = \frac{\partial^2 H}{\partial\tau\partial\boldsymbol{r}} = \mathbf{G}^{\top}(\mathbf{BR}+\tau\mathbf{G})\boldsymbol{r} + (\mathbf{BR}+\tau\mathbf{G})^{\top}\mathbf{G}\boldsymbol{r}, \tag{S72}$$

$$\boldsymbol{b}_2 = \frac{\partial}{\partial\tau}\frac{\partial H}{\tau\boldsymbol{r}\partial\boldsymbol{\theta}} = \mathbf{F}^{\top}\mathbf{G}\boldsymbol{r}, \tag{S73}$$

proving Equation (33b) in the main text, which was directly given without explanation.

Next, we consider the second-order derivatives with respect to $\boldsymbol{r}$ and $\boldsymbol{\theta}$. Following from Equation (S69b), we can differentiate Equation (S70) with respect to $\boldsymbol{r}$ to obtain

$$\begin{aligned}\mathbf{A}_{11} = \frac{\partial^2 H}{\partial \boldsymbol{r}^2} &= \big(\mathbf{BR}+\tau\mathbf{G}\big)^{\top}\big(\mathbf{BR}+\tau\mathbf{G}\big) - \mathrm{Diag}(\boldsymbol{d}) + \big(\mathbf{BR}\big)^{\top}\big(\mathbf{BR}\big) + \mathrm{Diag}\left(\mathbf{B}^{\top}\mathbf{B}\boldsymbol{r}^{\circ 2}\right) \\ &\quad + \tau\Big[\big(\mathbf{BR}\big)^{\top}\mathbf{G} + \mathrm{Diag}\left(\mathbf{B}^{\top}\mathbf{G}\boldsymbol{r}\right) + \mathbf{G}^{\top}(\mathbf{BR}) + \mathbf{C}\circ\mathbf{V}\Big] + \tau^2\Big[\mathbf{G}^{\top}\mathbf{G} + \mathbf{C}\circ\big(\mathbf{E}^{\top}\,\mathrm{Diag}(\mathbf{G}\boldsymbol{r})\mathbf{E}\big)\Big] \\ &= 2\left(\mathbf{BR}+\tau\mathbf{G}\right)^{\top}\big(\mathbf{BR}+\tau\mathbf{G}\big) + \mathrm{Diag}\left(\mathbf{B}^{\top}(\mathbf{BR}+\tau\mathbf{G})\boldsymbol{r} - \boldsymbol{d}\right) \\ &\quad + \tau\big(\mathbf{V}\circ\mathbf{C}\big) + \tau^2\mathbf{C}\circ\big(\mathbf{E}^{\top}\,\mathrm{Diag}(\mathbf{G}\boldsymbol{r})\mathbf{E}\big),\end{aligned} \tag{S74}$$

where we have used Equations (S66a), (S66c), (S66e), and (S66f), and differentiate Equation (S71) to obtain

$$\begin{aligned}\mathbf{A}_{21} = \frac{\partial}{\partial\boldsymbol{r}}\frac{\partial H}{\tau\boldsymbol{r}\partial\boldsymbol{\theta}} &= \frac{\partial}{\partial\boldsymbol{r}}\Big(\mathbf{F}^{\top}\big(\mathbf{BR}+\tau\mathbf{G}\big)\boldsymbol{r}\Big) \\ &= \mathbf{F}^{\top}\big(\mathbf{BR}+\tau\mathbf{G}\big) + \mathbf{F}^{\top}(\mathbf{BR}) - \mathbf{V}\circ\mathbf{S} + \tau\mathbf{F}^{\top}\mathbf{G} - \tau\mathbf{S}\circ\big(\mathbf{E}^{\top}\,\mathrm{Diag}(\mathbf{G}\boldsymbol{r})\mathbf{E}\big) \\ &= 2\mathbf{F}^{\top}\big(\mathbf{BR}+\tau\mathbf{G}\big) - \mathbf{V}\circ\mathbf{S} - \tau\mathbf{S}\circ\big(\mathbf{E}^{\top}\,\mathrm{Diag}(\mathbf{G}\boldsymbol{r})\mathbf{E}\big),\end{aligned} \tag{S75}$$

where we have used Equations (S66b) and (S66d). Following from Equation (S69a), we differentiate Equation (S70) with respect to $\boldsymbol{\theta}$ to obtain

$$\begin{aligned}\mathbf{A}_{12} = \frac{\partial^2 H}{\partial\boldsymbol{\theta}\partial\boldsymbol{r}} &= \frac{\partial}{\partial\boldsymbol{\theta}}\Big(\big(\mathbf{BR}+\tau\mathbf{G}\big)^\top\big(\mathbf{BR}+\tau\mathbf{G}\big)\boldsymbol{r}\Big)\\ &= 2\tau\big(\mathbf{BR}\big)^\top\big(\mathbf{FR}\big) + \tau\big(\mathbf{V}\circ\mathbf{S}\big)\mathbf{R} + \tau\operatorname{Diag}\left(\mathbf{F}^\top\mathbf{B}\boldsymbol{r}^{\circ 2}\right)\\ &\quad + 2\tau^2\mathbf{G}^\top\mathbf{FR} + \tau^2\Big[\mathbf{S}\circ\left(\mathbf{E}^\top\operatorname{Diag}(\mathbf{G}\boldsymbol{r})\mathbf{E}\right)\Big]\mathbf{R} + \tau^2\operatorname{Diag}\left(\mathbf{F}^\top\mathbf{G}\boldsymbol{r}\right)\\ &= 2\tau\big(\mathbf{BR}+\tau\mathbf{G}\big)^\top\mathbf{FR} + \tau\operatorname{Diag}\left(\mathbf{F}^\top(\mathbf{BR}+\tau\mathbf{G})\boldsymbol{r}\right)\\ &\quad + \tau\big(\mathbf{V}\circ\mathbf{S}\big)\mathbf{R} + \tau^2\Big[\mathbf{S}\circ\left(\mathbf{E}^\top\operatorname{Diag}(\mathbf{G}\boldsymbol{r})\mathbf{E}\right)\Big]\mathbf{R},\end{aligned} \tag{S76}$$

where we have used Equations (S67a), (S67c), and (S67e), and we differentiate Equation (S71) to obtain

$$\begin{aligned}\mathbf{A}_{22} &= \frac{\partial}{\partial\boldsymbol{\theta}}\frac{\partial H}{\tau\boldsymbol{r}\partial\boldsymbol{\theta}} = \frac{\partial}{\partial\boldsymbol{\theta}}\left(\mathbf{F}^\top(\mathbf{BR}+\tau\mathbf{G})\boldsymbol{r}\right)\\ &= \big(\mathbf{V}\circ\mathbf{C}\big)\mathbf{R} - \operatorname{Diag}\left(\mathbf{G}^\top\mathbf{B}\boldsymbol{r}^{\circ 2}\right) + 2\tau\mathbf{F}^\top\mathbf{FR} + \tau\Big[\mathbf{C}\circ\left(\mathbf{E}^\top\operatorname{Diag}(\mathbf{G}\boldsymbol{r})\mathbf{E}\right)\Big]\mathbf{R} - \tau\operatorname{Diag}(\mathbf{G}^\top\mathbf{G}\boldsymbol{r})\\ &= \big(\mathbf{V}\circ\mathbf{C}\big)\mathbf{R} - \operatorname{Diag}\left(\mathbf{G}^\top(\mathbf{BR}+\tau\mathbf{G})\boldsymbol{r}\right) + 2\tau\mathbf{F}^\top\mathbf{FR} + \tau\Big[\mathbf{C}\circ\left(\mathbf{E}^\top\operatorname{Diag}(\mathbf{G}\boldsymbol{r})\mathbf{E}\right)\Big]\mathbf{R},\end{aligned} \tag{S77}$$

where we have used Equations (S67b) and (S67d). Now, all blocks in the coefficient matrix have been specified as given in Equation (34) in the main text.

Finally, we specify the Hessian matrix of the energy homotopy in Equation (19) in the main text, which, when evaluated at a critical point, characterizes its stability. In particular, it describes the stability of equilibria in the approximate mode-amplitude system at $\tau = 0$ and that of equilibria in the coupled oscillator network at $\tau = 1$. Similar to Equation (6) in the main text, the Hessian in the $(\boldsymbol{r}, \boldsymbol{\theta})$-coordinates is given by

$$\nabla^2 H(\boldsymbol{r},\boldsymbol{\theta};\tau) = \begin{bmatrix} \dfrac{\partial^2 H}{\partial\boldsymbol{r}^2} & \dfrac{\partial^2 H}{\boldsymbol{r}\partial\boldsymbol{\theta}\partial\boldsymbol{r}} - \operatorname{Diag}\left(\dfrac{\partial H}{\boldsymbol{r}^2\partial\boldsymbol{\theta}}\right)\\ \dfrac{\partial^2 H}{\boldsymbol{r}\partial\boldsymbol{r}\partial\boldsymbol{\theta}} - \operatorname{Diag}\left(\dfrac{\partial H}{\boldsymbol{r}^2\partial\boldsymbol{\theta}}\right) & \dfrac{\partial^2 H}{\boldsymbol{r}^2\partial\boldsymbol{\theta}^2} + \operatorname{Diag}\left(\dfrac{\partial H}{\boldsymbol{r}\partial\boldsymbol{r}}\right)\end{bmatrix}. \tag{S78}$$

We can derive the following two relations:

$$\begin{aligned}&\frac{\partial^2 H}{\boldsymbol{r}\partial\boldsymbol{\theta}\partial\boldsymbol{r}} - \operatorname{Diag}\left(\frac{\partial H}{\boldsymbol{r}^2\partial\boldsymbol{\theta}}\right)\\ &= \tau(\mathbf{V}\circ\mathbf{S}) - \tau\operatorname{Diag}\left((\mathbf{V}\circ\mathbf{S})\boldsymbol{r}\right)\mathbf{R}^{-1} + 2\tau\big(\mathbf{BR}\big)^\top + 2\tau^2\mathbf{G}^\top\mathbf{F} + \tau^2\mathbf{S}\circ\left(\mathbf{E}^\top\operatorname{Diag}(\mathbf{G}\boldsymbol{r})\mathbf{E}\right)\\ &\quad + \tau^2\operatorname{Diag}\left(\mathbf{F}^\top\mathbf{G}\boldsymbol{r}\right)\mathbf{R}^{-1} + \tau\operatorname{Diag}\left((\mathbf{V}\circ\mathbf{S})\boldsymbol{r}\right)\mathbf{R}^{-1} - \tau^2\operatorname{Diag}\left(\mathbf{F}^\top\mathbf{G}\boldsymbol{r}\right)\mathbf{R}^{-1}\\ &= \tau(\mathbf{V}\circ\mathbf{S}) + 2\tau\big(\mathbf{BR}\big)^\top\mathbf{F} + 2\tau^2\mathbf{G}^\top\mathbf{F} + \tau^2\mathbf{S}\circ\left(\mathbf{E}^\top\operatorname{Diag}(\mathbf{G}\boldsymbol{r})\mathbf{E}\right),\end{aligned} \tag{S79}$$

$$
\begin{aligned}
&\frac{\partial^2 H}{\boldsymbol{r}^2 \partial \boldsymbol{\theta}^2} + \operatorname{Diag}\left(\frac{\partial H}{\boldsymbol{r}\partial \boldsymbol{r}}\right) \\
&= \tau\big(\mathbf{V}\circ\mathbf{C}\big) - \tau \operatorname{Diag}\big((\mathbf{V}\circ\mathbf{C})\boldsymbol{r}\big)\mathbf{R}^{-1} + 2\tau^2\mathbf{F}^\top\mathbf{F} + \tau^2\mathbf{C}\circ\big(\mathbf{E}^\top\operatorname{Diag}(\mathbf{G}\boldsymbol{r})\mathbf{E}\big) - \tau^2\operatorname{Diag}(\mathbf{G}^\top\mathbf{G}\boldsymbol{r})\mathbf{R}^{-1} \\
&\quad + \operatorname{Diag}\big(\mathbf{B}^\top\mathbf{B}\boldsymbol{r}^{\circ 2} - \boldsymbol{d}\big) + \tau\operatorname{Diag}\big((\mathbf{V}\circ\mathbf{C})\boldsymbol{r}\big)\mathbf{R}^{-1} + \tau\operatorname{Diag}\big((\mathbf{B}\mathbf{R})^\top\mathbf{G}\boldsymbol{r}\big)\mathbf{R}^{-1} + \tau^2\operatorname{Diag}\big(\mathbf{G}^\top\mathbf{G}\boldsymbol{r}\big)\mathbf{R}^{-1} \\
&= \operatorname{Diag}\big(\mathbf{B}^\top\mathbf{B}\boldsymbol{r}^{\circ 2} - \boldsymbol{d}\big) + \tau\big(\mathbf{V}\circ\mathbf{C}\big) + \tau\operatorname{Diag}\big(\mathbf{B}^\top\mathbf{G}\boldsymbol{r}\big) + 2\tau^2\mathbf{F}^\top\mathbf{F} + \tau^2\mathbf{C}\circ\big(\mathbf{E}^\top\operatorname{Diag}(\mathbf{G}\boldsymbol{r})\mathbf{E}\big).
\end{aligned}
\tag{S80}
$$

Therefore, the Hessian matrix for the energy homotopy is given by

$$
\begin{aligned}
\nabla^2 H(\boldsymbol{r},\boldsymbol{\theta};\tau) &= \begin{bmatrix} \operatorname{Diag}\big(\mathbf{B}^\top\mathbf{B}\boldsymbol{r}^{\circ 2} - \boldsymbol{d}\big) + 2\mathbf{R}\mathbf{B}^\top\mathbf{B}\mathbf{R} & \mathbf{0} \\ \mathbf{0} & \operatorname{Diag}\big(\mathbf{B}^\top\mathbf{B}\boldsymbol{r}^{\circ 2} - \boldsymbol{d}\big) \end{bmatrix} \\
&+ \tau \begin{bmatrix} \mathbf{V}\circ\mathbf{C} + \operatorname{Diag}\big(\mathbf{B}^\top\mathbf{G}\boldsymbol{r}\big) + 2\mathbf{G}^\top\mathbf{B}\mathbf{R} + 2(\mathbf{B}\mathbf{R})^\top\mathbf{G} & \mathbf{V}\circ\mathbf{S} + 2\big(\mathbf{B}\mathbf{R}\big)^\top\mathbf{F} \\ -\mathbf{V}\circ\mathbf{S} + 2\mathbf{F}^\top\mathbf{B}\mathbf{R} & \mathbf{V}\circ\mathbf{C} + \operatorname{Diag}\big(\mathbf{B}^\top\mathbf{G}\boldsymbol{r}\big) \end{bmatrix} \\
&+ \tau^2 \begin{bmatrix} \mathbf{C}\circ\big(\mathbf{E}^\top\operatorname{Diag}(\mathbf{G}\boldsymbol{r})\mathbf{E}\big) + 2\mathbf{G}^\top\mathbf{G} & \mathbf{S}\circ\big(\mathbf{E}^\top\operatorname{Diag}(\mathbf{G}\boldsymbol{r})\mathbf{E}\big) + 2\mathbf{G}^\top\mathbf{F} \\ -\mathbf{S}\circ\big(\mathbf{E}^\top\operatorname{Diag}(\mathbf{G}\boldsymbol{r})\mathbf{E}\big) + 2\mathbf{F}^\top\mathbf{G} & \mathbf{C}\circ\big(\mathbf{E}^\top\operatorname{Diag}(\mathbf{G}\boldsymbol{r})\mathbf{E}\big) + 2\mathbf{F}^\top\mathbf{F} \end{bmatrix}.
\end{aligned}
\tag{S81}
$$

3 CONSTRAINED SOLVABILITY

Here we introduce the constrained solvability problem, which is used to determine feasible initial phases $\boldsymbol{\theta}_0$ in the presence of additional singularity as described in Section 2.4 in the main text. First, we present the problem in a general setting: for the parameter-dependent linear system

$$
\mathbf{A}(\boldsymbol{\theta})\,\boldsymbol{y} + \boldsymbol{b}(\boldsymbol{\theta}) = \mathbf{0}, \tag{S82}
$$

where $\mathbf{A}(\boldsymbol{\theta}) : \mathbb{R}^n \to \mathbb{R}^{n\times n}$ and $\boldsymbol{b}(\boldsymbol{\theta}) : \mathbb{R}^n \to \mathbb{R}^n$, find $\boldsymbol{\theta}$ constrained to a manifold $\boldsymbol{c}(\boldsymbol{\theta}) = \mathbf{0} \in \mathbb{R}^n$ such that Equation (S82) is solvable for $\boldsymbol{y}$. We assume that

$$
\frac{\partial \boldsymbol{c}(\boldsymbol{\theta})}{\partial \boldsymbol{\theta}} = \mathbf{A}(\boldsymbol{\theta}), \tag{S83}
$$

and that the rank of $\mathbf{A}(\boldsymbol{\theta})$ is locally constant in the neighborhood of interest. To characterize solvability, we define a function $g(\boldsymbol{\theta}) : \mathbb{R}^n \to \mathbb{R}$ by

$$
g(\boldsymbol{\theta}) \coloneqq \frac{1}{2}\min_{\boldsymbol{y}\in\mathbb{R}^n} \big\|\mathbf{A}(\boldsymbol{\theta})\,\boldsymbol{y} + \boldsymbol{b}(\boldsymbol{\theta})\big\|^2, \tag{S84}
$$

and define the associated residual function $\mathbf{f}(\boldsymbol{\theta}) : \mathbb{R}^n \to \mathbb{R}^n$ by

$$
\mathbf{f}(\boldsymbol{\theta}) \coloneqq \mathbf{A}(\boldsymbol{\theta})\,\boldsymbol{y}_*(\boldsymbol{\theta}) + \boldsymbol{b}(\boldsymbol{\theta}), \tag{S85}
$$

where $\boldsymbol{y}_*(\boldsymbol{\theta})$ denotes any least-squares solution with a given $\boldsymbol{\theta}$ (the residual $\mathbf{f}(\boldsymbol{\theta})$ is unique even if the least-squares solution is not). Then, Equation (S82) is solvable if and only if $g(\boldsymbol{\theta}) = 0$ (equivalently, $\mathbf{f}(\boldsymbol{\theta}) = \mathbf{0}$). The original constrained solvability problem is therefore reformulated as solving the coupled system $\mathbf{f}(\boldsymbol{\theta}) = \mathbf{0}$ and $\boldsymbol{c}(\boldsymbol{\theta}) = \mathbf{0}$.

Next, we present an iterative method for solving the preceding coupled system. Under the locally constant-rank assumption of $\mathbf{A}(\boldsymbol{\theta})$, the least-squares residual $\mathbf{f}(\boldsymbol{\theta})$ is well-defined and varies smoothly in the neighborhood of interest. Differentiating $g(\boldsymbol{\theta})$ for each component θ_i, we find

$$\begin{aligned}\frac{\partial g(\boldsymbol{\theta})}{\partial \theta_i} &= \frac{1}{2}\frac{\partial}{\partial \theta_i}\Big[\mathbf{f}(\boldsymbol{\theta})^\top \mathbf{f}(\boldsymbol{\theta})\Big] = \mathbf{f}(\boldsymbol{\theta})^\top \frac{\partial \mathbf{f}(\boldsymbol{\theta})}{\partial \theta_i} \\ &= \mathbf{f}(\boldsymbol{\theta})^\top \left[\frac{\partial \mathbf{A}(\boldsymbol{\theta})}{\partial \theta_i}\boldsymbol{y}_*(\boldsymbol{\theta}) + \mathbf{A}(\boldsymbol{\theta})\frac{\partial \boldsymbol{y}_*(\boldsymbol{\theta})}{\partial \theta_i} + \frac{\partial \boldsymbol{b}(\boldsymbol{\theta})}{\partial \theta_i}\right] \\ &= \mathbf{f}(\boldsymbol{\theta})^\top \left[\frac{\partial \mathbf{A}(\boldsymbol{\theta})}{\partial \theta_i}\boldsymbol{y}_*(\boldsymbol{\theta}) + \frac{\partial \boldsymbol{b}(\boldsymbol{\theta})}{\partial \theta_i}\right],\end{aligned} \tag{S86}$$

where the last equality follows from $\mathbf{A}(\boldsymbol{\theta})^\top \mathbf{f}(\boldsymbol{\theta}) = \mathbf{0}$; that is, the residual must be orthogonal to the column space of $\mathbf{A}(\boldsymbol{\theta})$. Defining $\mathbf{K}(\boldsymbol{\theta}) \in \mathbf{R}^{n\times n}$ by stacking the column vectors in the last square brackets for $i = 1, 2, \cdots, n$, Equation (S86) can be written simply as

$$\frac{\partial g(\boldsymbol{\theta})}{\partial \boldsymbol{\theta}} = \mathbf{K}(\boldsymbol{\theta})^\top \mathbf{f}(\boldsymbol{\theta}). \tag{S87}$$

Using $\mathbf{K}(\boldsymbol{\theta})^\top \mathbf{K}(\boldsymbol{\theta})$ as a positive semidefinite Hessian approximation, we now build a Gauss–Newton-type local model for $g(\boldsymbol{\theta})$, as

$$g(\boldsymbol{\theta} + \delta\boldsymbol{\theta}) \approx g(\boldsymbol{\theta}) + \delta\boldsymbol{\theta}^\top \mathbf{K}(\boldsymbol{\theta})^\top \mathbf{f}(\boldsymbol{\theta}) + \frac{1}{2}\delta\boldsymbol{\theta}^\top \mathbf{K}(\boldsymbol{\theta})^\top \mathbf{K}(\boldsymbol{\theta})\delta\boldsymbol{\theta} = \frac{1}{2}\big\|\mathbf{f}(\boldsymbol{\theta}) + \mathbf{K}(\boldsymbol{\theta})\delta\boldsymbol{\theta}\big\|^2. \tag{S88}$$

Meanwhile, in order to stay on the manifold $\boldsymbol{c}(\boldsymbol{\theta}) = \mathbf{0}$, we adopt the first-order approximation $\boldsymbol{c}(\boldsymbol{\theta} + \delta\boldsymbol{\theta}) \approx \boldsymbol{c}(\boldsymbol{\theta}) + \mathbf{A}(\boldsymbol{\theta})\delta\boldsymbol{\theta}$ and impose the linearized constraint

$$\mathbf{A}(\boldsymbol{\theta})\delta\boldsymbol{\theta} = -\boldsymbol{c}(\boldsymbol{\theta}). \tag{S89}$$

Therefore, in each iteration for solving the original constrained solvability problem, we solve the constrained least-squares subproblem

$$\min_{\delta\boldsymbol{\theta}} \frac{1}{2}\big\|\mathbf{f}(\boldsymbol{\theta}) + \mathbf{K}(\boldsymbol{\theta})\delta\boldsymbol{\theta}\big\|^2 \quad \text{s.t.} \quad \mathbf{A}(\boldsymbol{\theta})\delta\boldsymbol{\theta} = -\boldsymbol{c}(\boldsymbol{\theta}), \tag{S90}$$

whose first-order optimality conditions yield the KKT system

$$\begin{bmatrix} \mathbf{K}(\boldsymbol{\theta})^\top \mathbf{K}(\boldsymbol{\theta}) & \mathbf{A}(\boldsymbol{\theta})^\top \\ \mathbf{A}(\boldsymbol{\theta}) & \mathbf{0} \end{bmatrix} \begin{bmatrix} \delta\boldsymbol{\theta} \\ \boldsymbol{\mu} \end{bmatrix} = - \begin{bmatrix} \mathbf{K}(\boldsymbol{\theta})^\top \mathbf{f}(\boldsymbol{\theta}) \\ \boldsymbol{c}(\boldsymbol{\theta}) \end{bmatrix}, \tag{S91}$$

where $\boldsymbol{\mu}$ is the Lagrange multiplier associated with Equation (S89). It is easy to verify that the $\delta\boldsymbol{\theta}$ returned by the KKT system corresponds to a descent direction for the augmented merit function

$$\Phi(\boldsymbol{\theta}, \boldsymbol{\mu}) := g(\boldsymbol{\theta}) + \boldsymbol{\mu}^\top \boldsymbol{c}(\boldsymbol{\theta}) + \frac{\nu}{2}\left\|\boldsymbol{c}(\boldsymbol{\theta})\right\|^2, \quad \nu > 0, \tag{S92}$$

unless a KKT stationary point of $\min_{\boldsymbol{\theta}} g(\boldsymbol{\theta})$ subject to $\boldsymbol{c}(\boldsymbol{\theta}) = \mathbf{0}$ has been reached. Indeed, using Equations (S87) and (S91), we have

$$\delta\boldsymbol{\theta}^\top \frac{\partial \Phi(\boldsymbol{\theta},\boldsymbol{\mu})}{\partial \boldsymbol{\theta}} = \delta\boldsymbol{\theta}^\top \left[\mathbf{K}(\boldsymbol{\theta})^\top \mathbf{f}(\boldsymbol{\theta}) + \mathbf{A}(\boldsymbol{\theta})^\top \boldsymbol{\mu} + \nu \mathbf{A}(\boldsymbol{\theta})^\top \boldsymbol{c}(\boldsymbol{\theta})\right] \\ = \delta\boldsymbol{\theta}^\top \left[-\mathbf{K}(\boldsymbol{\theta})^\top \mathbf{K}(\boldsymbol{\theta})\delta\boldsymbol{\theta} + \nu \mathbf{A}(\boldsymbol{\theta})^\top \boldsymbol{c}(\boldsymbol{\theta})\right] = -\left\|\mathbf{K}(\boldsymbol{\theta})\delta\boldsymbol{\theta}\right\|^2 - \nu \left\|\boldsymbol{c}(\boldsymbol{\theta})\right\|^2 \le 0, \tag{S93}$$

with equality only if $\boldsymbol{c}(\boldsymbol{\theta}) = \mathbf{0}$ and $\mathbf{K}(\boldsymbol{\theta})\delta\boldsymbol{\theta} = \mathbf{0}$ (equivalently, $\mathbf{A}(\boldsymbol{\theta})^\top + \partial\Phi(\boldsymbol{\theta},\boldsymbol{\mu})/\partial\boldsymbol{\theta} = \mathbf{0}$). Therefore, the iteration can be globalized by performing Armijo backtracking on the merit function, to choose a step size $\alpha \in (0,1]$ such that $\Phi(\boldsymbol{\theta} + \alpha\delta\boldsymbol{\theta}, \boldsymbol{\mu})$ decreases sufficiently.

Finally, we present the expressions for related quantities in our problem, especially, the matrix $\mathbf{K}(\boldsymbol{\theta})$. By defining

$$\boldsymbol{x}_*(\boldsymbol{\theta}) \coloneqq -\mathbf{A}_{11}^{(0)-1} \boldsymbol{b}_1^{(0)}(\boldsymbol{\theta}) \tag{S94}$$

and referring to Equation (40) in Section 2.4 in the main text, quantities $\mathbf{A}(\boldsymbol{\theta})$, $\boldsymbol{b}(\boldsymbol{\theta})$, and $\boldsymbol{y}_*(\boldsymbol{\theta})$ in the general setting above obviously have the following correspondence:

$$\mathbf{A}(\boldsymbol{\theta}) \leftarrow \mathbf{A}_{22}^{(0)}(\boldsymbol{\theta}), \tag{S95a}$$

$$\boldsymbol{b}(\boldsymbol{\theta}) \leftarrow \boldsymbol{b}_2^{(0)}(\boldsymbol{\theta}) + \mathbf{A}_{21}^{(0)}(\boldsymbol{\theta})\boldsymbol{x}_*(\boldsymbol{\theta}), \tag{S95b}$$

$$\boldsymbol{y}_*(\boldsymbol{\theta}) \leftarrow -\mathbf{A}_{22}^{(0)+}(\boldsymbol{\theta})\left[\boldsymbol{b}_2^{(0)}(\boldsymbol{\theta}) + \mathbf{A}_{21}^{(0)}(\boldsymbol{\theta})\boldsymbol{x}_*(\boldsymbol{\theta})\right], \tag{S95c}$$

where $\mathbf{A}_{22}^{(0)+}$ denotes the pseudoinverse of $\mathbf{A}_{22}^{(0)}$, while $\boldsymbol{b}_2^{(0)}$, $\boldsymbol{b}_1^{(0)}$, $\mathbf{A}_{22}^{(0)}$, $\mathbf{A}_{21}^{(0)}$, and $\mathbf{A}_{11}^{(0)}$ have been specified in Equations (33b) and (34) in the main text. From the definition of $\mathbf{K}(\boldsymbol{\theta})$ in Equations (S86) and (S87), its kth column is given by

$$\frac{\partial \mathbf{A}(\boldsymbol{\theta})}{\partial \theta_k}\boldsymbol{y}_*(\boldsymbol{\theta}) + \frac{\partial \boldsymbol{b}(\boldsymbol{\theta})}{\partial \theta_k} \\ \leftarrow \frac{\partial \mathbf{A}_{22}^{(0)}(\boldsymbol{\theta})}{\partial \theta_k}\boldsymbol{y}_*(\boldsymbol{\theta}) + \frac{\partial \boldsymbol{b}_2^{(0)}(\boldsymbol{\theta})}{\partial \theta_k} + \frac{\partial \mathbf{A}_{21}^{(0)}(\boldsymbol{\theta})}{\partial \theta_k}\boldsymbol{x}_*(\boldsymbol{\theta}) - \mathbf{A}_{21}^{(0)}(\boldsymbol{\theta})\mathbf{A}_{11}^{(0)-1}\frac{\partial \boldsymbol{b}_1^{(0)}(\boldsymbol{\theta})}{\partial \theta_k}. \tag{S96}$$

We can clearly see

$$\frac{\partial}{\partial \boldsymbol{\theta}}\boldsymbol{b}_1^{(0)}(\boldsymbol{\theta}) = \left.\frac{\partial^2 H_1(\boldsymbol{r},\boldsymbol{\theta})}{\partial\boldsymbol{\theta}\partial\boldsymbol{r}}\right|_{\boldsymbol{r}=\boldsymbol{r}_0} = \mathbf{A}_{12}^{(1)}(\boldsymbol{\theta}), \tag{S97a}$$

$$\frac{\partial}{\partial \boldsymbol{\theta}}\boldsymbol{b}_2^{(0)}(\boldsymbol{\theta}) = \frac{\partial}{\boldsymbol{r}_0\partial\boldsymbol{\theta}}H_2(\boldsymbol{r}_0,\boldsymbol{\theta}) = \mathbf{A}_{22}^{(1)}(\boldsymbol{\theta}), \tag{S97b}$$

by comparing the definitions of related quantities and their expansions in τ. Moreover, we can derive

$$\frac{\partial \mathbf{A}_{21}^{(0)}}{\partial \theta_k} = \frac{\partial\left[-\mathbf{V}\circ\mathbf{S} + 2\mathbf{F}^\top\mathbf{B}\mathbf{R}\right]}{\partial \theta_k} \\ = -\mathbf{V}\circ(\mathbf{C}_{k,*} - \mathbf{C}_{*,k}) + 2r_k \operatorname{Diag}(\mathbf{C}_{\cdot,k})\mathbf{E}^\top \operatorname{Diag}(\mathbf{E}_{\cdot,k})\mathbf{B}\mathbf{R} - 2\mathbf{G}_{*,k}^\top\mathbf{B}\mathbf{R}, \tag{S98}$$

$$\frac{\partial \mathbf{A}_{22}^{(0)}}{\partial \theta_k} = \frac{\partial\left[(\mathbf{V}\circ\mathbf{C})\mathbf{R} - \operatorname{Diag}\left(\mathbf{G}^\top\mathbf{B}\mathbf{R}\boldsymbol{r}\right)\right]}{\partial \theta_k} \\ = -\big(\mathbf{V}\circ(\mathbf{S}_{k,*} - \mathbf{S}_{*,k})\big)\mathbf{R} + \operatorname{Diag}\left[\big(\mathbf{V}\circ(\mathbf{S}_{k,*} - \mathbf{S}_{*,k})\big)\boldsymbol{r}\right], \tag{S99}$$

where all quantities should be evaluated at $\boldsymbol{r} = \boldsymbol{r}_0$. Therefore, the matrix $\mathbf{K}(\boldsymbol{\theta})$ can be expressed as the sum of two parts,

$$\mathbf{K}(\boldsymbol{\theta}) = \mathbf{K}^1(\boldsymbol{\theta}) + \mathbf{K}^2(\boldsymbol{\theta}), \tag{S100a}$$

$$\mathbf{K}^1(\boldsymbol{\theta}) = \mathbf{A}_{22}^{(1)}(\boldsymbol{\theta}) - \mathbf{A}_{21}^{(0)}(\boldsymbol{\theta})\mathbf{A}_{11}^{(0)-1}\mathbf{A}_{12}^{(1)}(\boldsymbol{\theta}), \tag{S100b}$$

$$\mathbf{K}^2_{\cdot,k}(\boldsymbol{\theta}) = \frac{\partial \mathbf{A}_{22}^{(0)}(\boldsymbol{\theta})}{\partial \theta_k}\boldsymbol{y}_*(\boldsymbol{\theta}) + \frac{\partial \mathbf{A}_{21}^{(0)}(\boldsymbol{\theta})}{\partial \theta_k}\boldsymbol{x}_*(\boldsymbol{\theta}), \tag{S100c}$$

with $\partial \mathbf{A}_{22}^{(0)}/\partial \theta_k$ and $\partial \mathbf{A}_{21}^{(0)}/\partial \theta_k$ given by Equations (S98) and (S99). The appendix ends here.
